# Enhanced biosynthesis of full-length triple-helical human type III collagen in engineered *Komagataella phaffii*

**DOI:** 10.1007/s00253-026-13996-7

**Published:** 2026-08-29

**Authors:** Zhijian Ni, Xiaolu Yu, Qi Da, Xiaoxi Wu, ZhiDa Wei, Yulei Hu, Yao Xiao, Chunting Ye, Chunlin Deng

**Affiliations:** 1 School of Materials Science and Engineering, South China University of Technology, Guangzhou, 510641 China; 2 Guangzhou Trauer Biotechnology Co., Ltd., Guangzhou, 510000 China; 3 School of Bioscience and Bioengineering, Jiangxi Agricultural University, Nanchang, 330045 China; 4 National Engineering Research Center for Tissue Restoration and Reconstruction, Guangzhou, 510006 China

**Keywords:** Human type III collagen, *Komagataella phafi*, Prolyl 4-hydroxylase, Procollagen trimer, Triple helix, Characterization

## Abstract

**Abstract:**

Fibrillar type III collagen, characterized by a distinct triple helix structure, is a critical structural component of the mammalian extracellular matrix (ECM). Its unique mechanical properties and biological functions make it a highly valuable biomaterial for medical applications. However, the limited availability of natural human type III collagen (hCOL3) and the defects of animal-derived sources necessitate sustainable alternatives. Here, we developed an integrated molecular engineering strategy for the efficient production of full-length recombinant human type III collagen (rhCOL3) in *Komagataella phaffii*. The N- and C-terminal propeptides of the rhCOL3α1 chain were rationally redesigned to preserve its intrinsic capability for triple helix formation. Synergistic co-expression of human P4Hαβ and *Bacillus anthracis*-derived BaP4H achieved a proline hydroxylation level of 52.88%, enabling the stable assembly of rhCOL3 procollagen homotrimers (> 300 kDa). Subsequent optimization, including high-copy-number strain screening, signal sequence substitution (with Ost1 identified as optimal), and co-expression of the molecular chaperones (e.g., BCY1 and ERO1), significantly enhanced productivity. The engineered strain achieved a rhCOL3 procollagen yield of 430 mg/L in a 5-L fed-batch bioreactor. The purified rhCOL3 exhibited a stable triple helix conformation, a melting temperature of 38.87 °C, and the ability to self-assemble into fibrils with a characteristic D-banding pattern. Biological assays confirmed that the rhCOL3 promotes cell proliferation, adhesion, and migration at levels comparable to those of natural collagen. This study establishes a promising platform for the industrial-scale production of bioactive, triple-helical human collagen.

**Key points:**

• *Synergistic P4Hαβ and BaP4H co-expression achieves 52.88% Hyp level and Tm 38.87 °C.*

• *Ost1 and Bcy1 co-expression elevated rhCOL3 yield to 430 mg/L in a 5-L bioreactor.*

• *Purified rhCOL3 self-assembles into D-banded fibrils resembling native collagen.*

**Graphical Abstract:**

**Figure:**
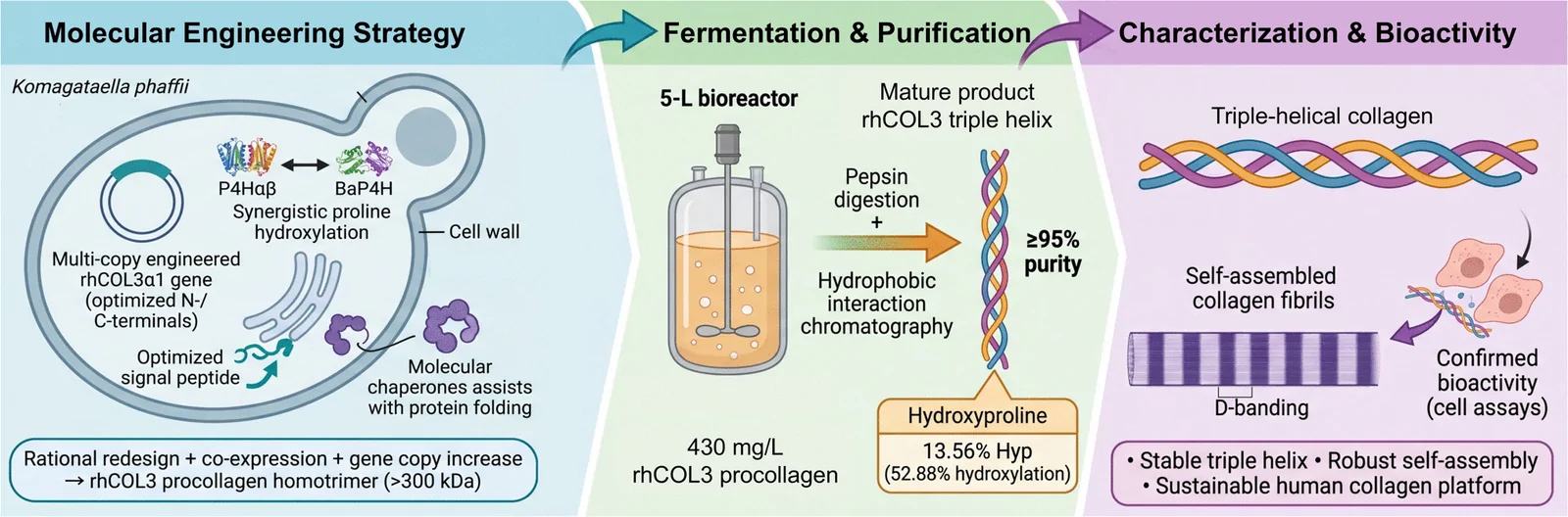

**Supplementary Information:**

The online version contains supplementary material available at https://doi.org/10.1007/s00253-026-13996-7.

## Introduction

Collagen, the most abundant high-molecular-weight protein in the mammalian ECM, serves as a cornerstone component for connective tissues and organs [43, 56]. To date, at least 28 genetically distinct collagen types have been identified in humans, yet all share a conserved fundamental architecture formed by repeating Gly-X-Y amino acid motifs and a distinctive right-handed triple helix, wherein the X and Y positions are typically occupied by proline and hydroxyproline (Hyp), respectively [39]. Among these, type III collagen, a homotrimer composed of three identical α1 polypeptide chains, acts as a critical extracellular structural scaffold that can modulate cell proliferation and migration through interactions with integrins and other ECM components [32, 39, 56]. As a matter of fact, collagen’s structural integrity and biological functions are profoundly dependent on post-translational modifications, particularly the enzymatic proline hydroxylation at the Y position to form 4-Hyp [42]. Intrinsically, the crucial 4-Hyp residue at the Y position plays an indispensable role in stabilizing the triple helix conformation by facilitating the formation of interchain hydrogen bonds of collagen [22, 42]. In the human, 4-Hyp is obtained almost exclusively via the catalytic action of P4H on proline residues within nascent collagen polypeptide chains. Human P4H enzyme exists as an α2β2 tetramer, where the α-subunit provides catalytic activity while the β-subunit acts as a molecular chaperone that stabilizes the soluble and active conformation of the α-subunit [21, 34, 47, 48]. Previous studies have revealed that P4H-catalyzed hydroxylation exhibits strict sequence specificity in collagen [18, 37], occurring almost exclusively at Y-position prolines within the canonical Gly-X-Y motifs, a phenomenon widely regarded as crucial for stabilizing the collagen triple helix.

Considering its unique structural specificity, excellent biocompatibility, and low immunogenicity, collagen has rapidly advanced in diverse biomedical applications in recent years, such as medical repair dressings for wound healing promotion, scaffolds, and tissue implants for regenerative medicine and aesthetic surgery [10, 26, 43, 46]. Currently, commercial collagen is predominantly extracted from animal tissues. However, animal-derived collagen poses inherent risks of immunogenicity and pathogenic transmission, as well as a compositional bias toward type I collagen, making the isolation of type III collagen both costly and inefficient at scale [31, 35]. Consequently, there is an urgent need to develop efficient biomanufacturing methods capable of producing collagen with structural and functional properties comparable to natural human collagen. Over recent decades, advances in synthetic biology, protein engineering, and biomanufacturing have established the heterologous expression of human collagen with defined composition [30, 45, 54]. To the best of our knowledge, numerous studies have reported high-yield expression of recombinant collagen [29, 53], but these “collagen” products are typically truncated collagen peptides or collagen-mimetic proteins with molecular weights below 50 kDa, rather than full-length human collagen possessing the native amino acid sequence and a fully formed triple-helical structure. In addition, several studies explored in various heterologous expression systems, including mammalian cells [51], bacteria [37, 58]*,* yeast [16, 21, 33, 47, 48, 55], and plant platforms [44], have shown that co-expression of P4Hs with collagen effectively promotes the production of hydroxylated or triple-helical collagen. [33] described the expression of recombinant human type I–III collagens in *Komagataella phaffii* (*K. phaffii*, formerly named *Pichia pastoris*) and reported a yield range of approximately 0.2–0.6 g/L. However, that short communication did not disclose critical experimental details such as strain engineering strategies, fermentation conditions, or protein characterization data. Dongwan Biotechnology Inc. (Nanjing, China) achieved successful expression of full-length human type I collagen using CHO cells [51]. Their recombinant collagen displays a high hydroxylation ratio, which enables the formation of a stable triple helix and self-assembly into collagen fibrils, with overall characteristics highly consistent with those of natural human collagen. Nevertheless, its low yield (approximately 300 mg/L) and high production costs severely limit its industrial scalability and in-depth applications. Although several alternative non-human P4H enzymes with similar functions have been identified, including BaP4H from *Bacillus anthracis* (*B. anthracis*) [5, 28, 38, 52, 58], AtP4H from *Arabidopsis thaliana* [17], BmP4H from *Bacillus megaterium* [29], and the virus-encoded enzymes L593 [37, 58] and A085R [13, 41], the recombinant production of full-length, triple-helical collagen in engineered microbial hosts remains challenging, primarily due to two interrelated bottlenecks. On one hand, the insufficient catalytic activity of heterologous P4Hs compromises the efficiency of proline hydroxylation, thereby impairing the proper folding of collagen and its structural integrity. On the other hand, the large molecular weight of full-length collagen, combined with its highly dense Gly-X-Y repeats and widely distributed prolines, poses significant obstacles to rigorous post-translational modification, precise intracellular assembly, and extracellular secretion.

Previous research has established that various strategies, including codon optimization, promoter engineering [12, 14, 23], signal peptide optimization [57], gene dosage amplification [40], and high-cell-density fermentation techniques [6, 11] effectively enhance the expression of exogenous proteins in *K. phaffii*. However, their application to triple-helical collagen production remains unexplored. In this study, we developed an engineered *K. phaffii* platform to efficiently produce full-length triple-helical rhCOL3 procollagen. Our strategy combined rational redesign of N- and C-terminal propeptides, increased gene copy number, synergistic co-expression of human P4Hαβ and *B. anthracis*-BaP4H, and optimization of intracellular expression alongside trimeric folding via signal sequence screening and chaperone co-expression. Subsequent high-cell-density fermentation in 5-L fed-batch bioreactors verified the yield potential and achieved substantial rhCOL3 procollagen production. Mature rhCOL3 was then efficiently purified from fermented yeast cells using a sequential workflow involving cell disruption, pepsin digestion, specific chromatography, and freeze-drying. Moreover, comprehensive biophysical characterization of the purified rhCOL3 was performed employing multiple spectroscopic and morphological techniques, including mass spectrometry (LC-MS/MS), circular dichroism (CD) spectroscopy, differential scanning calorimetry (DSC), Fourier transform infrared spectroscopy (FTIR), and transmission electron microscopy (TEM). Collectively, these analyses confirmed the structural integrity and conformational stability of the triple helix within the obtained rhCOL3. This work presents an effective strategy for producing bioactive human collagen with an authentic triple helix structure, offering promising prospects for its industrial-scale production, functional investigation, and biological applications as alternative collagen-based materials.

## Materials and methods

All reagents and chemicals were purchased from Sangon Biotech (Shanghai) unless stated otherwise. Enzymes and oligonucleotides were purchased from Invitrogen, NEB and GenScript. All primer sequences are provided in Supplementary Materials Tables S1, and all genes of interest were codon-optimized and are provided in Supplementary material Table S2.

### Plasmid construction and *K. phaffii* transformation

Plasmids pPIC9K, pPICZαA, and pGAPZαA were utilized for the expression of P4Hs, recombinant collagen, and molecular chaperones, respectively. Gene synthesis and recombinant plasmid construction were performed commercially by GenScript (Nanjing, China). Codon-optimized genes encoding the human P4H α-subunit (GenBank: KAI4076361.1), β-subunit (GenBank: KAI4052196.1), and *Bacillus anthracis* BaP4H (GenBank: PDZ16530.1) were cloned into pPIC9K under the control of the AOX1 promoter to generate pPIC9K-P4Hα, pPIC9K-P4Hβ, and pPIC9K-BaP4H (Table 1), respectively*.* The β-subunit expression cassette was amplified from pPIC9K-P4Hβ and inserted into the *Aat*II site of pPIC9K-P4Hα to generate pPIC9K-P4Hαβ in tandem. Similarly, the BaP4H expression cassette was PCR-amplified and cloned into the SphI site of pPIC9K-P4Hαβ for tandem expression. The resulting plasmids were linearized with *Bsp*EI and individually electroporated into *K. phaffii* GS115 using a Gene Pulser (Bio-Rad, USA). Transformants were selected on MD agar (13.4 g/L yeast nitrogen base, 20 g/L glucose, and 20 g/L agar) at 29 °C for 3 days and confirmed by sequencing, as previously described [53].

**Table 1:** Plasmids and strains used in this study

Plasmids and strains	Relevant characteristics	Reference or source
Plasmids		
pPIC9K	AOX1 promoter, PBR322 ori, HIS4,Amp and G418 resistance	GenScript
pPICZαA	AOX1 promoter, PBR322 ori, Zeocin resistance	GenScript
pGAPZαA	GAP promoter, PBR322 ori, Zeocin resistance	GenScript
pPIC9K-P4Hα	pPIC9K-derived plasmid, with P4H α-subunit gene inserted between *Bam*HI and *Not*I sites	This work
pPIC9K-P4Hβ	pPIC9K-derived plasmid, with P4H β-subunit gene inserted between *Eco*RI and *Not*I sites	This work
pPIC9K-BaP4H	pPIC9K-derived plasmid, with BaP4H gene inserted between *Bam*HI and *Not*I sites	This work
pPIC9K-P4Hαβ	pPIC9K-P4Hα-derived plasmid, with P4H β-subunit gene expression cassette inserted into *Aat*II site	This work
pPIC9K-P4Hαβ-BaP4H	pPIC9K-P4Hαβ-derived plasmid, with BaP4H gene expression cassette inserted into *Sph*I site	This work
pPICZ-GAPDH	pPICZαA-derived plasmid, with GAPDH gene inserted between *Bst*BI and *Sal*I sites	This work
pPICZ-SigN-rhCOL3α1	pPICZαA-derived plasmid, with rhCOL3α1 gene carrying SigN signal sequence inserted between *Bst*BI and *Sal*I sites	This work
pPICZ-SPc1-rhCOL3α1	pPICZαA-derived plasmid, with rhCOL3α1 gene carrying SPc1 signal sequence inserted between *Bst*BI and *Sal*I sites	This work
pPICZ-αpre-rhCOL3α1	pPICZαA-derived plasmid, with rhCOL3α1 gene carrying αpre signal sequence inserted between *Bst*BI and *Sal*I sites	This work
pPICZ-Ost1-rhCOL3α1	pPICZαA-derived plasmid, with rhCOL3α1 gene carrying Ost1 signal sequence inserted between *Bst*BI and *Sal*I sites	This work
pGAPZ-HACI	pGAPZαA-derived plasmid, with HACI gene inserted between *Bst*BI and *Sal*I sites	This work
pGAPZ-BiP	pGAPZαA-derived plasmid, with BiP gene inserted between *Bst*BI and *Sal*I sites	This work
pGAPZ-FKBP22	pGAPZαA-derived plasmid, with FKBP22 gene inserted between *Bst*BI and *Sal*I sites	This work
pGAPZ-Hsp47	pGAPZαA-derived plasmid, with Hsp47 gene inserted between *Bst*BI and *Sal*I sites	This work
pGAPZ-EROI	pGAPZαA-derived plasmid, with EROI gene inserted between *Bst*BI and *Sal*I sites	This work
pGAPZ-Bcy1	pGAPZαA-derived plasmid, with Bcy1 gene inserted between *Bst*BI and *Sal*I sites	This work
CRISPR-Hygr-Chr1-1	A gene-editing plasmid, containing GAP promoter used to express sgRNA, and bidirectional promoter PHTX1 used to express the Cas9 gene and PapRecT-PaSSB cistron coding gene, as well as PBR322 ori and Hyg resistance	Lab stock
CRISPR-Hygr-△*ku70*	CRISPR-Hygr-Chr1-1-derived plasmid, containing a unique N20 sequence targeting the ku70 region for ku70 gene knockout	This work
CRISPR-Hygr-Chr1-4	CRISPR-Hygr-Chr1-1-derived plasmid, containing a unique N20 sequence targeting the intergenic region between the PAS_chr1-4_0248 and _0249 genes for gene knockin	This work
Strains		
*E. coli* DH5α	F-φ80 lacZΔM15Δ(lacZYA-argF) U169 recA1endA1 hsdR17(rk-,mk+) supE44λ-thi-1 gyrA96 relA1phoA	Sangon Biotech
*K. phaffii* GS115	Mut+, his4-, AOX1, AOX2	Lab stock
GS-P4Hαβ	GS115 derivative strain, his4-:: P_AOX1_- αMF-P4Hβ-T_AOX1_- P_AOX1_-P4Hα-T_AOX1_,HIS4 and G418 resistance	This work
GS-BaP4H	GS115 derivative strain, his4-:: P_AOX1_- BaP4H -T_AOX1_, HIS4 and G418 resistance	This work
GS-P4Hαβ-BaP4H	GS115 derivative strain, his4-:: P_AOX1_- αMF-P4Hβ-T_AOX1_- P_AOX1_-P4Hα-T_AOX1_- P_AOX1_- BaP4H -T_AOX1_, HIS4 and G418 resistance	This work
GS-SigN-rhCOL3α1	GS115 derivative strain,, AOX1:: P_AOX1_-SigN-rhCOL3α1-T_AOX1_, HIS4, G418 and Zeocin resistance	
GS-BaP4H-SigN-rhCOL3α1	GS-BaP4H derivative strain, AOX1:: P_AOX1_-SigN-rhCOL3α1-T_AOX1_, HIS4, G418 and Zeocin resistance	This work
GS-P4Hαβ-SigN-rhCOL3α1	GS-P4Hαβ derivative strain, AOX1:: P_AOX1_-SigN-rhCOL3α1-T_AOX1_, HIS4, G418 and Zeocin resistance	This work
GS-P4Hαβ-BaP4H-SigN-rhCOL3α1	GS-P4Hαβ-BaP4H derivative strain, AOX1:: P_AOX1_-SigN-rhCOL3α1-T_AOX1_, HIS4, G418 and Zeocin resistance	This work
GS-P4Hαβ-BaP4H-SPc1-rhCOL3α1	GS-P4Hαβ-BaP4H derivative strain, AOX1:: P_AOX1_-SPc1-rhCOL3α1-T_AOX1_, HIS4, G418 and Zeocin resistance	This work
GS-P4Hαβ-BaP4H-αpre-rhCOL3α1	GS-P4Hαβ-BaP4H derivative strain, AOX1:: P_AOX1_-αpre-rhCOL3α1-T_AOX1_, HIS4, G418 and Zeocin resistance	This work
GS-P4Hαβ-BaP4H-Ost1-rhCOL3α1	GS-P4Hαβ-BaP4H derivative strain, AOX1:: P_AOX1_-Ost1-rhCOL3α1-T_AOX1_, HIS4, G418 and Zeocin resistance	This work
GS△ku70-P4Hαβ-BaP4H-Ost1-rhCOL3α1	GS-P4Hαβ-BaP4H-Ost1-rhCOL3α1 derivative strain with inactivated ku70 gene	This work
GS△ku70-P4Hαβ-BaP4H-Ost1-rhCOL3α1-HACI	GS115△ku70-P4Hαβ-BaP4H-Ost1-rhCOL3α1derivative strain, PAS_chr1-4_0248-0249::P_GAP_-HAC1-T_AOX1_	This work
GS△ku70-P4Hαβ-BaP4H-Ost1-rhCOL3α1-BiP	GS115△ku70-P4Hαβ-BaP4H-Ost1-rhCOL3α1derivative strain, PAS_chr1-4_0248-0249::P_GAP_-BiP-T_AOX1_	This work
GS△ku70-P4Hαβ-BaP4H-Ost1-rhCOL3α1-FKBP22	GS115△ku70-P4Hαβ-BaP4H-Ost1-rhCOL3α1derivative strain, PAS_chr1-4_0248-0249::P_GAP_-FKBP22-T_AOX1_	This work
GS△ku70-P4Hαβ-BaP4H-Ost1-rhCOL3α1-Hsp47	GS115△ku70-P4Hαβ-BaP4H-Ost1-rhCOL3α1derivative strain, PAS_chr1-4_0248-0249::P_GAP_-Hsp47-T_AOX1_	This work
GS△ku70-P4Hαβ-BaP4H-Ost1-rhCOL3α1-EROI	GS115△ku70-P4Hαβ-BaP4H-Ost1-rhCOL3α1derivative strain, PAS_chr1-4_0248-0249::P_GAP_-EROI-T_AOX1_	This work
GS△ku70-P4Hαβ-BaP4H-Ost1-rhCOL3α1-Bcy1	GS115△ku70-P4Hαβ-BaP4H-Ost1-rhCOL3α1derivative strain, PAS_chr1-4_0248-0249::P_GAP_-Bcy1-T_AOX1_	This work

**Fig. 1:**
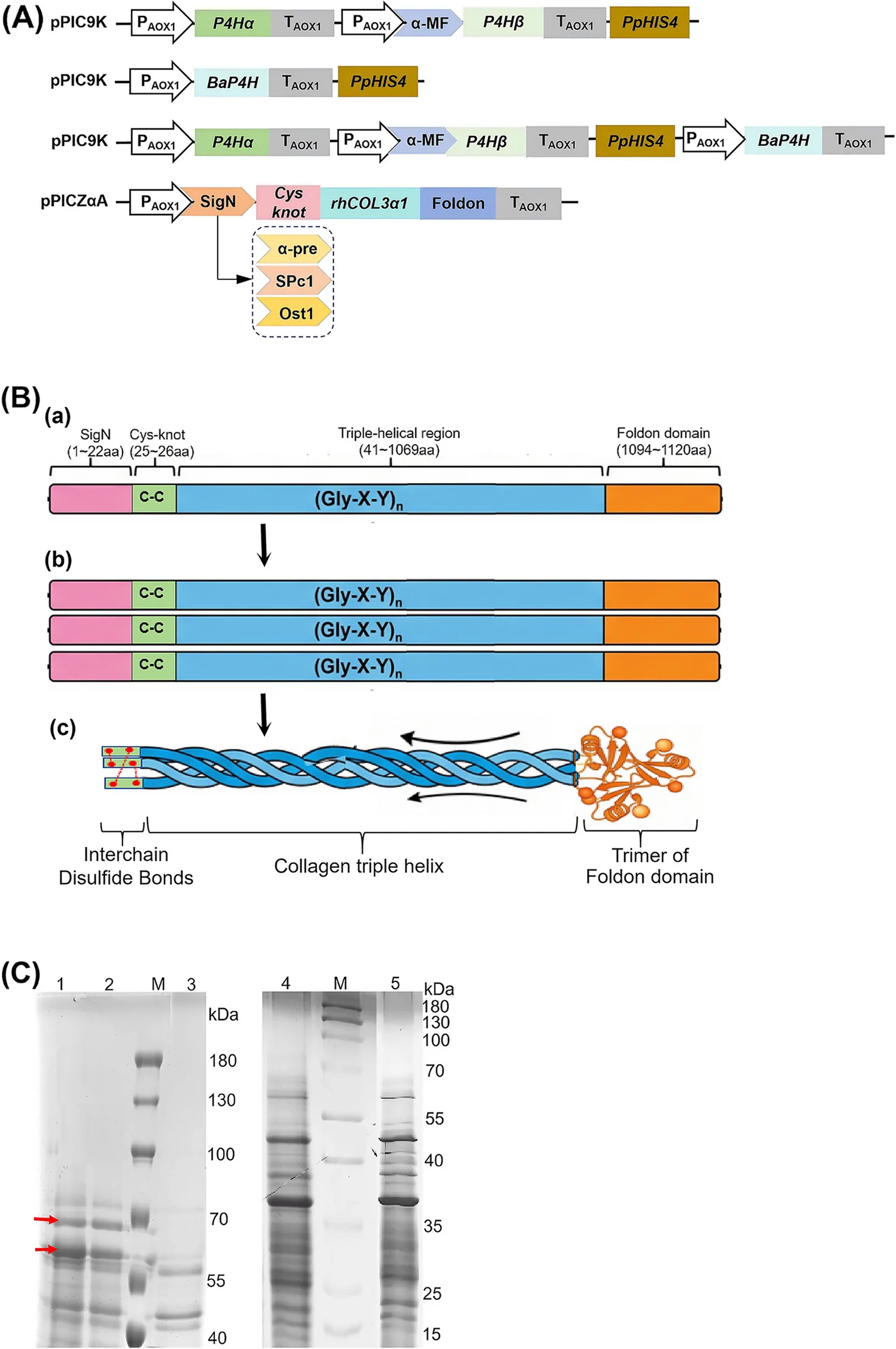

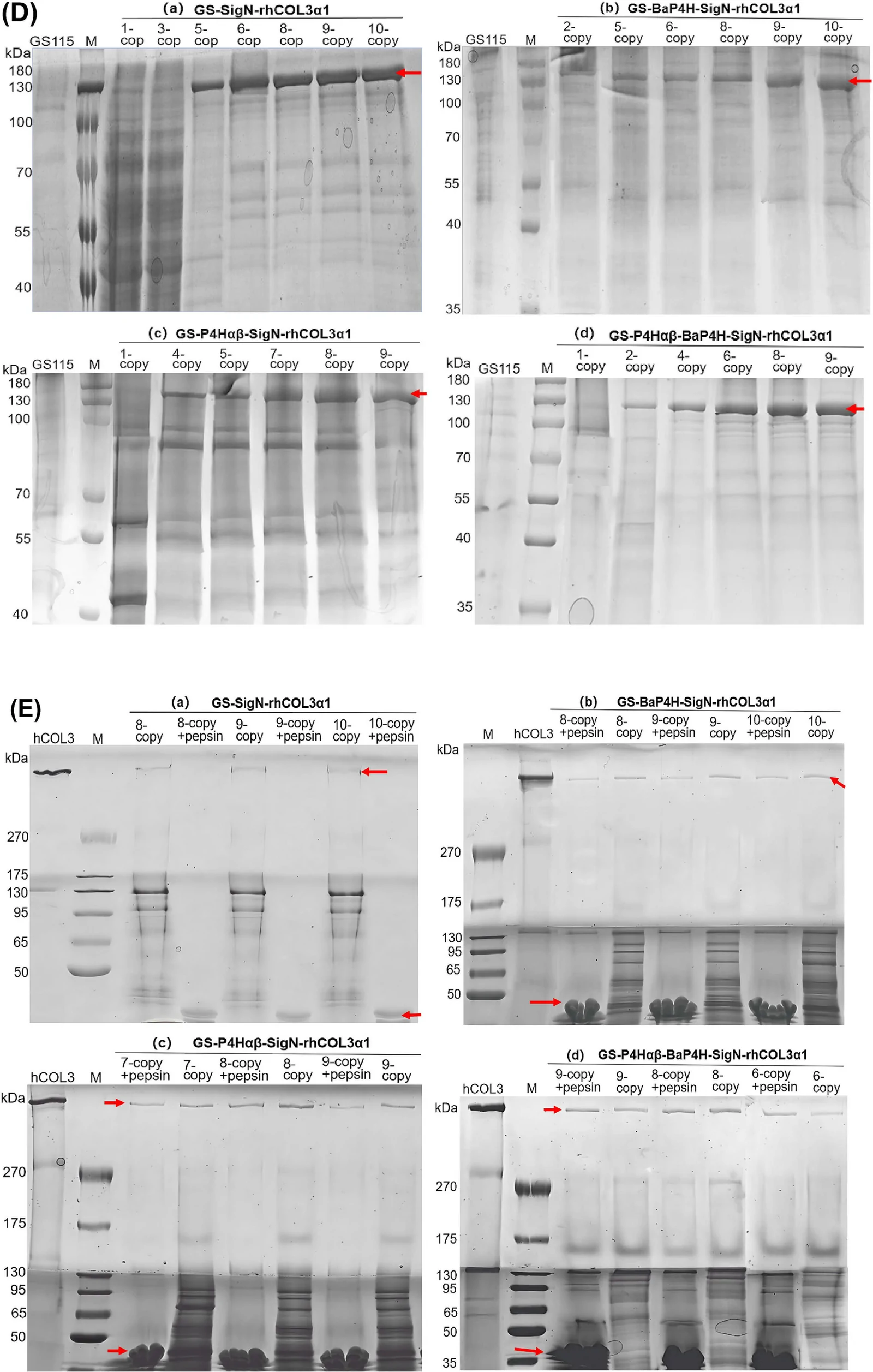

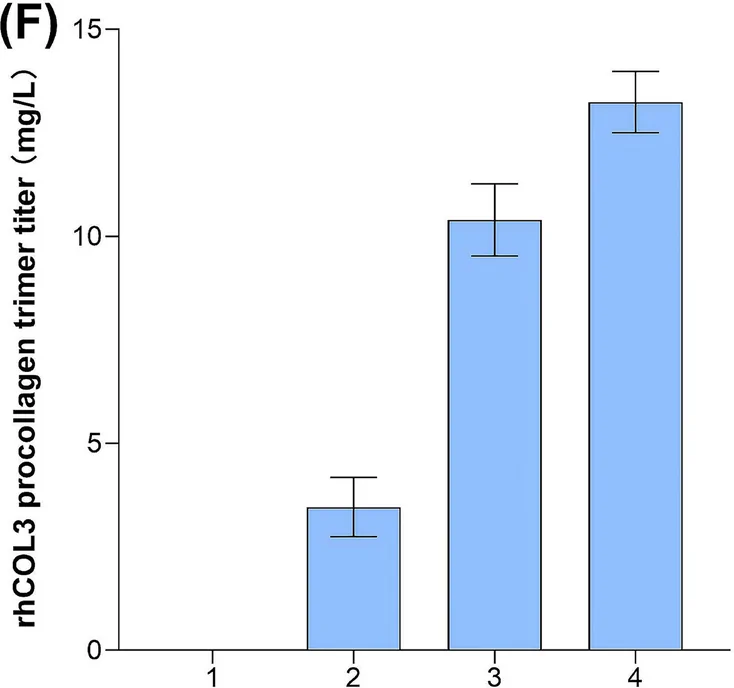
Intracellular expression of recombinant P4Hs and rhCOL3 procollagen in *K. phaffii*. **A** Schematic diagram of plasmid construction for co-expression of P4Hs and rhCOL3 procollagen. Plasmid pPIC9K was used to express P4Hs, including the human P4H α-subunit, β-subunit, and the bacterial BaP4H. Plasmid pPICZαA was used to express recombinant human type III collagen (rhCOL3) α1 chains containing different signal sequences (sigN, SPc1, αpre, and Ost1). Cys-knot stands for the motif of Gly-Pro-Cys-Cys. Foldon represents the 27-residue trimerization domain of bacteriophage T4 fibritin. **B** Schematic diagram of the triple helix assembly of rhCOL3. (a) Structure of the rhCOL3 α1 single chain; (b) co-localization of three chains; (c) trimerization of the Foldon domain drives assembly. **C** Reducing SDS-PAGE analysis of recombinant P4Hs in neutral cell lysates after 48 h of shake-flask fermentation using a stacking gel containing 5% (w/v) acrylamide and a separating gel containing 8% (w/v) acrylamide. Lane M: Marker; lane 1: GS-P4Hαβ strain; lane 2: GS-P4Hαβ-BaP4H strain; lane 3 and 5: *K. phaffii* GS115 strain (control); lane 4: GS-BaP4H strain. Arrows indicate the P4H α-subunit (~ 60 kDa) and β-subunit (~ 55 kDa); the BaP4H band (~ 27 kDa) was not detected. **D** Reducing SDS-PAGE analysis of recombinant collagen in acidified cell lysates after 48 h of shake-flask fermentation using a stacking gel containing 5% (w/v) acrylamide and a separating gel containing 8% (w/v) acrylamide. (a) GS-sigN-rhCOL3α1 strain (with 1 ~ 10 copies of the rhCOL3α1 gene); (b) GS-BaP4H-sigN-rhCOL3α1 strain (with 2 ~ 10 copies of the rhCOL3α1 gene); (c) GS-P4Hαβ-sigN-rhCOL3α1 strain (with 1 ~ 9 copies of the rhCOL3α1 gene); (d) GS-P4Hαβ-BaP4H-sigN-rhCOL3α1 strain (with 1 ~ 9 copies of the rhCOL3α1 gene). Lane M: Marker; lane GS115: *K. phaffii* GS115 (control). Arrows indicate the rhCOL3α1 chain (~ 130 kDa. **E** Non-reducing SDS-PAGE analysis of recombinant procollagen in acidified cell lysates treated and untreated with pepsin using a stacking gel containing 5% (w/v) acrylamide and a discontinuous separating gel. Samples were taken from three high-copy strains of each recombinant type. (a) GS-sigN-rhCOL3α1 strain (with 8 ~ 10 copies of the rhCOL3α1 gene); (b) GS-BaP4H-sigN-rhCOL3α1 strain (with 8 ~ 10 copies of the rhCOL3α1 gene); (c) GS-P4Hαβ-sigN-rhCOL3α1 strain (with 7 ~ 9 copies of the rhCOL3α1 gene); (d) GS-P4Hαβ-BaP4H-sigN-rhCOL3α1 strain (with 6 ~ 9 copies of the rhCOL3α1 gene). Lane M: Marker; lane hCOL3: natural human type III collagen standard (2 μg loaded, control). Arrows indicate the procollagen trimer band (> 300 kDa), and the pepsin band is visible at ~ 40 kDa. Two-tone appearance at ~ 130 kDa: The distinct visual transition is not an artifact, and since the target procollagen trimer exceeds 300 kDa, we employed a non-reducing discontinuous SDS-PAGE system with a 5% (w/v) acrylamide stacking gel and a two-layer separating gel composed of a 6% (w/v) upper layer and a 12% (w/v) lower layer. This design improves resolution of high-molecular-weight species and causes the transition at ~ 130 kDa. **F** Quantitative analysis of rhCOL3 procollagen trimer titers in high-copy-number strains. The densitometric values of the desired bands in the destained gels were measured using ImageJ software, and normalized against that of natural hCOL3 standard for relative quantification of proteins. Only the highest titer and copy number of each recombinant strain are shown. Column 1: GS-sigN-rhCOL3α1 strain (a control with only trace amounts of procollagen trimer observed in its high-copy strains); column 2: GS-BaP4H-sigN-rhCOL3α1 strain (with 10 copies of the rhCOL3α1 gene); column 3: GS-P4Hαβ-sigN-rhCOL3α1 strain (with 8 copies of the rhCOL3α1 gene); column 4: GS-P4Hαβ-BaP4H-sigN-rhCOL3α1 strain (with 8 copies of the rhCOL3α1 gene). All assays were repeated at least three times and results were expressed as the mean ± SD

An engineered rhCOL3α1 chain was rationally designed to facilitate collagen triple helix formation (Fig. 1A and B). The construct comprises a core functional region corresponding to residues 154–1220 of the natural hCOL3 α1 chain (GenBank: KAI4037252.1) but lacks its native propeptides. An N-terminal Cys-knot motif (Gly-Pro-Cys-Cys) was incorporated to enable potential interchain covalent cross-linking, and a 27-residue Foldon domain of bacteriophage T4 fibritin was fused to the C-terminus to serve as a trimerization nucleation domain [4, 9]. Codon-optimized fusion DNA fragment (Table S2) encoding the engineered rhCOL3α1 chain was cloned into the *Bst*BI–*Sal*I sites of the pPICZαA to generate pPICZ-sigN-rhCOL3α1. The verified plasmid was linearized with *Sac*I and electroporated separately into the recombinant strains expressing distinct P4Hs. Transformants were selected on YPD agar plates containing 100 μg/mL Zeocin. Single colonies were subsequently streaked onto YPD agar plates supplemented with 0.5, 1.0, or 2.0 mg/mL Zeocin, and incubated at 29 °C to enrich for high-copy-number integrants. Positive clones were confirmed by DNA sequencing.

### Expression of rhCOL3 procollagen in *K. phaffii*

For shake-flask fermentation, single colonies were inoculated into 5 mL of YPD medium, and cultured overnight at 29 °C and 250 rpm to prepare primary seed liquid. Subsequently, 2% (v/v) of the seed culture was transferred into a 250-mL shake flask containing 50 mL of BMGY medium (10 g/L glycerin, 20 g/L peptone, 10 g/L yeast extract, 100 mM potassium phosphate (pH 6.0), 13.4 g/L YNB, 4 × 10^−4^ g/L biotin) and incubated at 29 °C and 250 rpm until the OD600 reached 5~6. The cells were centrifuged at room temperature at 5000 × g for 2 min, washed with sterile deionized water to remove residual glycerol, and then resuspended in 50 mL of fresh BMMY medium (5.0 mL/L methanol, 20 g/L peptone, 10 g/L yeast extract, 100 mM potassium phosphate (pH 6.0), 13.4 g/L YNB, 4 × 10^−4^ g/L biotin). Cultures were maintained at 25 °C and 250 rpm. Every 24 h, methanol was replenished to a final concentration of 5.0 mL/L, together with sodium L-ascorbate (120 µg/mL) and FeSO_4_ (100 µg/mL), over a 48-h induction period.

For fed-batch fermentation, the enlarged cultivation was conducted in a 5-L bioreactor (BXBIO, Shanghai, China) using the following procedure. The engineered strain was first activated and pre-cultured in BMGY medium until the OD600 reached ~ 6.0, and then inoculated into 3 L of basal salt medium containing 27 mL/L H_3_PO_4_, 0.93 g/L CaSO_4_·2H_2_O, 18 g/L K_2_SO_4_, 14.9 g/L MgSO_4_·7H_2_O, 4.13 g/L KOH, 40 g/L glycerol, and 4.5 mL/L PTM1 trace salt solution (6 g/L CuSO_4_·5H_2_O, 0.08 g/L KI, 3 g/L MnSO_4_·H_2_O, 0.02 g/L H_3_BO_3_, 0.2 g/L Na_2_MoO_4_·2H_2_O, 0.5 g/L CoCl_2_, 20 g/L ZnCl_2_, 6.5 g/L FeSO_4_·7H_2_O, 0.2 g/L D-biotin, and 5.0 mL/L H_2_SO_4_). Cultivation conditions were initiated at 29 °C, pH 6.0, 300 rpm, and an aeration rate of 1 vvm. During the glycerol batch phase, dissolved oxygen (DO) gradually declined to a minimum and subsequently rebounded above 60%, indicating complete depletion of the initial glycerol in the BSM medium. In the subsequent glycerol fed-batch phase, agitation speed was increased to 800 rpm, and a feeding solution of 50% (w/v) glycerol supplemented with 12 mL/L PTM1 was supplied at a constant rate of 18 mL/(L·h) until the wet cell weight reached ~ 400 g/L. Upon glycerol exhaustion (DO > 60%), the methanol induction phase was initiated. The aeration rate was increased to 1.5 vvm, and the temperature and pH were shifted to 25 °C and 5.0, respectively. Methanol containing 12 mL/L PTM1 was fed to initiate protein expression. During the initial 4 h of induction, methanol was pulsed intermittently (3.2 g/(L·h)) to allow metabolic adaptation. Thereafter, the feeding rate was incrementally increased using a pulse-feeding strategy whenever DO exceeded 50% to maintain DO at 30–40%. In addition, L-ascorbic acid sodium salt (150 µg/mL) and FeSO_4_ (100 µg/mL) were supplemented every 24 h during the methanol induction phase. The fermentation was terminated after 60 h of induction.

### Optimization of signal sequences for rhCOL3 procollagen expression

In this study, three alternative signal sequences (Table 2), SPc1 (derived from natural human type I collagen), αpre, and Ost1 (both from *S. cerevisiae*), were individually linked to rhCOL3α1 chain, and cloned into the *Bst*BI-*Sal*I sites of pPICZαA (Fig. 1A). Each construct was linearized with *Sac*I, followed by electroporation into the recombinant strain capable of synergistically expressing P4Hαβ and BaP4H. High-copy-number positive transformants were screened and verified by colony PCR and DNA sequencing.

**Table 2:** Signal sequences used in this study

Signal sequences	Amino acid sequences (N → C)	Optimized nucleotide sequences (5′ → 3′)
SigN	MSFVQKGSWLLLALLHPTIILA	ATGTCATTTGTGCAGAAAGGTAGCTGGTTACTCCTAGCACTTTTGCATCCCACGATCATTCTGGCT
αpre	MRFPSIFTAVLFAASSALA	ATGAGATTTCCTTCAATTTTTACTGCTGTTTTATTCGCAGCATCCTCCGCATTAGCT
SPc1	MFSFVDLRLLLLLAATALLTHG	ATGTTCTCCTTCGTGGACCTTCGTTTGCTGCTACTGTTGGCAGCTACAGCCCTTTTGACTCATGGA
Ost1	MRQVWFSWIVGLFLCFFNVSSA	ATGAGACAGGTTTGGTTCTCCTGGATCGTCGGTTTGTTCCTGTGCTTCTTCAACGTTTCTTCCGCC

### Coexpression of molecular chaperones via genome editing

Six chaperone genes, *HAC1* (GenBank: XP_002490039.1), *Bip* (GenBank: XP_002491027.1), *FKBP22* (GenBank: NP_060416.1), *Hsp47* (GenBank: NP_001193943.1), *ERO1* (GenBank: XP_002489645.1), and *Bcy1* (GenBank: KZV10583.1), were commercially synthesized and individually inserted into the *Eco*RI*-Sal*I sites of the GAP-promoter-driven vector pGAPZαA, generating six expression plasmids for these chaperones (Table 1).

CRISPR/Cas9-mediated genome editing was employed for targeted integration. To enhance homologous recombination efficiency in *K. phaffii*, the *ku70* gene was first knocked out as described previously [15]. The CRISPR/Cas9-based editing plasmid CRISPR-Hygr-Chr1-1, which expresses sgRNA from the GAP promoter and Cas9 from the bidirectional PHTX1 promoter, was kindly donated by Professor Liu’s team at Jiangnan University for precision genome editing in *K. phaffii* [7, 8]. Using this plasmid as a backbone, plasmids CRISPR-Hygr-∆ku70 and CRISPR-Hygr-Chr1_4 were constructed via PCR amplification using the primer pairs sgRNA-∆ku70-F/R and sgRNA-Chr1_4-F/R (Table S1), respectively, following the methodology described by Deng et al*.* [8]. For the seamless deletion of *ku70*, the upstream and downstream homologous arms (~ 1000 bp each) were amplified from the *K. phaffii* GS115 genome using primer pairs ku70-UP-F/R and ku70-DO-F/R, respectively, and fused into a single donor DNA construct via overlap extension PCR. Similarly, for integration of chaperone expression cassettes into the neutral genomic locus between *PAS_chr1-4_0248* and *PAS_chr1-4_0249* (Table S3), the upstream and downstream homology arms of this locus and each chaperone expression cassette were amplified using the corresponding primer pairs listed in Table S1. These three PCR products were assembled using overlap extension PCR to generate donor constructs for site-specific integration of each chaperone cassette. For both genomic deletion and integration, CRISPR plasmids and donor constructs (2 µg each) were co-transformed into competent cells via electroporation using an optimized electroporation protocol [8]. Transformants were selected on YPD agar plates supplemented with 250 μg/mL hygromycin. Positive colonies were verified by PCR and Sanger sequencing using primer pairs ku70-UP-F/ku70-DO-R (for deletion) and 0248-UP-F/0249-DO-R (for integration), respectively. CRISPR plasmids were subsequently cured by culturing in non-selective YPD medium.

### Quantitative PCR analysis for rhCOL3α1 copy numbers

Genomic DNA was extracted from *K. phaffii* using a Yeast Genomic DNA Extraction Kit (Solarbio, Beijing, China), with glyceraldehyde-3-phosphate dehydrogenase (GAPDH) selected as the reference gene. The GAPDH gene was amplified using the primer pair GAPDH-F/R and cloned into the *Eco*RI-*Sal*I sites of pPICZαA to generate pPICZ-GAPDH. Standard curves were generated using serial dilutions of both pPICZ-GAPDH and the rhCOL3α1 expression plasmid. The copy number of the integrated rhCOL3α1 gene in the yeast genome was subsequently quantified following the method described by Abad et al*.* [1].

### SDS-PAGE analysis and protein quantification

Yeast cells from methanol-induced cultures were harvested by centrifugation (5000 × g, 2 min, 4 °C). The pellets were washed with ice-cold sterile water and resuspended in pre-chilled lysis buffer (10% glycerol, 1 mM PMSF, 1 mM EDTA, 0.2 M NaCl, 50 mM sodium phosphate buffer, and pH 6.0). Cell disruption was performed using an AH-NANO high-pressure homogenizer (ATS Engineer, Suzhou, China) at 1200 ~ 1300 bar for three cycles. The resulting lysate was acidified to pH 3.5 with acetic acid when necessary, then centrifuged (5000 × g, 5 min, 4 °C); the supernatant was subsequently collected for analysis. For reducing SDS-PAGE analysis, samples were mixed with a loading buffer containing 100 mM dithiothreitol (DTT), and denatured at 100 °C for 10 min, followed by electrophoresis on 8% acrylamide gels. For non-reducing SDS-PAGE analysis, samples were mixed with a DTT-free loading buffer and not subjected to heat treatment, followed by electrophoresis on a discontinuous two-layer separating gel, which consists of a 6% (w/v) acrylamide upper layer and a 12% (w/v) acrylamide lower layer, alongside a 270-kDa prestained protein ladder (Beyotime Biotechnology, Shanghai) as the molecular weight standard. All gels were stained with Coomassie Brilliant Blue for visualization. However, in crude cell lysates, the high abundance of interfering host proteins precludes accurate quantification of recombinant collagen using standard colorimetric assays, such as the BCA or Bradford method. To enable a cost-effective, efficient, and rapid preliminary comparison across the various engineered strains, we therefore employed a semi-quantitative approach based on band intensity. The densitometric values of the desired bands in the destained gels were measured using ImageJ software, and normalized against that of natural hCOL3 standard (Product Number: CC054, Sigma-Aldrich, USA) for relative protein quantification [2, 53].

### Purification of triple-helical rhCOL3

Triple-helical rhCOL3 was purified and prepared following the procedure below. Yeast cell lysates were acidified and digested with pepsin (Sunson, Hebei, China) at a final concentration of 1.0 mg/mL for 12 h at 4 °C. Following centrifugation at 5000 × g for 10 min to remove debris, the supernatant fractions were concentrated using a Microcon® centrifugal filter (MWCO 100 kDa, Millipore). The concentrate was then subjected to hydrophobic interaction chromatography on a Phenyl-Sepharose FF column (TRUKING, Changsha, China). Unbound proteins and pigments were removed by washing with equilibration buffer, and rhCOL3 was eluted with a solution pre-mixed with 0.5 M NaCl and 1.0 M acetic acid. The eluted fractions containing rhCOL3 were pooled and dialyzed extensively against 0.2 M acetic acid. The purified rhCOL3 was lyophilized using an FD-1E-50 vacuum freeze dryer (BIOCOOL, Beijing, China), and stored at 4 °C.

### SEC–HPLC analysis

Protein purity was analyzed using a Waters Alliance e2695 HPLC system equipped with a 2489 UV/Visible Detector. The samples were separated on a SHIMSEN Ankylo SEC-300 column (4.6 × 300 mm, 5 µm) purchased from Shimadzu Corporation (Kyoto, Japan). The mobile phase consisted of 300 mM sodium phosphate buffer (pH 6.0) supplemented with 150 mM NaCl, and the flow rate was 0.4 mL/min. Samples (10 µL) were injected and separated at 25 °C with detection at 221 nm.

### Protein characterization

#### Mass spectrometry analysis

The gel strips containing the target bands cut from the non-reducing SDS-PAGE gel, or the purified protein samples, were treated with 10 mM DTT and 50 mM iodoacetamide (IAA) for reductive alkylation. It was then digested by trypsin, chymotrypsin, and *Staphylococcus* Glu-C protease to generate a range of degraded peptides with varying molecular weights, which were further analyzed by LC-MS/MS using an Easy-nLC 1200 system coupled to an Orbitrap Fusion Lumos Tribrid mass spectrometer (ThermoFisher Scientific, USA). The raw data were processed and retrieved for analysis using the PEAKS Studio 13 software (Bioinformatics Solutions Inc., Waterloo, Canada).

#### Amino acid composition and hydroxyproline content

To clarify the amino acid profile of rhCOL3, the lyophilized sample was neutralized with NaOH after being hydrolyzed with 6 M HCl at 120 °C for 20 h, and derivatized with O-phthalaldehyde (OPA) and fluorenylmethyl chloroformate (FMOC-Cl). Each amino acid content was determined using an automatic amino acid analyzer (Hitachi, Tokyo, Japan). The concentration of each amino acid was calculated based on the area of the corresponding chromatographic peak. The percentage of proline hydroxylation was calculated according to the following formula (1):

1 $$Hyp\,(\%)=\frac{CHyp}{CHyp+CPro\times\frac{Mr(Hyp)}{Mr(Pro)}}\times100$$

where *C*_*Hyp*_ and *C*_*pro*_ represent the mass concentrations of Hyp and proline detected in the sample, respectively, and Mr_(Hyp)_ and Mr_(Pro)_ denote their molecular weights (g/mol).

#### CD spectroscopy analysis

The triple helix conformation of collagen was characterized by CD spectroscopy using a J-1500 spectropolarimeter (JASCO, Japan). Lyophilized rhCOL3 was dissolved in 0.5 M acetic acid. Far-UV spectra (190–260 nm) were recorded in a 1-mm path-length quartz cuvette at a scan speed of 50 nm/min with a data interval of 0.1 nm. Baseline correction was performed using 0.5 M acetic acid. Characteristic peaks at ~220 nm (positive) and ~195 nm (negative) were used as indicators of the triple helix formation.

#### FTIR spectroscopy analysis

The spectrum of purified rhCOL3 was detected using a NEXUS FTIR spectrometer (Thermo Nicolet, Tempe, AZ, USA) in attenuated total reflection mode. The sample was mixed and milled with pure KBr at a ratio of 1:100, then placed into the sample slot for tableting, and scanned in the infrared region using the FTIR spectrometer. An air spectrum was employed for background correction, and all spectra were collected over a wavelength range of 400–4000 cm^−1^, as reported previously [51].

#### Thermal stability analysis

The melting temperature (*Tm*), used to quantitatively characterize the thermal stability of collagen [51], was determined by DSC using a MicroCal PEAQ-DSC (Malvern Panalytical, USA). Briefly, a linear temperature program ranging from 20 to 60 °C at a heating rate of 1 °C/min was employed, with ice water used as the cooling medium. A blank solution of 5 mM acetic acid was used as the reference. The total denaturation enthalpy (ΔH) was determined by measuring the area of the DSC thermogram, and the *Tm* of purified rhCOL3 was assigned to the maximum of the endothermic peak.

#### Turbidity analysis and fibrillogenesis

Lyophilized sample was dissolved in 5 mM acetic acid at a concentration of 6 mg/mL. Two volumes of 30 mM PBS buffer (3 mM KH_2_PO_4_, 27 mM Na_2_HPO_4_, 30 mM KCl, 175 mM NaCl, and pH 7.6) were added to achieve a final collagen concentration of 2.0 mg/mL for self-assembly. Turbidity was monitored at 313 nm and 30 °C for 3500 s using an advanced multimode microplate reader (Spark, TECAN, Switzerland). For observation of collagen morphology by TEM, a 200-mesh copper grid was incubated on a 10 μL droplet of the collagen gel suspension, and incubated for 10 min to facilitate protein adsorption. Excess liquid was carefully removed with filter paper, and the grid was negatively stained with 1% uranyl acetate for 1 min. After removing the excess stain, grids were air-dried under ambient conditions for at least 1 h. Images were captured using a JEM-1400 microscope (Hitachi, Japan) at an accelerating voltage of 80 kV.

### Cell experiments

#### Cell proliferation

The effect of triple-helical rhCOL3 on cell proliferation was evaluated using human skin fibroblast (HSF) cells cultured in a 5% CO_2_ cell incubator at 37 °C. 100 μL of cell suspension (8 × 10^3 cells per well) was seeded into 96-well plates and incubated for 24 h. The culture medium was subsequently removed, and the cells were washed with PBS (pH 7.4, Gibco), followed by additions of triple-helical rhCOL3 at concentrations of 0.01, 0.02, 0.05, 0.1, 0.25, 0.5, and 1.0 mg/mL, respectively. Control wells received an equal volume of DMEM alone. After an incubation of 24 h, cell viability was determined using the Cell Counting Kit-8 (CCK-8), and the absorbance was measured at a wavelength of 450 nm. The cell proliferation rate was calculated using the following formula (2):

2 $$\mathrm{Cell}\;\mathrm{proliferation}\;\mathrm{rate}\;(\%)=OD{450}_{\left(rhCOL3\right)}/OD{450}_{\left(control\right)}\times100$$

#### Cell adhesion

A CCK-8 assay was used to assess the adhesion strength of collagen as described previously [51]. One hundred microliters of 0.1 mg/mL triple-helical rhCOL3 and PBS was separately coated on the surface of a 96-well plate and incubated at 37 °C for 4 h. After removing the excess solution, 100 µL of BSA was added and incubated at 37 °C for 1 h. Wash the wells with PBS, and HaCaT cell suspension (1 × 10^5^ cells per well) was seeded and incubated at 37 °C for 6 h. Non-adherent cells were removed by PBS washing. The absorbance at 450 nm was recorded and compared. The adhesion rate was calculated using the following formula (3):

3 $$\mathrm{Cell}\;\mathrm{adhesion}\;\mathrm{rate}\;\left(\%\right)=\left(\mathrm{number}\;\mathrm{of}\;\mathrm{adherent}\;\mathrm{cells}/\mathrm{number}\;\mathrm{of}\;\mathrm{initially}\;\mathrm{seeded}\;\mathrm{cells}\right)\times100$$

#### Cell migration

Migration was analyzed by a scratch assay. HaCaT cells (2 × 10^5^ cells per well) were seeded in 6-well plates and cultured to 80–90% confluence. The supernatant was removed, and a linear scratch was drawn horizontally across each well using a sterile pipette tip. The detached cells were washed away with PBS. Cells were then treated with 2 mL of 0.1 mg/mL rhCOL3, while control wells received an equal volume of DMEM. Images were captured at 0, 24, and 48 h using a microscope. The area of each scratch region was analyzed using the ImageJ software, and the migration ratio was calculated using the following formula (4):

4 $$\mathrm{Cell}\;\mathrm{migration}\;\mathrm{ratio}\left(\%\right)=\left(\mathrm{initial}\;\mathrm{scratch}\;\mathrm{area}\;-\;\mathrm{scratch}\;\mathrm{area}\;\mathrm{at}\;\mathrm a\;\mathrm{specific}\;\mathrm{time}\;\mathrm{point}\right)/\mathrm{initial}\;\mathrm{scratch}\;\mathrm{area}\;\times\;100$$

## Results

### Synergistic effect of P4Hs on rhCOL3 procollagen expression

Prolyl‐4‐hydroxylase catalyzes the site-specific hydroxylation of proline residues to 4-Hyp, a modification essential for the proper folding and thermal stability of collagen triple helix [36]. Although P4H activity is indispensable for recombinant human collagen production, native P4Hs often suffer from low expression and poor stability in heterologous hosts. To achieve efficient proline hydroxylation and subsequent triple helix formation, we comparatively evaluated the catalytic performance of two evolutionarily distinct P4Hs with previously reported high activity [18, 26, 34, 37, 58] He et al*.* 2015; [33] Schnicker et al*.* 2016: human P4Hαβ and *Bacillus* anthracis-derived BaP4H. Codon-optimized genes for *K. phaffii* were cloned into the pPIC9K vector (Fig. 1A), and the resulting plasmids, pPIC9K-P4Hαβ, pPIC9K-BaP4H, and pPIC9K-P4Hαβ-BaP4H, were linearized and electroporated into *K. phaffii* GS115 to generate stable p4H-expressing strains, GS-P4Hαβ, GS-BaP4H, and GS-P4Hαβ-BaP4H (Table 1)*.* Following 48 h of methanol-induced cultivation in shake-flasks, SDS-PAGE analysis revealed distinct bands for the P4H *α*-subunit (~ 60 kDa) and *β*-subunit (~ 55 kDa) (Fig. 1C). In contrast, no obvious band corresponding to BaP4H (~ 27 kDa) was observed, which is consistent with its previously reported low expression level in *K. phaffii* [52]. The BaP4H band was likely obscured by abundant endogenous host proteins released during cell lysis, which create a dense background on the gel. The α-subunit band was more intense under standardized loading conditions (equal cell concentration, lysis, and loading volume), suggesting its higher expression level and providing a molecular basis for its hydroxylation capacity when co-expressed with collagen chains.

To further evaluate whether these P4Hs produce the desired hydroxylation activity, we introduced rhCOL3α1 gene into these p4H-expressing strains, thus producing strains, GS-P4Hαβ-sigN-rhCOL3α1, GS-BaP4H-sigN-rhCOL3α1 and GS-P4Hαβ-BaP4H-sigN-rhCOL3α1, with different rhCOL3α1 copy numbers (Table S4). After methanol induction, the cell growth, collagen expression, and the assembly of the triple helix were evaluated. Table S4 summarizes the copy numbers of rhCOL3α1 gene in all co-expression strains, which range from 1 to 11 copies. Biomass analysis (OD600) of the fermentation broths showed that no appreciable growth differences among strains after 48 h of induction, indicating that neither the introduction of P4Hs nor increased collagen gene dosage adversely affected cell growth (Fig. S1). Reducing SDS-PAGE analysis revealed clear expression of a recombinant target protein migrating at ~130 kDa, corresponding to the α1 polypeptide chain, in most co-expression strains carrying high rhCOL3α1 copy numbers (e.g., >6 copies), whereas no band was detected in the control strain and in low-copy strains (e.g., <3 copies) (Fig. 1D). Non-reducing SDS-PAGE further revealed that the prominent bands above 300 kDa that co-migrate precisely with the natural hCOL3 standard in the P4H-coexpressing strains (Fig. 1E), indicating successful trimer formation. Although a very faint trimer band was also detected in the absence of P4Hs (Fig. 1E), likely attributable to the intrinsic trimerization mediated by the C-terminal Foldon domain [4, 9], these trimers were susceptible to pepsin digestion, reflecting unstable folding and insufficient structural integrity. In contrast, high-copy strains co-expressing P4Hs yielded pepsin-resistant trimers (Fig. 1E), indicative of a stable triple-helix formation within the rhCOL3 procollagen. Quantitative analysis demonstrated that P4Hαβ co-expression yielded significantly higher procollagen titers (10.4 mg/L) compared to BaP4H alone (3.8 mg/L). Notably, the synergistic co-expression of both P4Hαβ and BaP4H further increased the yield to 13.2 mg/L (Fig. 1F). However, the dual-P4H strain GS-P4Hαβ-BaP4H-SigN-rhCOL3α1 (8-copy) exhibited only a modest increase in procollagen accumulation compared to GS-P4Hαβ-SigN-rhCOL3α1 (8-copy), suggesting that P4Hαβ alone may provide sufficient hydroxylation for efficient triple helix formation.

In addition, the gel strips containing the target bands excised from the non-reducing SDS-PAGE gels were analyzed by LC-MS/MS to identify Hyp sites, which allowed us to determine the number of hydroxylated proline sites and the coverage for each strain. The distribution of Hyp residues across the rhCOL3α1 chains revealed that the dual-P4H strain achieved the highest hydroxylation coverage (128 sites), whereas BaP4H alone modified 67 proline sites and P4Hαβ alone modified 103 sites (Fig. S2). Critically, the site-specific hydroxylation efficiency, defined as the percentage of Hyp at each modified proline site, also differed markedly. In the strain GS-P4Hαβ-BaP4H-SigN-rhCOL3α1 (8-copy), 108 out of 125 modified sites exhibited a Hyp ratio exceeding 50%. By comparison, the corresponding proportions were 77 out of 103 in the strain GS-P4Hαβ-SigN-rhCOL3α1 (8 copies) and only 16 out of 67 in the strain GS- BaP4H-SigN-rhCOL3α1 (10 copies) (Fig. S3). Together with the P4H expression levels (Fig. 1C), we concluded that P4Hαβ serves as the primary hydroxylation driver in *K. phaffii*, whereas BaP4H provides a mild synergistic boost presumably by modifying different proline sites.

### Optimization of signal sequences for rhCOL3 procollagen

**Fig. 2:**
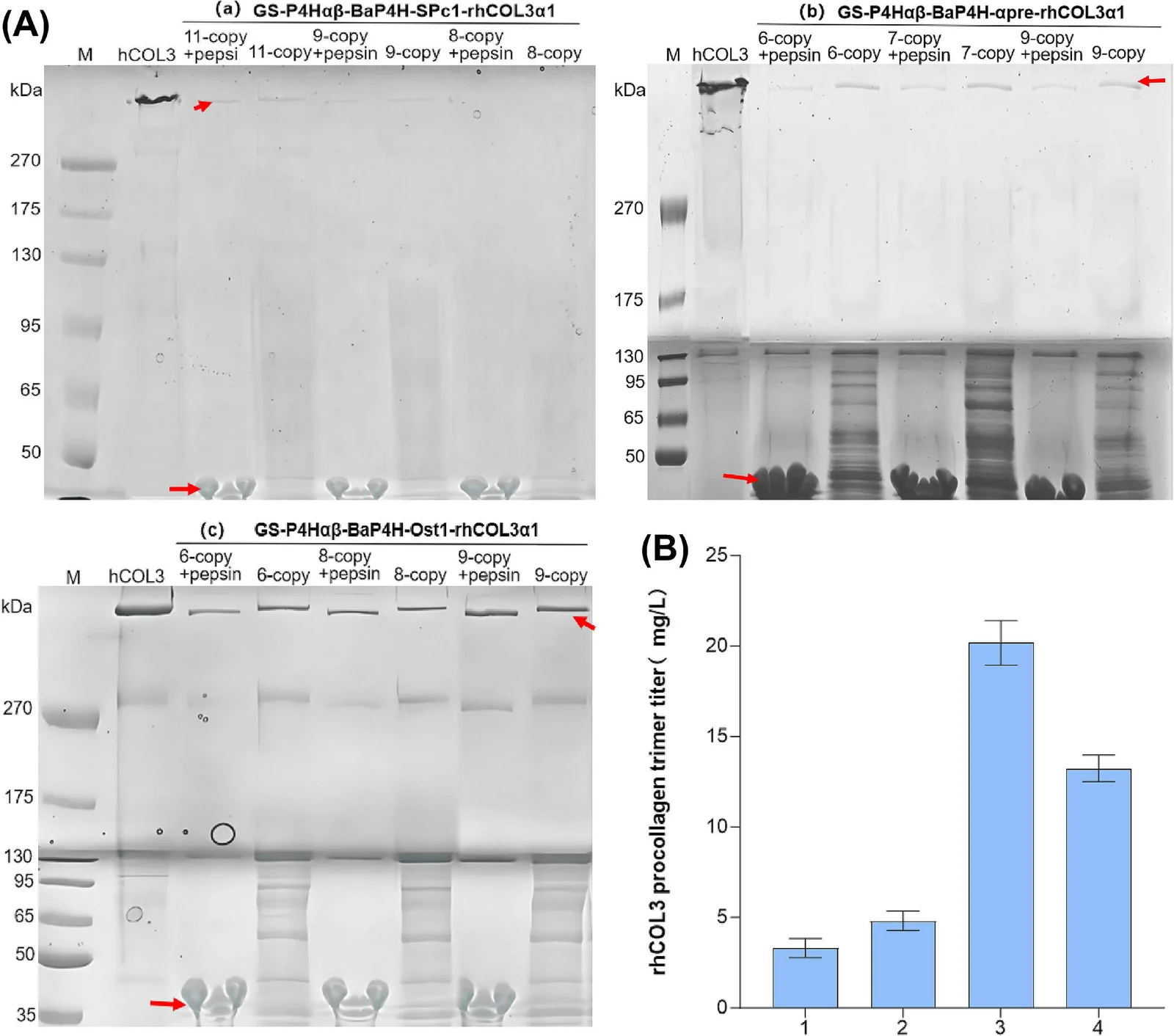
Expression analysis of rhCOL3 procollagen using alternative signal sequences. **A** Non-reducing SDS-PAGE analysis of acidified lysates treated and untreated with pepsin using a stacking gel containing 5% (w/v) acrylamide and a discontinuous separating gel. Samples were taken from three high-copy strains of each recombinant type containing an alternative signal sequence. (a) GS-P4Hαβ-BaP4H-SPc1-rhCOL3α1 strain (with 8 ~ 11 copies of the rhCOL3α1 gene); (b) GS-P4Hαβ-BaP4H-αpre-rhCOL3α1 (with 6 ~ 9 copies of the rhCOL3α1 gene); (c) GS-P4Hαβ-BaP4H-Ost1-rhCOL3α1 (with 6 ~ 9 copies of the rhCOL3α1 gene). Lane Lane M: Marker; lane hCOL3: natural human type III collagen standard (2 μg loaded, control). Arrows indicate the procollagen trimer band (> 300 kDa), and the pepsin band is visible at ~ 40 kDa. **B** Quantitative analysis of rhCOL3 procollagen trimer titers in high-copy-number strains. Only the highest titer and copy number of each recombinant strain are shown. Column 1: GS-P4Hαβ-BaP4H-SPc1-rhCOL3α1 strain (with 11 copies of the rhCOL3α1 gene); column 2: GS-P4Hαβ-BaP4H-αpre-rhCOL3α1 strain (with 9 copies of the rhCOL3α1 gene); column 3: GS-P4Hαβ-BaP4H-Ost1-rhCOL3α1 strain (with 9 copies of the rhCOL3α1 gene); column 4: GS-P4Hαβ-BaP4H-sigN-rhCOL3α1 strain (with 8 copies of the rhCOL3α1 gene). All assays were repeated at least three times and results were expressed as the mean ± SD

Signal peptides play crucial regulatory roles in recombinant protein expression by not only directing translocation of nascent polypeptides into the ER but also profoundly influencing post-translational modifications, folding, secretion efficiency, and overall expression levels [57]. Post-translational modification and trimerization of procollagen α1 chains occur naturally within the endoplasmic reticulum (ER) lumen [43], a process that requires an N-terminal signal sequence to direct post-translational translocation of the nascent polypeptide across the ER. To improve ER import of nascent rhCOL3α1 chains, we evaluated three non-secretory signal sequences (SPc1, αpre, and Ost1) in the parent strain GS-P4Hαβ-BaP4H. Both αpre and SPc1 typically mediate the post-translational translocation into the ER, but proteins prone to folding or aggregation in the cytosol may be translocated inefficiently [3]. Through plasmid reconstitution, we obtained pPICZ-SPc1-rhCOL3α1, pPICZ-αpre-rhCOL3α1, and pPICZ-Ost1-rhCOL3α1 (Table 1). Additionally, the corresponding recombinant strains, designated GS-P4Hαβ-BaP4H-SPc1-rhCOL3α1, GS-P4Hαβ-BaP4H-αpre-rhCOL3α1, and GS-P4Hαβ-BaP4H-Ost1-rhCOL3α1, were obtained by electrotransformation and high-copy screening. As a result, even at high copy numbers (9 copies for αpre and 11 copies for SPc1), the two signal sequences resulted in low accumulation of rhCOL3 procollagen, with only faint trimer bands visible on non-reducing SDS-PAGE gels (Fig. 2A). In contrast, the *S. cerevisiae* Ost1 signal sequence, which directs the co-translational translocation of nascent polypeptide chains across the ER membrane [3, 57], significantly enhanced the yield to 20.2 mg/L in the strain GS-P4Hαβ-BaP4H-Ost1-rhCOL3α1 (9-copy) (Fig. 2B), and this trimer remained stable after pepsin digestion.

### Improving rhCOL3 procollagen yield via co-expression of chaperones

**Fig. 3:**
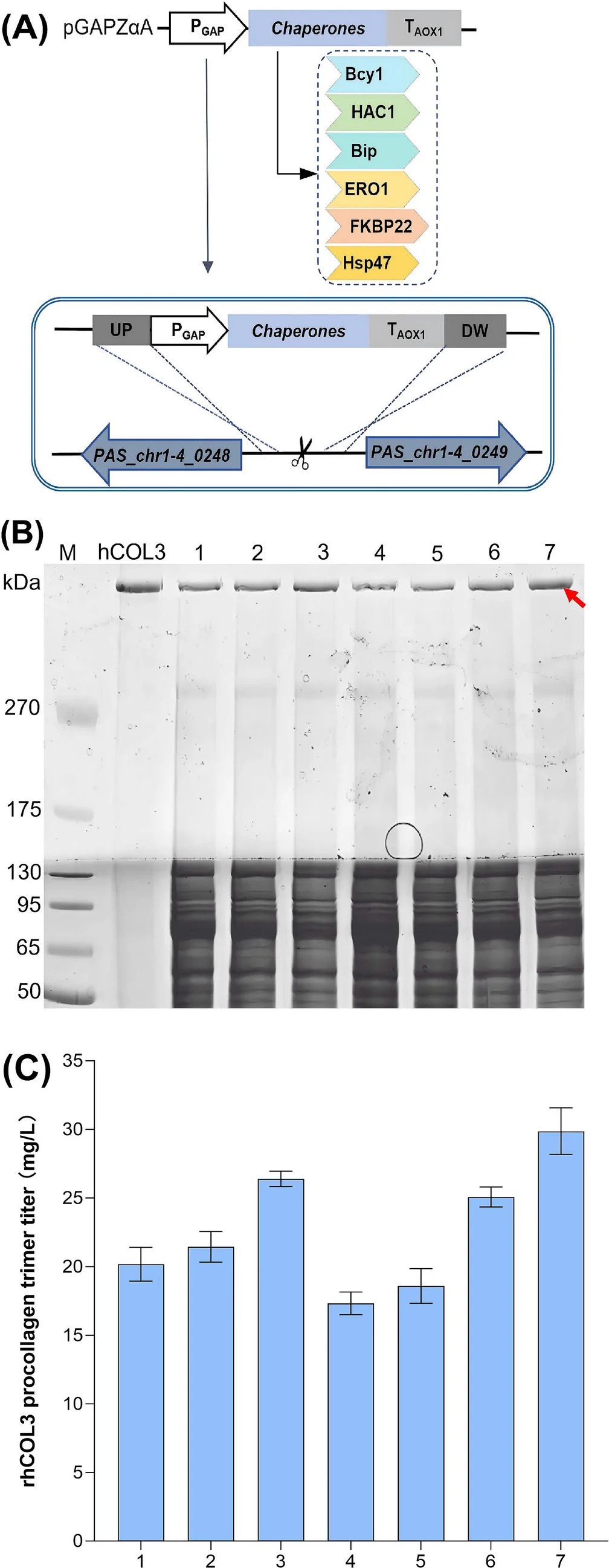
Enhancement of rhCOL3 procollagen expression via chaperone co-expression. **A** Schematic diagram of strain construction for chaperone co-expression in *K. phaffii*. UP: upstream homologous arm; DW: downstream homologous arm. **B** Non-reducing SDS-PAGE analysis of acidified cell lysates from the strains co-expressing chaperones using a stacking gel containing 5% (w/v) acrylamide and a discontinuous separating gel. Lane M: Marker; lane hCOL3: natural human type III collagen standard (2 μg loaded, control); lane 1: GSΔku70-P4Hαβ-BaP4H-Ost1-rhCOL3α1 strain; lanes 2–7: strains co-expressing FKBP22, ERO1, HAC1, BiP, HSP47, and BCY1, respectively. Arrows indicate the trimer band (> 300 kDa). **C** Quantitative analysis of rhCOL3 procollagen trimer titers in chaperone co-expressing strains. Column 1: GSΔku70-P4Hαβ-BaP4H-Ost1-rhCOL3α1 strain; Column 2–7: strains co-expressing FKBP22, ERO1, HAC1, BiP, HSP47, and BCY1, respectively. All assays were repeated at least three times and results were expressed as the mean ± SD

*K. phaffii* commonly experiences ER stress when expressing exogenous proteins, which triggers the unfolded protein response (UPR) and upregulates downstream target genes such as ERO1 and protein disulfide isomerase (PDI). The UPR can partially enhance ER protein folding [27]. Molecular chaperones maintain cellular proteostasis by promoting correct folding of nascent polypeptides, preventing aggregation, and modulating the ER redox and calcium environment, and are particularly important for alleviating ER stress during high-level recombinant protein production. In this study, based on previously established roles in protein translation [25], ER stress response [27, 49, 50], and collagen folding and assembly [19, 20], we evaluated the impact of co-expressing six chaperones (FKBP22, EROI, HAC1, Bip, Hsp47, and Bcy1) on rhCOL3 procollagen production in the strain GS-P4Hαβ-BaP4H-Ost1-rhCOL3α1 (9-copy). Using CRISPR-based genome editing, the expression cassette for each chaperone gene was individually integrated into the neutral genomic locus between *PAS_chr1-4_0248* and *PAS_chr1-4_0249* in *K. phaffii* (Fig. 3A).

Following shake‐flask fermentation, rhCOL3 procollagen production was clearly analyzed via non-reducing SDS‐PAGE (Fig. 3B). Among the tested chaperones, the co‐expression of Bcy1 gave the highest trimer titer of 29.5 mg/L, representing a 46.0% increase compared to the chaperone-free control (Fig. 3C), suggesting a pronounced enhancement of procollagen trimer folding and assembly. The co‐expression of EROI also conferred a moderate improvement in rhCOL3 procollagen yield, reaching 26.4 mg/L. Unexpectedly, other chaperones (FKBP22, HAC1, and BiP) did not show a significant promoting effect on the formation of rhCOL3 procollagen trimer.

### High-level expression in 5-L bioreactors

**Fig. 4:**
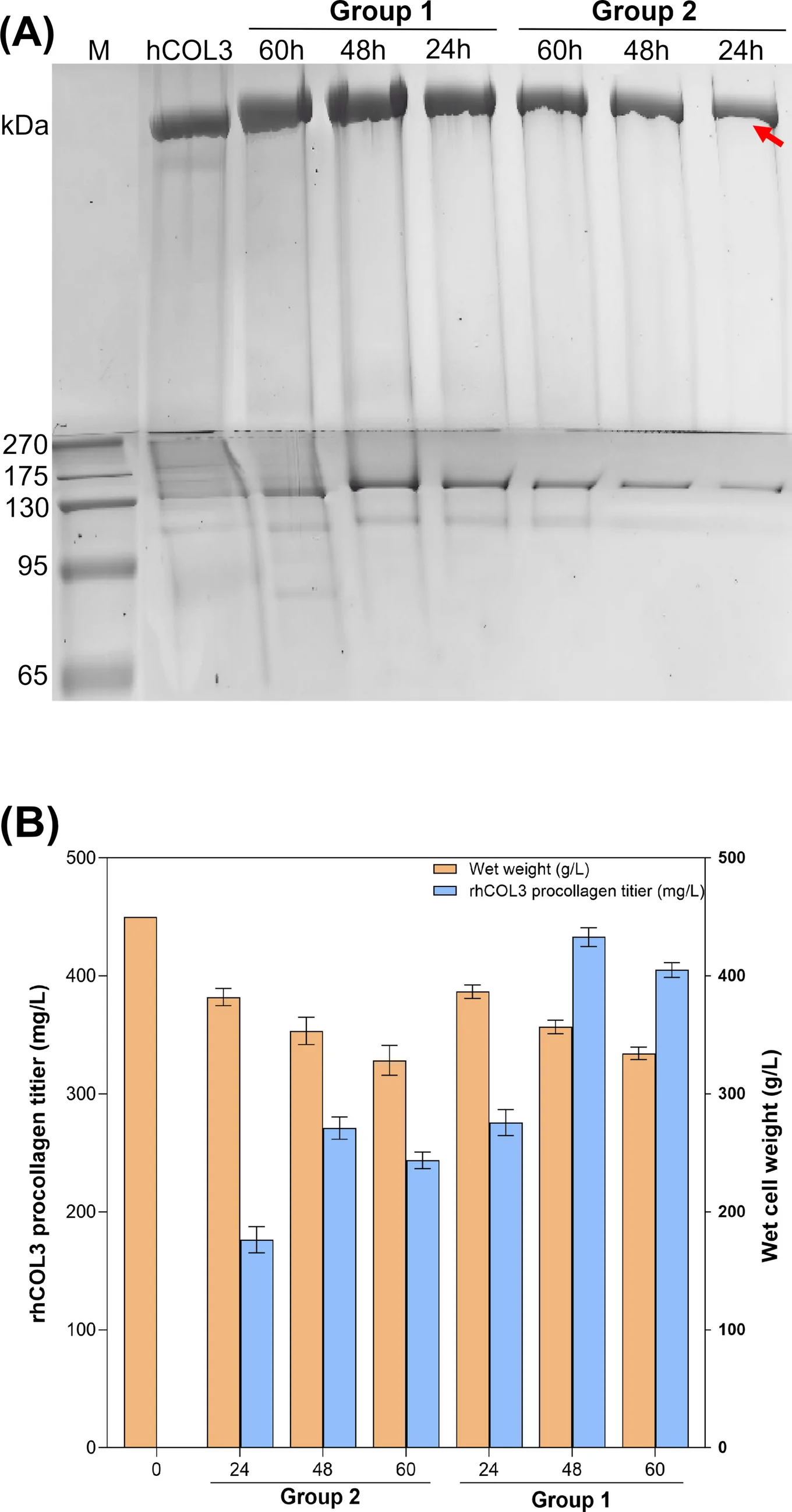
Production of rhCOL3 procollagen in 5-L bioreactors with the engineered strains. Group 1: GSΔku70-P4Hαβ-BaP4H-Ost1-rhCOL3α1-Bcy1 strain (with 9 copies of the rhCOL3α1 gene); Group 2: GS-P4Hαβ-BaP4H-SigN-rhCOL3α1strain (with 8 copies of the rhCOL3α1 gene). **A** Non-reducing SDS-PAGE analysis of acidified cell lysates at different time points of methanol induction in 5-L fed-batch fermentation. Lane M: Marker; lane hCOL3: natural human type III collagen standard (2 μg loaded, control). **B** Quantitative analysis of rhCOL3 procollagen trimer titers in the engineered strains. All assays were repeated at least three times and results were expressed as the mean ± SD

To assess the production performance at a larger scale, high-copy-number strains GS-P4Hαβ-BaP4H-SigN-rhCOL3α1 (8-copy) and GSΔku70-P4Hαβ-BaP4H-Ost1-rhCOL3α1-Bcy1 were cultivated in 5-L bioreactors using an optimized fed-batch fermentation process. Non-reducing SDS-PAGE analysis confirmed the production of rhCOL3 procollagen in both strains, as evidenced by a distinct band above 300 kDa that co-migrated with the natural hCOL3 standard (Fig. 4A), consistent with those results in shake flask experiments. Quantitative analysis revealed that the engineered strain GSΔku70-P4Hαβ-BaP4H-Ost1-rhCOL3α1-Bcy1 achieved a peak rhCOL3 procollagen titer of 430 mg/L (1.26 mg/g wet cell weight) at 48 h after methanol induction, which was 1.61 times that of the GS-P4Hαβ-BaP4H-SigN-rhCOL3α1 (8 copies) strain (267 mg/L) (Fig. 4B). However, both strains exhibited a subsequent decline in yield and biomass after 48 h, presumably attributable to progressive cell death and eventual cell lysis caused by prolonged ER stress and excessive metabolic burden [49].

### Purification of triple-helical rhCOL3

**Fig. 5:**
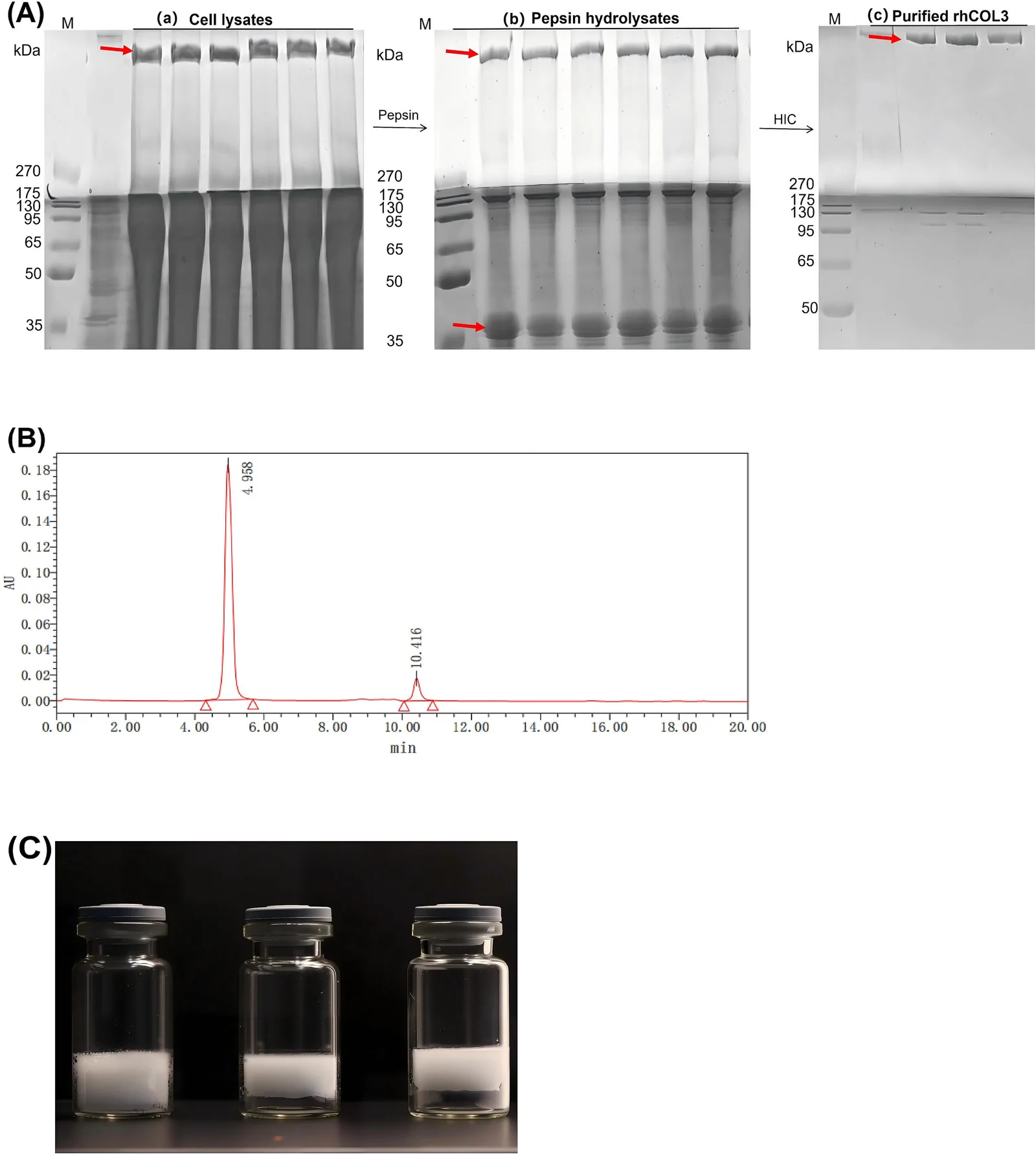
Purification of triple-helical rhCOL3. **A** Non-reducing SDS-PAGE analysis of samples from successive purification steps. (a) supernatant of cell lysates; (b) supernatant of pepsin hydrolysates; (c) purified rhCOL3. Arrows indicate the rhCOL3 trimer band (> 300 kDa) and pepsin (~ 40 kDa). **B** SEC-HPLC profile of purified rhCOL3. The peak at a retention time of 4.958 min corresponds to rhCOL3, and the peak at 10.416 min corresponds to the solvent (acetic acid). **C** Appearance of lyophilized rhCOL3

To prepare triple-helical rhCOL3 from its procollagen homotrimer, yeast cells harvested after 48 h of induction were disrupted by high-pressure homogenization, and the lysate was subjected to pepsin digestion (1.0 mg/mL) under acidic conditions. The digested product was concentrated using an ultrafiltration tube in 0.5 M acetic acid, and pepsin and pigments were removed by hydrophobic interaction chromatography. Non-reducing SDS-PAGE analysis of the purified product showed virtually no host-derived proteins, with the rhCOL3 trimer stably present (Fig. 5Ab). Additionally, a distinct monomeric band migrating at ~130 kDa was observed, likely corresponding to a minor fraction of unassembled rhCOL3α1 polypeptide chains that partially dissociated under high voltage electrophoresis. Both non-reducing SDS-PAGE and SEC-HPLC analyses indicated that the purity of the purified rhCOL3 protein exceeded 95% (Fig. 5Ac and B). Purified rhCOL3 appeared as a white solid after lyophilization (Fig. 5C).

### Structural characterization of triple-helical rhCOL3

#### Primary structure identification

**Fig. 6:**
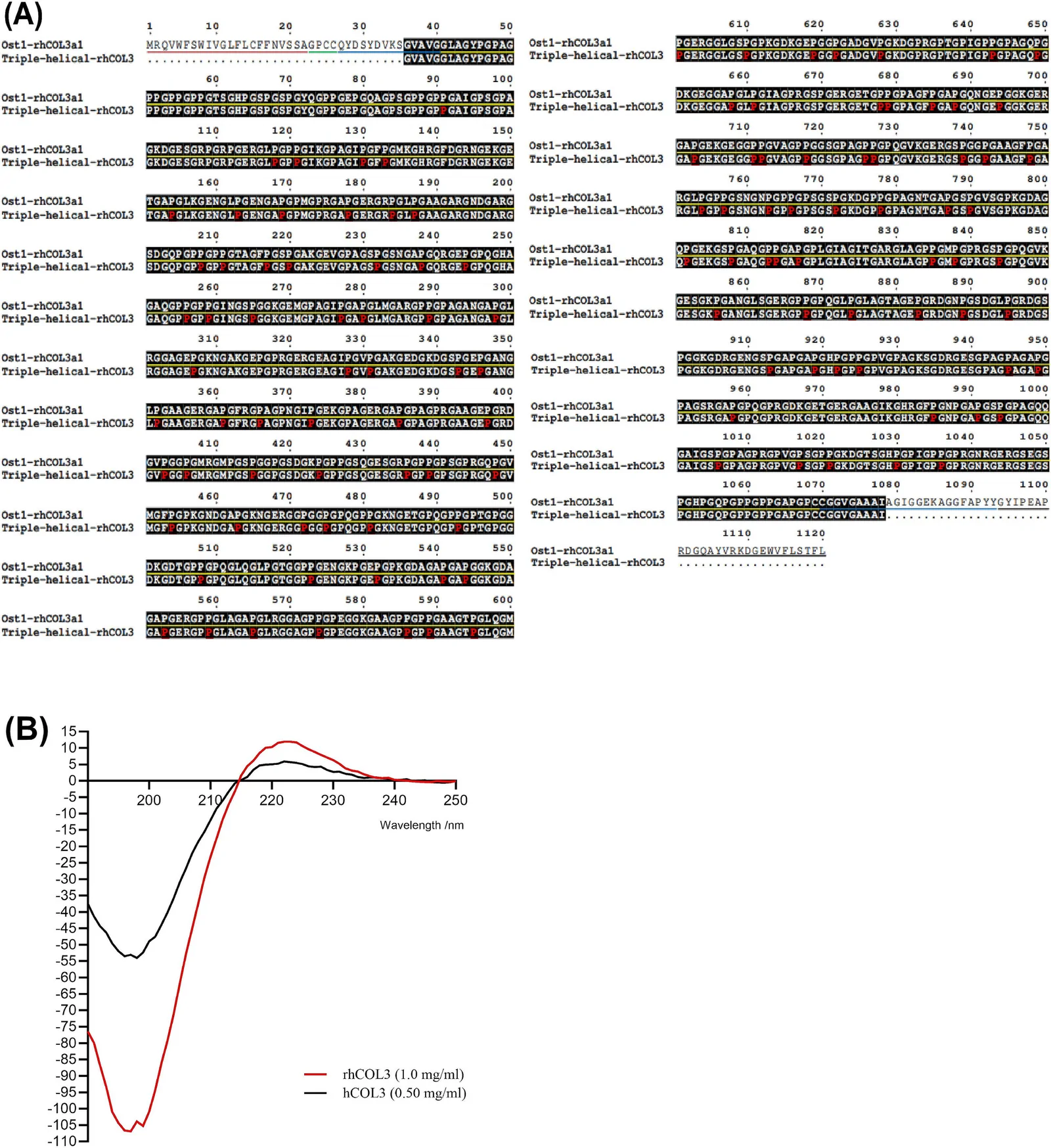

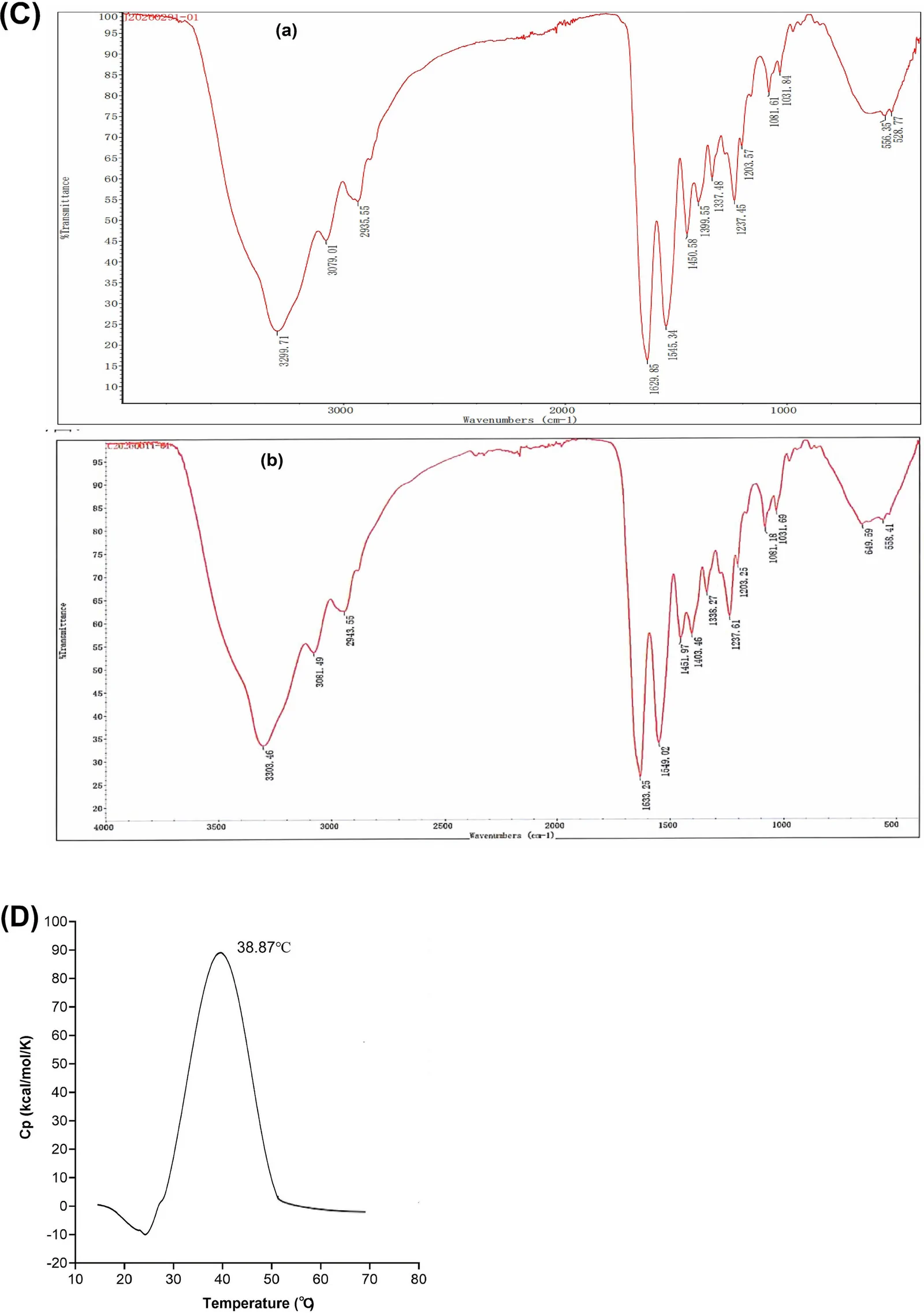

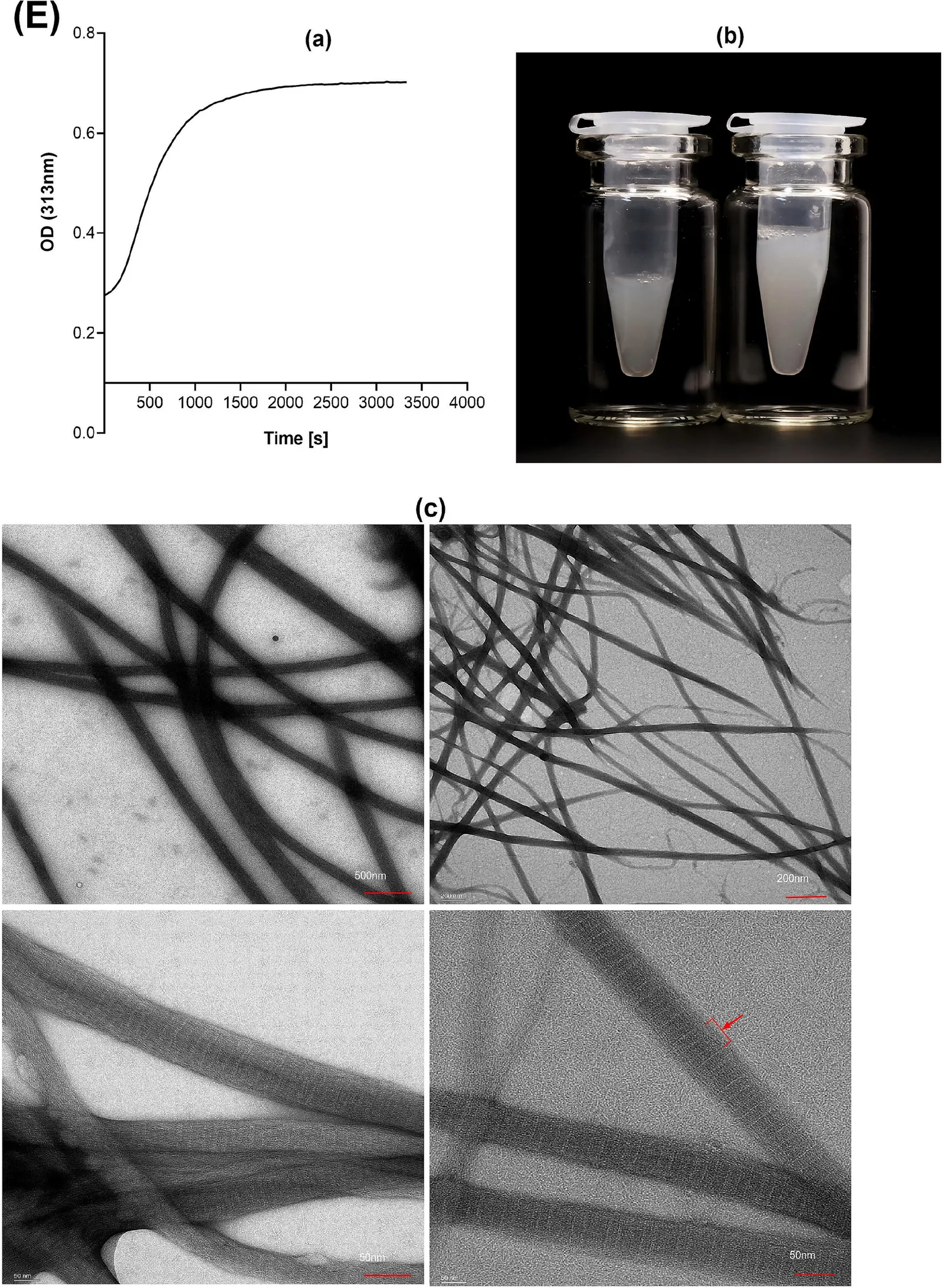
Structural characterization of triple-helical rhCOL3. **A** Theoretical sequence of the engineered rhCOL3α1 chain (marked as Ost1-rhCOLa1), and the experimentally determined sequence of purified rhCOL3 (marked as triple-helical-rhCOL3) obtained by LC-MS/MS. Underlined residues are color-coded as follows: brown, Ost1 signal sequence; green, Cys-knot motif; blue, telopeptide regions; yellow, triple-helical domain; black, Foldon domain. The sequence below the underlines corresponds to the identified sequence of purified rhCOL3, with hydroxylated proline residues highlighted in red. **B** CD spectra of purified rhCOL3 (~ 1.0 mg/mL) and natural human type III collagen standard (~ 0.5 mg/mL). **C** FTIR spectra of (a) purified rhCOL3 and (b) natural human type III collagen standard. **D** DSC thermogram of purified rhCOL3. **E** Self-assembly properties of triple-helical rhCOL3. (a) Turbidity kinetics monitored at 313 nm under a neutral pH; (b) photograph of collagen gel formed by self-assembly. (c) TEM images of collagen fibrils at 500 nm, 200 nm, and 50 nm scale

Pepsin digestion during purification removed the terminal propeptides of rhCOL3 procollagen, including the N-terminal Cys-knot motif and the C-terminal Foldon domain, to avoid potential misfolding or misassembly associated with these elements. Mass spectrometry results confirmed the precise removal of these terminal sequences while the triple-helical domain remained intact (Fig. 6A). Coverage analysis based on amino acid sequences verified the homogeneity of purified rhCOL3 with natural hCOL3, achieving a 100% peptide coverage of the triple-helical region. The N- and C-terminal regions of purified rhCOL3 only retained short truncated telopeptide tails, spanning residues from p163 to p1205 of natural hCOL3 α1 chain (GenBank: KAI4037252.1). Furthermore, as shown in Table 3, amino acid composition analysis (Table 3) showed that purified rhCOL3 was rich in glycine (22.48%), proline (10.61%), Hyp (13.56%), and arginine (7.57%), which closely mimics natural hCOL3. It was calculated that the percentage of proline hydroxylation in purified rhCOL3 reached 52.88%, very similar to the previously reported value (54%) of natural hCOL3 [37].

**Table 3:** Amino acid composition of purified rhCOL3

Number	Amino acids	Purified rhCOL3	Theoretical value from human COL3α1 chain (residues p163 ~ p1205)
1	Asn	6.12%	2.67%
2	Asp		3.04%
3	Thr	1.87%	1.57%
4	Ser	3.70%	3.88%
5	Gln	9.09%	3.08%
6	Glu		6.20%
7	Pro	10.61%	9.31%
8	Hyp	13.56%	16.93%
9	Gly	22.48%	24.20%
10	Ala	6.78%	7.36%
11	Val	1.93%	1.44%
12	Met	3.74%	1.18%
13	Ile	1.79%	1.73%
14	Leu	2.84%	2.54%
15	Tyr	0.70%	0.32%
16	Phe	1.58%	1.16%
17	Lys	4.74%	4.88%
18	His	0.88%	0.95%
19	Arg	7.57%	7.35%
20	Cys	0.22%	0.21%
21	Trp	0.00%	0.00%

Due to methodological limitations, Asn is converted to Asp, Gln is converted to Glu, and Met and Cys undergo substantial oxidation during acid hydrolysis; other amino acids are unaffecte

#### Spectroscopic characterization

Further characterization by CD spectroscopy provided insight into the conformational states of purified rhCOL3. A characteristic positive peak at 222 nm and a negative peak at 197 nm were observed in its CD spectrum (Fig. 6B), with a positive-to-negative peak ratio (Rpn) of 0.15, confirming a well-defined triple helix conformation [51]. FTIR analysis of the lyophilized sample exhibited five characteristic amide bands (Fig. 6C, a): amide A at 3303.46 cm⁻^1^, amide B at 3081.49 cm⁻^1^, amide I at 1633.25 cm⁻^1^, amide II at 1549.02 cm⁻^1^, and amide III at 1237.61 cm⁻^1^. These peaks are highly consistent with that of natural human type III collagen (Fig. 6C, b), further confirming the intact triple helix structure of rhCOL3.

#### Thermal stability and fibril formation

DSC analysis of purified rhCOL3 redissolved in 5 mM acetic acid revealed a distinct endothermic peak with a *Tm* value of 38.87 °C (Fig. 6D), nearly matching the previously reported *Tm* range of 40–43 °C for natural hCOL3 [24], demonstrating robust thermal stability. A hallmark of functional collagen is its ability to self-assemble into fibrils. The ability to self-assemble into fibrils was monitored by turbidity at 313 nm under neutral pH. Within 5 min of neutralization, the rhCOL3 solution underwent a sol–gel transition, and the OD313 entered a rapid increase phase (Fig. 6E, a). After 0.5 h, the OD313 stabilized at its maximum, and the solution gradually solidified into a gel-like state (Fig. 6E, b). TEM imaging revealed the formation of numerous visible, interwoven fibrous structures within the collagen gels. Most of these fibrils were slender and uniform within the field of view, with diameters ranging from 60 to 150 nm (Fig. 6F). Crucially, a characteristic periodic D-banding pattern, characterized by alternating light and dark regions (marked by red arrows), was also observed. These ultrastructural features confirmed that triple-helical rhCOL3 could readily form fibrils, demonstrating excellent self-assembly performance.

### Biological activity of triple-helical rhCOL3

**Fig. 7:**
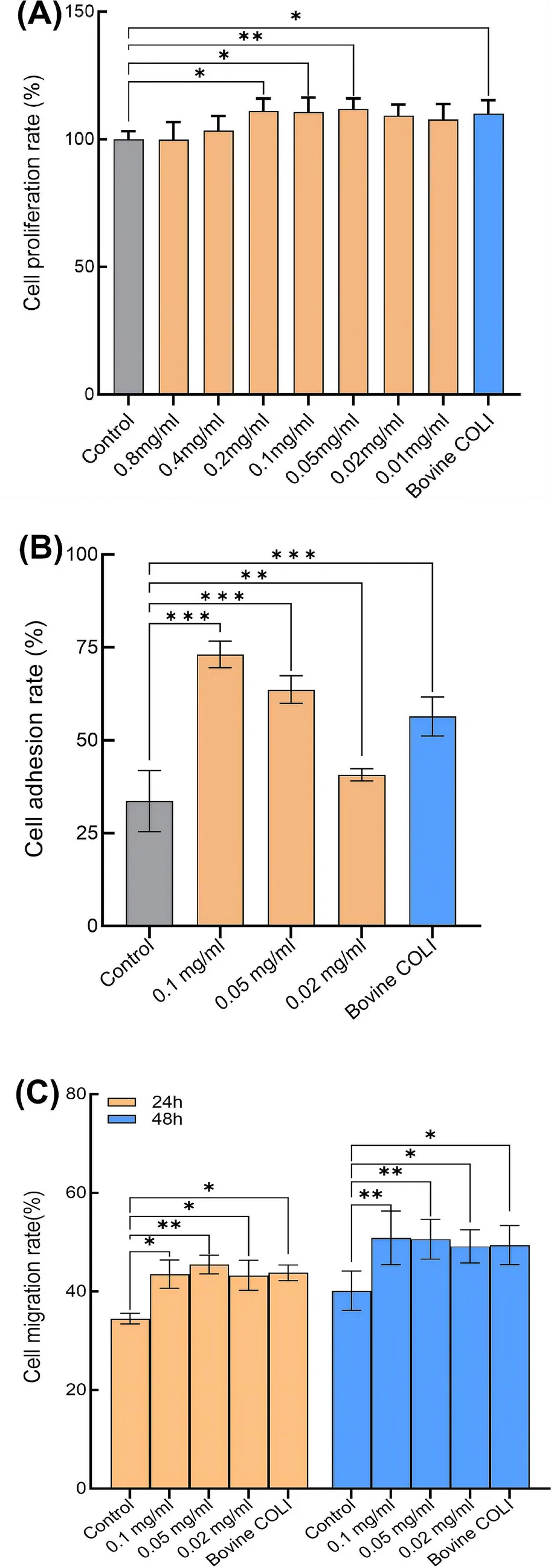

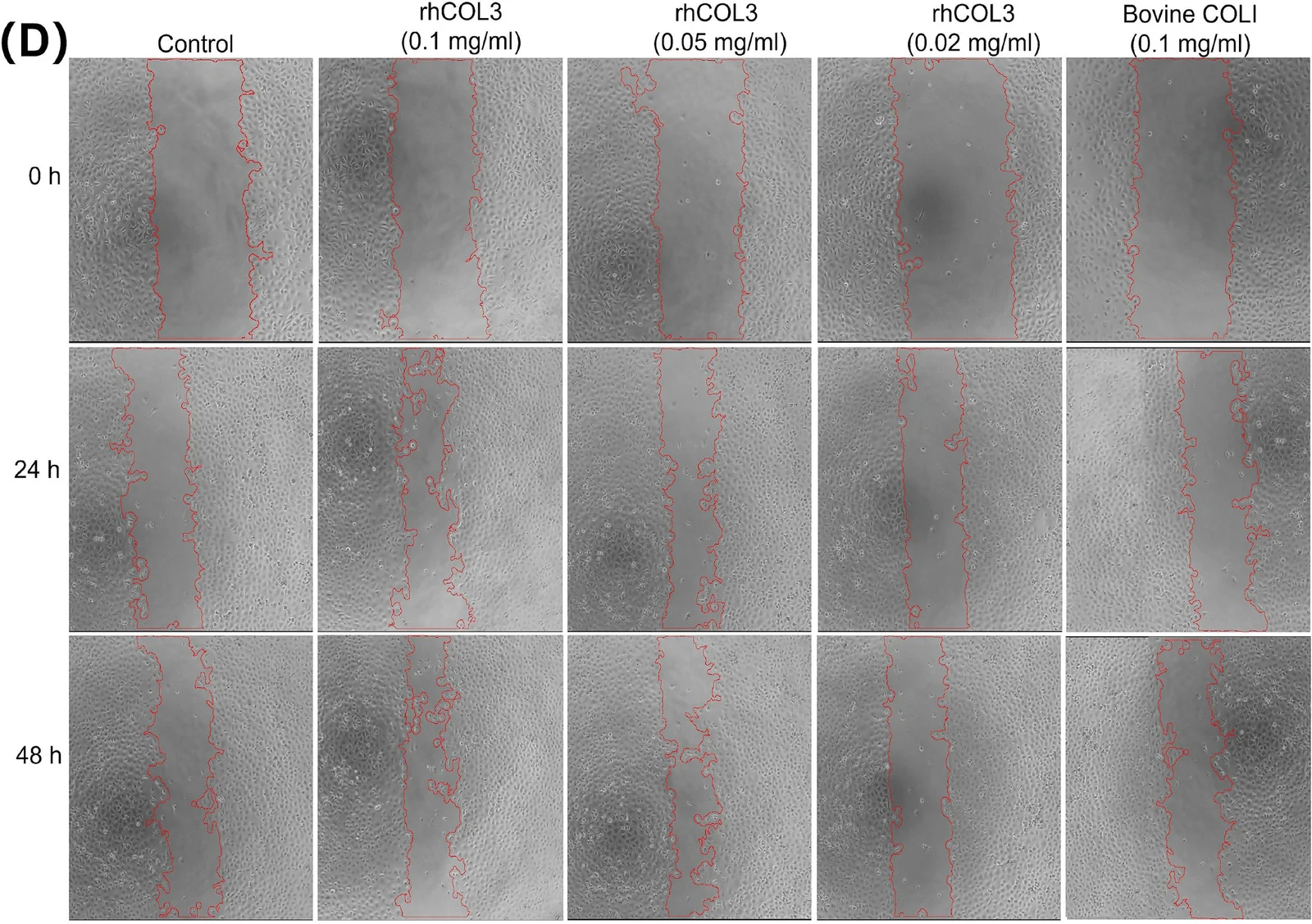
Cellular bioactivity of triple-helical rhCOL3. **A** Statistical analysis of HSF cell proliferation promoted by rhCOL3. Control: DMEM (negative); Bovine COLI: bovine type I collagen (0.1 mg/mL, positive). **B** Statistical analysis of HaCaT cell adhesion promoted by rhCOL3. Control: PBS (negative); Bovine COLI (0.1 mg/mL, positive). **C** Statistical analysis of HaCaT cell migration promoted by rhCOL3. Control: DMEM (negative); Bovine COLI (0.1 mg/mL, positive). **D** Representative micrographs of HaCaT cell migration at different time points. Data are presented as mean ± SD (*n* = 3). *P < 0.05, **P < 0.01, and ***P < 0.001

Cell proliferation, adhesion, and migration represent highly complex and precisely regulated biochemical processes frequently used to elucidate the functional properties of recombinant collagen. As illustrated in Fig. 7A, CCK-8 assay revealed that triple-helical rhCOL3 promoted HSF cell proliferation in a concentration-dependent manner within the range of approximately 0.02 ~ 0.1 mg/mL. The relative cell proliferation rates in the 0.05, 0.1, and 0.2 mg/mL rhCOL3 groups were significantly higher than that of the DMEM control group (*p* < 0.05), indicating that the recombinant human collagen had no cytotoxic effect and actively supported cell proliferation. Moreover, no statistically significant difference was observed in relative cell proliferation rates between the rhCOL3 (0.1 mg/mL) and bovine type I collagen (0.1 mg/mL) groups (*p* > 0.05), suggesting comparable bioactivity. In cell adhesion assays, rhCOL3 at 0.1 mg/mL exhibited the strongest effect, with an adhesion rate 2.05-fold that of the control (Fig. 7B) and superior to that of bovine type I collagen at the same concentration. HaCaT cell migration was assessed by scratch assay with microscopic imaging (Fig. 7C). After 24 h of culture, the relative migration rates in the rhCOL3 (0.05 mg/mL) and rhCOL3 (0.1 mg/mL) groups were 43.25% and 44.86% (Fig. 7D), respectively, both obviously higher than that of the PBS control group (34.30%; *p* < 0.05). After 48 h, the cell migration rates in the rhCOL3 (0.02, 0.05, and 0.1 mg/mL) groups were 47.23%, 50.42%, and 51.86%, respectively, all markedly higher than that of the PBS control group (40.14%; *p* < 0.05). Collectively, these findings demonstrate that our triple-helical rhCOL3 exerts a positive regulatory effect on HaCaT cell migration.

## Discussion

Collagen is distinguished by its unique triple helix structure and is valued for its excellent mechanical properties, biocompatibility, and biodegradability, which have led to its widespread application in biomaterials and medical devices [26, 35, 43, 46]. Previous efforts to produce recombinant collagen have largely focused on the expression of collagen fragments or collagen mimetic peptides in prokaryotic and eukaryotic hosts [4, 9, 16, 30, 53]; however, efficient expression of full-length, triple-helical collagen has rarely been reported. This gap reflects the stringent structural requirements of natural collagen: a stable triple helix depends critically on site-specific proline hydroxylation and chaperone-assisted folding. Given the proven capacity of *K. phaffii* for high-cell-density fermentation and eukaryotic post-translational modifications, it was chosen as the expression host. Since wild-type *K. phaffii* lacks endogenous prolyl 4-hydroxylase activity, functional hydroxylation must be reconstituted exogenously. To address this, in this study, we systematically compared the co-expression of two P4Hs and assessed their effects on proline hydroxylation and rhCOL3 procollagen yield. Although a distinct band for BaP4H was not observed on SDS-PAGE gel, LC-MS/MS analysis detected hydroxyproline residues consistent with bacterial P4H activity in the recombinant collagen. This enzymatic footprint provides clear evidence that BaP4H was successfully expressed and functional in the engineered strains. The differential performance of P4Hαβ and BaP4H can be understood in light of their distinct evolutionary origins and substrate recognition mechanisms. Human P4Hαβ, as a collagen-specific hydroxylase, likely recognizes and modifies a broader repertoire of proline sites within the Gly-X-Y repeat context, whereas the bacterial BaP4H, evolved for distinct substrates, may exhibit more restricted sequence specificity. This is supported by our MS data showing that P4Hαβ alone modified nearly twice as many sites as BaP4H (103 vs. 67). Although synergistic co-expression of both enzymes increased the number of high-occupancy hydroxylation sites (i.e., > 50% Hyp, increased by 31 sites), the overall gain in trimer yield was modest. This suggests that the hydroxylation capacity provided by P4Hαβ may approach saturation under the conditions tested and that further improvements in procollagen production are likely limited by bottlenecks downstream of hydroxylation, such as chain registration or ER-to-Golgi trafficking.

To further improve procollagen trimer production, two molecular engineering strategies, including signal peptide substitution and chaperone co-expression, were investigated. Since the assembly of the collagen triple helix is a highly coordinated process that depends on multiple ER-resident molecular chaperones and folding enzymes, the correct folding and assembly of procollagen molecules occur exclusively intracellularly. Moreover, the full-length, triple-helical procollagen protein exhibits an exceptionally high molecular weight (> 300 kDa), and yeast cells are inherently inefficient at secreting such large proteins, leading to intracellular accumulation. Non-secretory signal peptides were employed in this study to direct translocation into the rough ER lumen [47]. Translocation efficiency of the nascent rhCOL3α1 chain varied markedly among the tested signal sequences. Both SigN and SPc1 from natural human collagen support post-translational translocation, as does the *S. cerevisiae* α-mating factor pre-region (αpre). With these three signal sequences, clear target protein expression was detected (Fig. 2A), confirming their functionality in transmembrane translocation. However, the lower titers of procollagen trimer still revealed their limited translocation efficiency in the strains GS-P4Hαβ-BaP4H-SPc1-rhCOL3α1 and GS-P4Hαβ-BaP4H-αpre-rhCOL3α1. Fortunately, Ost1, which directs co-translational translocation, markedly increased procollagen yield relative to SigN (Fig. 2B), although the precise mechanism remains to be determined. The rhCOL3α1 chain, comprising over 1000 amino acids with extensive Gly-X-Y repeats, is predicted to fold rapidly or form aggregates in the cytosol. Under these conditions, post-translational translocation, which requires the polypeptide to remain unfolded for ER import, becomes inefficient [3]. The Ost1-mediated co-translational pathway circumvents this limitation by coupling translocation to ongoing polypeptide elongation, thereby minimizing the cytosolic residence time of aggregation-prone folding intermediates. This finding aligns with recent reports that co-translational signal sequences enhance the production of other difficult-to-express proteins in yeast [57].

Collagen-specific chaperones such as FKBP22 and HSP47, although essential in mammalian cells [19, 20], did not improve procollagen trimer production when overexpressed in yeast (Fig. 3B, C), suggesting that certain endogenous folding factors could partially compensate for their absence. In contrast, the co-expression of BCY1 and ERO1, which are not directly involved in collagen biosynthesis, significantly increased intracellular procollagen accumulation (Fig. 3C). From a global perspective, Bcy1, a regulatory subunit of protein kinase A that functions as a global translational regulator, can promote cell growth and heterologous protein expression by modulating ribosomal protein genes and stress-responsive genes [25], which likely restored cellular viability and metabolic capacity from ER stress. Similarly, ERO1 acting as a key downstream UPR effector to establish an oxidative ER environment and activate PDI. PDI can catalyze the formation of interchain disulfide bonds among recombinant rhCOL3α1 chains [50] and simultaneously serves as the β-subunit of the P4H α2β2 tetramer to stabilize the hydroxylation activity [21, 47, 48], thereby promoting the proline hydroxylation, subsequent oxidative folding, and trimeric assembly of rhCOL3 procollagen. Therefore, appropriate chaperone co-expression can not only influence polypeptide folding and structural integrity but also significantly boost expression levels by optimizing translation and mitigating cellular stress. In 5-L fed-batch bioreactors, high-density fermentation enabled substantial biomass accumulation, thereby significantly enhancing procollagen production. Consequently, the procollagen titers achieved by the strains GS-P4Hαβ-BaP4H-SigN-rhCOL3α1 and GSΔku70-P4Hαβ-BaP4H-Ost1-rhCOL3α1-Bcy1 markedly exceeded those obtained in shake-flask cultures. The maximum titer reached 430 mg/L (Fig. 4B), significantly exceeding the yield of full-length human type I collagen reported by [51] using CHO cells (~ 0.3 mg/mL). Although this result represents a substantial advancement and provides a basis for downstream process development, the yield remains below the threshold required for cost-effective industrial production.

To obtain triple-helical rhCOL3, we adapted a purification protocol inspired by methods for animal tissue-derived collagen [31, 35]. Selective pepsin digestion removed host proteins and completely cleaved the N- and C-terminal propeptides from the procollagen trimer (Fig. 6A). Subsequent ultrafiltration and hydrophobic interaction chromatography eliminated residual pepsin and other impurities, yielding a high-purity lyophilized product (Fig. 5). Mass spectrometry analysis confirmed that the purified product consisted of the full-length triple-helical domain of the human COL3α1 chain, with only short residual telopeptides at the termini (Fig. 6A). Amino acid composition analysis revealed a Hyp content of 13.56%, closely matching that of natural hCOL3 (Table 3), providing robust biochemical evidence for faithful proline hydroxylation. Both CD and DSC revealed a properly folded triple helix (Fig. 6B, D). Stable triple-helical collagens can self-assemble into higher-order fibrils, a property essential for biological function [4, 9]. TEM revealed fibrils exhibiting the typical axial D-periodicity (Fig. 6F), consistent with the definitive ultrastructural hallmark of natural collagen fibrils. Taken together, these results confirm that rhCOL3 adopts a native-like triple-helical conformation, assembles with proper intermolecular packing, and recapitulates the hierarchical self-assembly of natural collagen, thereby supporting its potential for biomaterial applications. Biologically, collagen regulates fibroblast proliferation, migration, and adhesion primarily through specific binding motifs such as GFOGER [39, 43, 56]. In our functional assays, it was observed that triple-helical rhCOL3 significantly enhanced HSF proliferation, HaCaT adhesion, and migration at physiologically relevant concentrations of 0.02–0.1 mg/mL (Fig. 7). However, the stimulatory effect plateaued above 0.4 mg/mL, which might be due to receptor saturation or steric hindrance, thereby possibly limiting the application of triple-helical rhCOL3 in medical scenarios requiring high-dose administration.

## Conclusion

In summary, this study established a systematic platform for producing full-length, triple-helical recombinant human type III collagen in *K. phaffii*, achieving a titer of 430 mg/L in fed-batch bioreactors. The purified collagen faithfully recapitulates the primary sequence, proline hydroxylation profile, thermal stability, and hierarchical fibril-forming capacity of native human type III collagen and supports fibroblast proliferation, adhesion, and migration at levels comparable to natural collagen. These properties position this recombinant collagen as a promising candidate for regenerative medicine, medical aesthetics, and advanced wound healing applications. Future efforts, including engineering of the secretory pathway, dynamic regulation of global transcriptional and metabolic regulators, and AI-guided optimization of medium composition and fermentation parameters, are expected to alleviate cellular metabolic burden and further enhance productivity. Overall, this work provides an effective platform for producing recombinant human collagen with a stable, native-like triple helix.

## Supplementary Information

Below is the link to the electronic supplementary material.

ESM 1 (PDF 5.90 MB)

ESM 2 (DOCX 16.5 MB)

## Data Availability

No datasets were generated or analysed during the current study.
