# Supplementary material for: Enhanced biosynthesis of full-length triple-helical human type III collagen in engineered Komagataella phaffii
Source: Appl Microbiol Biotechnol. 2026 Aug 29;110:300. doi: 10.1007/s00253-026-13996-7 (PMC13646244; doi:10.1007/s00253-026-13996-7)
Supplement: Supplementary file 1 [file 253_2026_13996_MOESM1_ESM.pdf]

Supplementary materials

Enhanced biosynthesis of full-length triple-helical human type III collagen in engineered *Komagataella phaffii*

Zhijian Ni [1,2] • Xiaolu Yu [2] • Qi Da [2] • Xiaoxi Wu [2] • ZhiDa Wei [2] • Yulei Hu [2] • Yao Xiao [4] • Chunting Ye [2] • Chunlin Deng [1,3,*]

Fig. S1. Growth profiles of recombinant strains harboring different rhCOL3α1 copy numbers. Biomass (OD600) was measured after 48 h of methanol-induced shake-flask fermentation.

Fig. S2. Distribution of hydroxyproline residues across the rhCOL3α1 chain in recombinant strains co-expressing collagen with different P4Hs, as identified by LC-MS/MS. Hydroxylated proline sites are color-coded as follows: yellow, 67 sites in strain GS-BaP4H-SigN-rhCOL3α1 (10-copy); blue, 103 sites in strain GS-P4Hαβ-SigN-rhCOL3α1 (8-copy); and red, 128 sites in strain GS-P4Hαβ-BaP4H-SigN-rhCOL3α1 (8-copy).

Fig. S3. Site-specific hydroxylation efficiency (defined as the percentage of Hyp at each modified proline residue) of the rhCOL3α1 chain in recombinant strains co-expressing collagen with P4Hs, determined by LC-MS/MS. Specifically, the target bands (>300 kDa) from non-reducing SDS-PAGE gels of three high-copy strains (GS-BaP4H-sigN-rhCOL3α1, GS-P4Hαβ-sigN-rhCOL3α1, and GS-P4Hαβ-BaP4H-sigN-rhCOL3α1) were excised, reduced with 10 mM DTT, alkylated with 50 mM iodoacetamide, and followed by trypsin, chymotrypsin and

Glu-C. The resulting peptides were analyzed by LC-MS/MS to identify Hyp sites. For each identified Hyp site, hydroxylation ratio was determined from extracted ion chromatograms of the modified and unmodified peptides using PEAKS Studio 13. The peak area of the Hyp-containing peptide (S_{mod}) and that of the corresponding non-hydroxylated peptide (S_{umod}) were integrated. The site-specific hydroxylation efficiency is expressed as: Hydroxylation efficiency (%) = S_{mod} / (S_{mod} + S_{umod})] × 100. **(A)** Strain GS-BaP4H-SigN-rhCOL3α1 (10-copy): 16 of 67 modified sites exhibited a Hyp content exceeding 50%. **(B)** Strain GS-P4Hαβ-SigN-rhCOL3α1 (8-copy): 77 of 103 modified sites exhibited a Hyp content exceeding 50%. **(C)** Strain GS-P4Hαβ-BaP4H-SigN-rhCOL3α1 (8-copy): 108 of 125 modified sites exhibited a Hyp content exceeding 50%.

Fig. S1

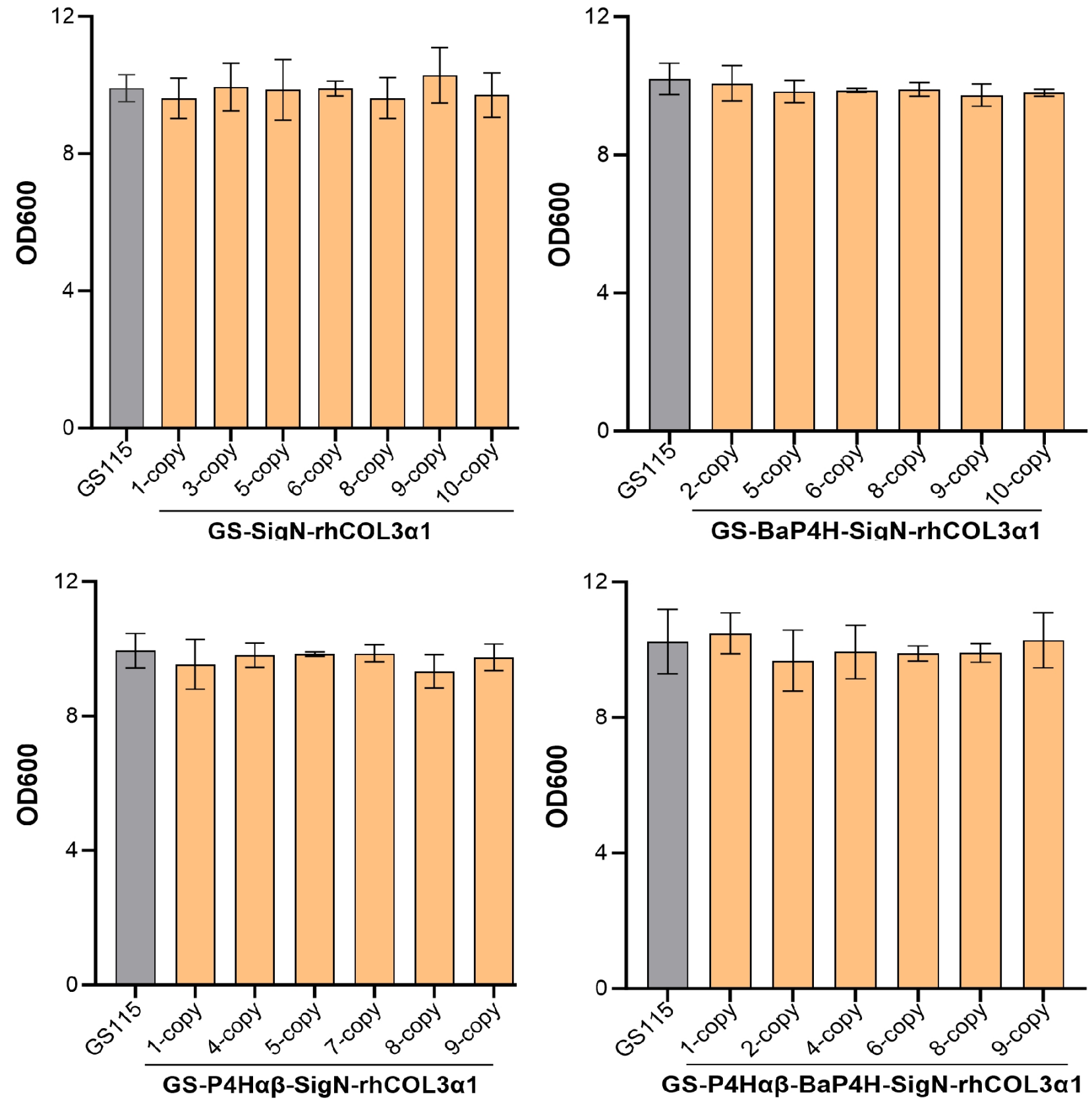

OD600
0
4
8
12
GS115
1-copy
3-copy
5-copy
6-copy
8-copy
9-copy
10-copy
GS-SigN-rhCOL3α1
GS115
2-copy
5-copy
6-copy
8-copy
9-copy
10-copy
GS-BaP4H-SigN-rhCOL3α1
GS115
1-copy
4-copy
5-copy
7-copy
8-copy
9-copy
GS-P4Hαβ-SigN-rhCOL3α1
GS115
1-copy
2-copy
4-copy
6-copy
8-copy
9-copy
GS-P4Hαβ-BaP4H-SigN-rhCOL3α1

Fig. S2

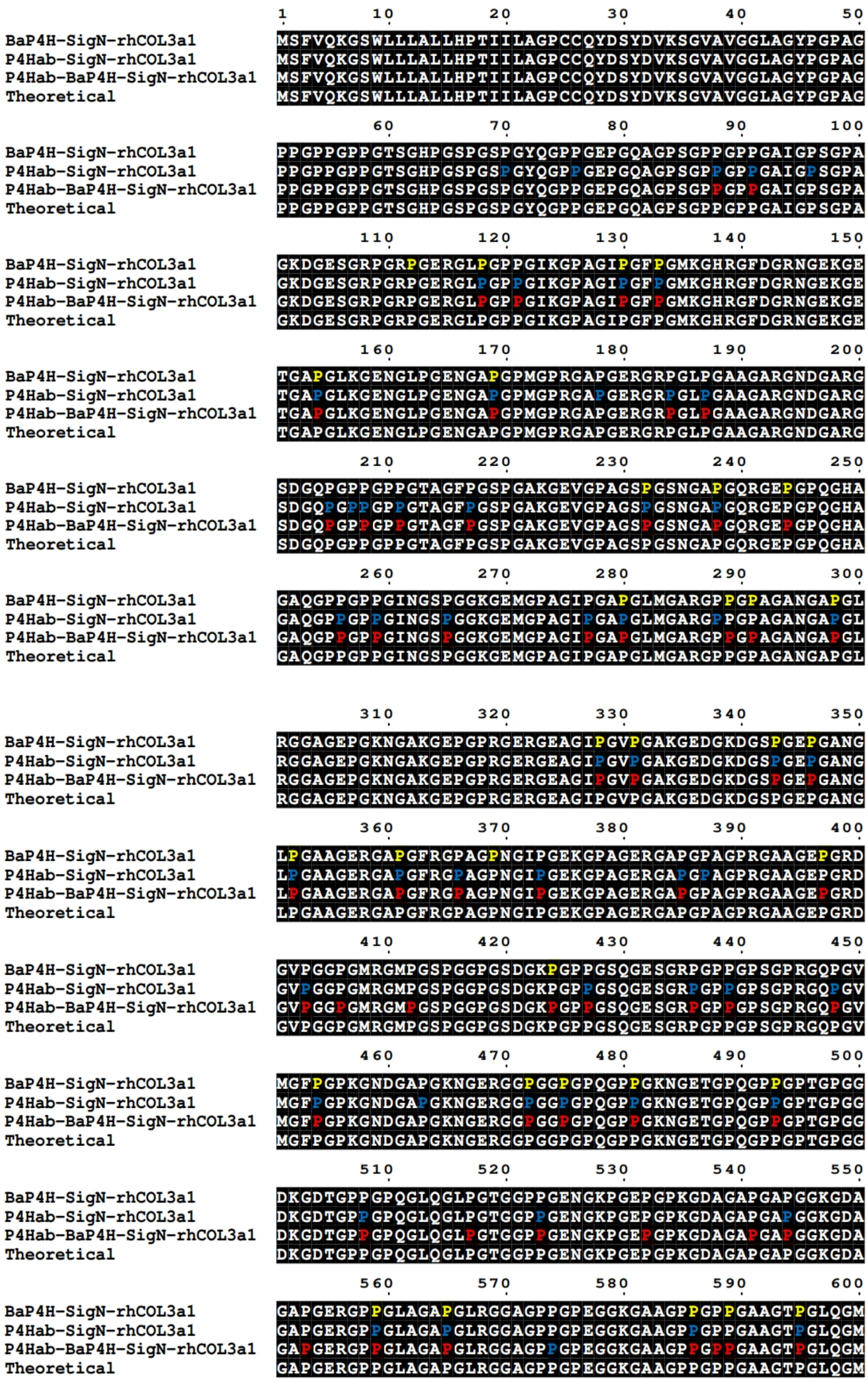
1 10 20 30 40 50
BaP4H-SigN-rhCOL3a1 MSFVQKGSWLLLALLHPTIILAGPCCQYDSYDVKSGVAVGGLAGYPGPAG
P4Hab-SigN-rhCOL3a1 MSFVQKGSWLLLALLHPTIILAGPCCQYDSYDVKSGVAVGGLAGYPGPAG
P4Hab-BaP4H-SigN-rhCOL3a1 MSFVQKGSWLLLALLHPTIILAGPCCQYDSYDVKSGVAVGGLAGYPGPAG
Theoretical MSFVQKGSWLLLALLHPTIILAGPCCQYDSYDVKSGVAVGGLAGYPGPAG
60 70 80 90 100
BaP4H-SigN-rhCOL3a1 PPGPPGPPGTSGHPGSPGSPGYQGPPGEPGQAGPSGPPGPPGAIGPSGPA
P4Hab-SigN-rhCOL3a1 PPGPPGPPGTSGHPGSPGSPGYQGPPGEPGQAGPSGPPGPPGAIGPSGPA
P4Hab-BaP4H-SigN-rhCOL3a1 PPGPPGPPGTSGHPGSPGSPGYQGPPGEPGQAGPSGPPGPPGAIGPSGPA
Theoretical PPGPPGPPGTSGHPGSPGSPGYQGPPGEPGQAGPSGPPGPPGAIGPSGPA
110 120 130 140 150
BaP4H-SigN-rhCOL3a1 GKDGESGRPGRPGERGLPGPPGIKGPAGIPGFPGMKGHRGFDGRNGEKGE
P4Hab-SigN-rhCOL3a1 GKDGESGRPGRPGERGLPGPPGIKGPAGIPGFPGMKGHRGFDGRNGEKGE
P4Hab-BaP4H-SigN-rhCOL3a1 GKDGESGRPGRPGERGLPGPPGIKGPAGIPGFPGMKGHRGFDGRNGEKGE
Theoretical GKDGESGRPGRPGERGLPGPPGIKGPAGIPGFPGMKGHRGFDGRNGEKGE
160 170 180 190 200
BaP4H-SigN-rhCOL3a1 TGAPGLKGENGLPGENGAPGPMGPRGAPGERGRPGLPGAAGARGNDGARG
P4Hab-SigN-rhCOL3a1 TGAPGLKGENGLPGENGAPGPMGPRGAPGERGRPGLPGAAGARGNDGARG
P4Hab-BaP4H-SigN-rhCOL3a1 TGAPGLKGENGLPGENGAPGPMGPRGAPGERGRPGLPGAAGARGNDGARG
Theoretical TGAPGLKGENGLPGENGAPGPMGPRGAPGERGRPGLPGAAGARGNDGARG
210 220 230 240 250
BaP4H-SigN-rhCOL3a1 SDGQPGPPGPPGTAGFPGSPGAKGEVGPAGSPGSNGAPGQRGEPGPQGHA
P4Hab-SigN-rhCOL3a1 SDGQPGPPGPPGTAGFPGSPGAKGEVGPAGSPGSNGAPGQRGEPGPQGHA
P4Hab-BaP4H-SigN-rhCOL3a1 SDGQPGPPGPPGTAGFPGSPGAKGEVGPAGSPGSNGAPGQRGEPGPQGHA
Theoretical SDGQPGPPGPPGTAGFPGSPGAKGEVGPAGSPGSNGAPGQRGEPGPQGHA
260 270 280 290 300
BaP4H-SigN-rhCOL3a1 GAQGPPGPPGINGSPGGKGEMGPAGIPGAPGLMGARGPPGPAGANGAPGL
P4Hab-SigN-rhCOL3a1 GAQGPPGPPGINGSPGGKGEMGPAGIPGAPGLMGARGPPGPAGANGAPGL
P4Hab-BaP4H-SigN-rhCOL3a1 GAQGPPGPPGINGSPGGKGEMGPAGIPGAPGLMGARGPPGPAGANGAPGL
Theoretical GAQGPPGPPGINGSPGGKGEMGPAGIPGAPGLMGARGPPGPAGANGAPGL
310 320 330 340 350
BaP4H-SigN-rhCOL3a1 RGGAGEPGKNGAKGEPGPRGERGEAGIPGVPGAKGEDGKDGSPGEPGANG
P4Hab-SigN-rhCOL3a1 RGGAGEPGKNGAKGEPGPRGERGEAGIPGVPGAKGEDGKDGSPGEPGANG
P4Hab-BaP4H-SigN-rhCOL3a1 RGGAGEPGKNGAKGEPGPRGERGEAGIPGVPGAKGEDGKDGSPGEPGANG
Theoretical RGGAGEPGKNGAKGEPGPRGERGEAGIPGVPGAKGEDGKDGSPGEPGANG
360 370 380 390 400
BaP4H-SigN-rhCOL3a1 LPGAAGERGAPGFRGPAGPNGIPGEKGPAGERGAPGPAGPRGAAGEPGRD
P4Hab-SigN-rhCOL3a1 LPGAAGERGAPGFRGPAGPNGIPGEKGPAGERGAPGPAGPRGAAGEPGRD
P4Hab-BaP4H-SigN-rhCOL3a1 LPGAAGERGAPGFRGPAGPNGIPGEKGPAGERGAPGPAGPRGAAGEPGRD
Theoretical LPGAAGERGAPGFRGPAGPNGIPGEKGPAGERGAPGPAGPRGAAGEPGRD
410 420 430 440 450
BaP4H-SigN-rhCOL3a1 GVPGGPGMRGMPGSPGGPGSDGKPGPPGSQGESGRPGPPGPSGPRGQPGV
P4Hab-SigN-rhCOL3a1 GVPGGPGMRGMPGSPGGPGSDGKPGPPGSQGESGRPGPPGPSGPRGQPGV
P4Hab-BaP4H-SigN-rhCOL3a1 GVPGGPGMRGMPGSPGGPGSDGKPGPPGSQGESGRPGPPGPSGPRGQPGV
Theoretical GVPGGPGMRGMPGSPGGPGSDGKPGPPGSQGESGRPGPPGPSGPRGQPGV
460 470 480 490 500
BaP4H-SigN-rhCOL3a1 MGFPGPKGNDGAPGKNGERGGPGGPGPQGPPGKNGETGPQGPPGPTGPGG
P4Hab-SigN-rhCOL3a1 MGFPGPKGNDGAPGKNGERGGPGGPGPQGPPGKNGETGPQGPPGPTGPGG
P4Hab-BaP4H-SigN-rhCOL3a1 MGFPGPKGNDGAPGKNGERGGPGGPGPQGPPGKNGETGPQGPPGPTGPGG
Theoretical MGFPGPKGNDGAPGKNGERGGPGGPGPQGPPGKNGETGPQGPPGPTGPGG
510 520 530 540 550
BaP4H-SigN-rhCOL3a1 DKGDTGPPGPQGLQGLPGTGGPPGENGKPGEPGPKGDAGAPGAPGGKGDA
P4Hab-SigN-rhCOL3a1 DKGDTGPPGPQGLQGLPGTGGPPGENGKPGEPGPKGDAGAPGAPGGKGDA
P4Hab-BaP4H-SigN-rhCOL3a1 DKGDTGPPGPQGLQGLPGTGGPPGENGKPGEPGPKGDAGAPGAPGGKGDA
Theoretical DKGDTGPPGPQGLQGLPGTGGPPGENGKPGEPGPKGDAGAPGAPGGKGDA
560 570 580 590 600
BaP4H-SigN-rhCOL3a1 GAPGERGPPGLAGAPGLRGGAGPPGPEGGKGAAGPPGPPGAAGTPGLQGM
P4Hab-SigN-rhCOL3a1 GAPGERGPPGLAGAPGLRGGAGPPGPEGGKGAAGPPGPPGAAGTPGLQGM
P4Hab-BaP4H-SigN-rhCOL3a1 GAPGERGPPGLAGAPGLRGGAGPPGPEGGKGAAGPPGPPGAAGTPGLQGM
Theoretical GAPGERGPPGLAGAPGLRGGAGPPGPEGGKGAAGPPGPPGAAGTPGLQGM

```
                                610       620       630       640       650
BaP4H-SigN-rhCOL3a1          PGERGGLGSPGPKGDKGEPGGPGADGVPGKDGPRGPTGPIGPPGPAGQPG
P4Hab-SigN-rhCOL3a1          PGERGGLGSPGPKGDKGEPGGPGADGVPGKDGPRGPTGPIGPPGPAGQPG
P4Hab-BaP4H-SigN-rhCOL3a1    PGERGGLGSPGPKGDKGEPGGPGADGVPGKDGPRGPTGPIGPPGPAGQPG
Theoretical                  PGERGGLGSPGPKGDKGEPGGPGADGVPGKDGPRGPTGPIGPPGPAGQPG

                                660       670       680       690       700
BaP4H-SigN-rhCOL3a1          DKGEGGAPGLPGIAGPRGSPGERGETGPPGPAGFPGAPGQNGEPGGKGER
P4Hab-SigN-rhCOL3a1          DKGEGGAPGLPGIAGPRGSPGERGETGPPGPAGFPGAPGQNGEPGGKGER
P4Hab-BaP4H-SigN-rhCOL3a1    DKGEGGAPGLPGIAGPRGSPGERGETGPPGPAGFPGAPGQNGEPGGKGER
Theoretical                  DKGEGGAPGLPGIAGPRGSPGERGETGPPGPAGFPGAPGQNGEPGGKGER

                                710       720       730       740       750
BaP4H-SigN-rhCOL3a1          GAPGEKGEGGPPGVAGPPGGSGPAGPPGPQGVKGERGSPGGPGAAGFPGA
P4Hab-SigN-rhCOL3a1          GAPGEKGEGGPPGVAGPPGGSGPAGPPGPQGVKGERGSPGGPGAAGFPGA
P4Hab-BaP4H-SigN-rhCOL3a1    GAPGEKGEGGPPGVAGPPGGSGPAGPPGPQGVKGERGSPGGPGAAGFPGA
Theoretical                  GAPGEKGEGGPPGVAGPPGGSGPAGPPGPQGVKGERGSPGGPGAAGFPGA

                                760       770       780       790       800
BaP4H-SigN-rhCOL3a1          RGLPGPPGSNGNPGPPGPSGSPGKDGPPGPAGNTGAPGSPGVSGPKGDAG
P4Hab-SigN-rhCOL3a1          RGLPGPPGSNGNPGPPGPSGSPGKDGPPGPAGNTGAPGSPGVSGPKGDAG
P4Hab-BaP4H-SigN-rhCOL3a1    RGLPGPPGSNGNPGPPGPSGSPGKDGPPGPAGNTGAPGSPGVSGPKGDAG
Theoretical                  RGLPGPPGSNGNPGPPGPSGSPGKDGPPGPAGNTGAPGSPGVSGPKGDAG

                                810       820       830       840       850
BaP4H-SigN-rhCOL3a1          QPGEKGSPGAQGPPGAPGPLGIAGITGARGLAGPPGMPGPRGSPGPQGVK
P4Hab-SigN-rhCOL3a1          QPGEKGSPGAQGPPGAPGPLGIAGITGARGLAGPPGMPGPRGSPGPQGVK
P4Hab-BaP4H-SigN-rhCOL3a1    QPGEKGSPGAQGPPGAPGPLGIAGITGARGLAGPPGMPGPRGSPGPQGVK
Theoretical                  QPGEKGSPGAQGPPGAPGPLGIAGITGARGLAGPPGMPGPRGSPGPQGVK

                                860       870       880       890       900
BaP4H-SigN-rhCOL3a1          GESGKPGANGLSGERGPPGPQGLPGLAGTAGEPGRDGNPGSDGLPGRDGS
P4Hab-SigN-rhCOL3a1          GESGKPGANGLSGERGPPGPQGLPGLAGTAGEPGRDGNPGSDGLPGRDGS
P4Hab-BaP4H-SigN-rhCOL3a1    GESGKPGANGLSGERGPPGPQGLPGLAGTAGEPGRDGNPGSDGLPGRDGS
Theoretical                  GESGKPGANGLSGERGPPGPQGLPGLAGTAGEPGRDGNPGSDGLPGRDGS

                                910       920       930       940       950
BaP4H-SigN-rhCOL3a1          PGGKGDRGENGSPGAPGAPGHPGPPGPVGPAGKSGDRGESGPAGPAGAPG
P4Hab-SigN-rhCOL3a1          PGGKGDRGENGSPGAPGAPGHPGPPGPVGPAGKSGDRGESGPAGPAGAPG
P4Hab-BaP4H-SigN-rhCOL3a1    PGGKGDRGENGSPGAPGAPGHPGPPGPVGPAGKSGDRGESGPAGPAGAPG
Theoretical                  PGGKGDRGENGSPGAPGAPGHPGPPGPVGPAGKSGDRGESGPAGPAGAPG

                                960       970       980       990      1000
BaP4H-SigN-rhCOL3a1          PAGSRGAPGPQGPRGDKGETGERGAAGIKGHRGFPGNPGAPGSPGPAGQQ
P4Hab-SigN-rhCOL3a1          PAGSRGAPGPQGPRGDKGETGERGAAGIKGHRGFPGNPGAPGSPGPAGQQ
P4Hab-BaP4H-SigN-rhCOL3a1    PAGSRGAPGPQGPRGDKGETGERGAAGIKGHRGFPGNPGAPGSPGPAGQQ
Theoretical                  PAGSRGAPGPQGPRGDKGETGERGAAGIKGHRGFPGNPGAPGSPGPAGQQ

                               1010      1020      1030      1040      1050
BaP4H-SigN-rhCOL3a1          GAIGSPGPAGPRGPVGPSGPPGKDGTSGHPGPIGPPGPRGNRGERGSEGS
P4Hab-SigN-rhCOL3a1          GAIGSPGPAGPRGPVGPSGPPGKDGTSGHPGPIGPPGPRGNRGERGSEGS
P4Hab-BaP4H-SigN-rhCOL3a1    GAIGSPGPAGPRGPVGPSGPPGKDGTSGHPGPIGPPGPRGNRGERGSEGS
Theoretical                  GAIGSPGPAGPRGPVGPSGPPGKDGTSGHPGPIGPPGPRGNRGERGSEGS

                               1060      1070      1080      1090      1100
BaP4H-SigN-rhCOL3a1          PGHPGQPGPPGPPGAPGPCCGGVGAAAIAGIGGEKAGGFAPYYGYIPEAP
P4Hab-SigN-rhCOL3a1          PGHPGQPGPPGPPGAPGPCCGGVGAAAIAGIGGEKAGGFAPYYGYIPEAP
P4Hab-BaP4H-SigN-rhCOL3a1    PGHPGQPGPPGPPGAPGPCCGGVGAAAIAGIGGEKAGGFAPYYGYIPEAP
Theoretical                  PGHPGQPGPPGPPGAPGPCCGGVGAAAIAGIGGEKAGGFAPYYGYIPEAP

                               1110      1120
BaP4H-SigN-rhCOL3a1          RDGQAYVRKDGEWVFLSTFL
P4Hab-SigN-rhCOL3a1          RDGQAYVRKDGEWVFLSTFL
P4Hab-BaP4H-SigN-rhCOL3a1    RDGQAYVRKDGEWVFLSTFL
Theoretical                  RDGQAYVRKDGEWVFLSTFL
```

Fig. S3

(A)

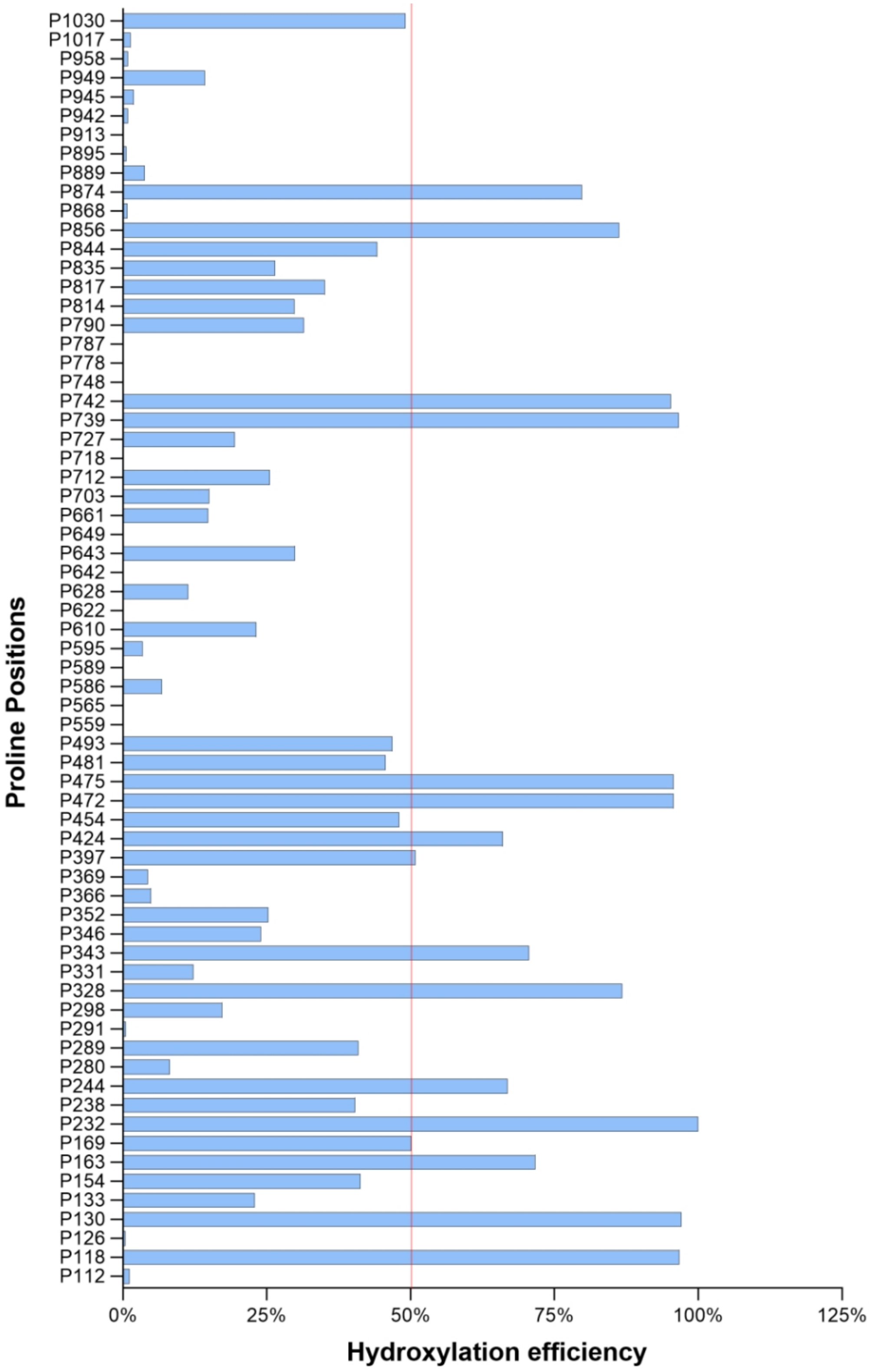

Proline Positions
P1030
P1017
P958
P949
P945
P942
P913
P895
P889
P874
P868
P856
P844
P835
P817
P814
P790
P787
P778
P748
P742
P739
P727
P718
P712
P703
P661
P649
P643
P642
P628
P622
P610
P595
P589
P586
P565
P559
P493
P481
P475
P472
P454
P424
P397
P369
P366
P352
P346
P343
P331
P328
P298
P291
P289
P280
P244
P238
P232
P169
P163
P154
P133
P130
P126
P118
P112
0%
25%
50%
75%
100%
125%
Hydroxylation efficiency

(B)

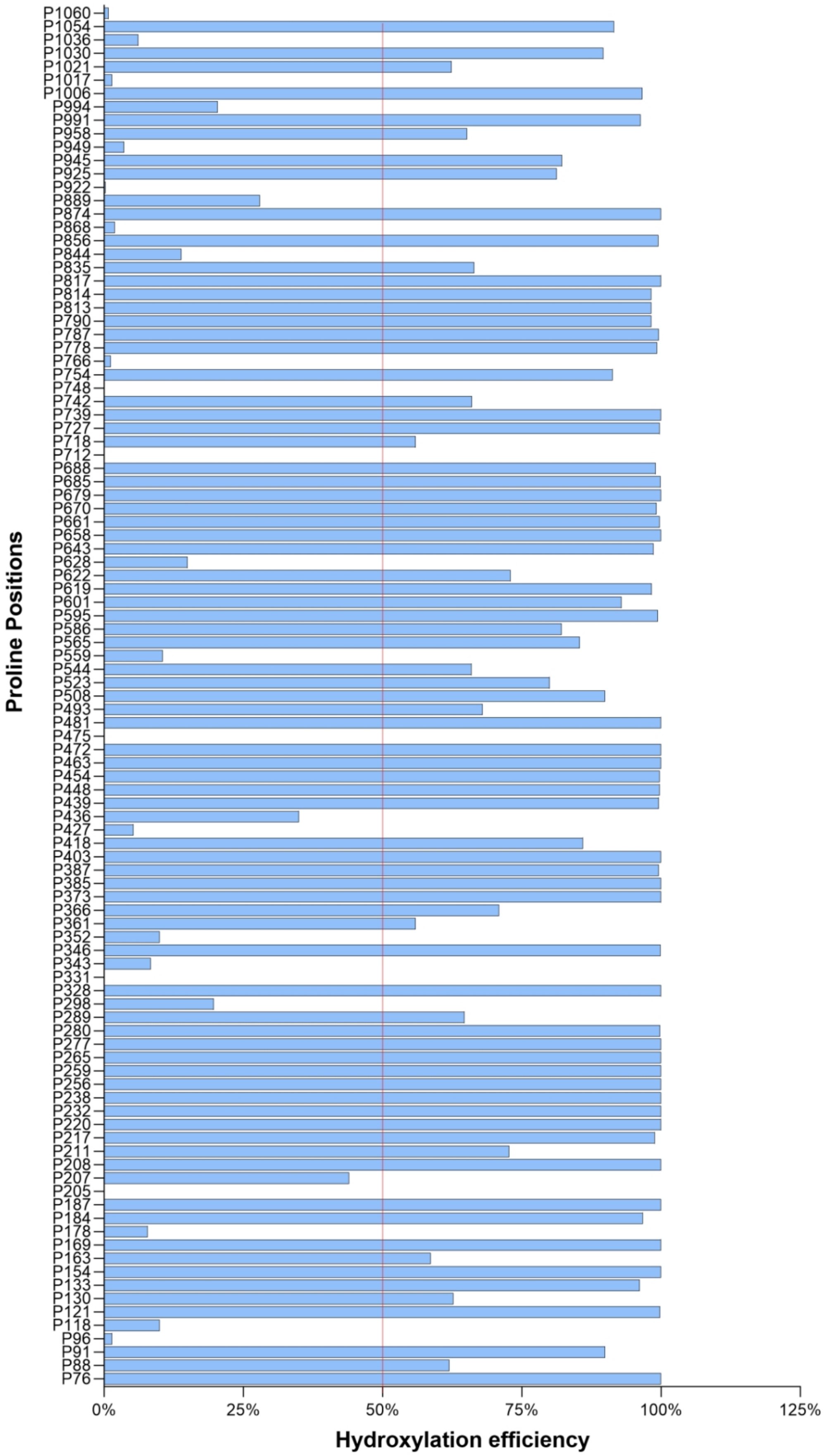

Proline Positions
P1060
P1054
P1036
P1030
P1021
P1017
P1006
P994
P991
P958
P949
P945
P925
P922
P889
P874
P868
P856
P844
P835
P817
P814
P813
P790
P787
P778
P766
P754
P748
P742
P739
P727
P718
P712
P688
P685
P679
P670
P661
P658
P643
P628
P622
P619
P601
P595
P586
P565
P559
P544
P523
P508
P493
P481
P475
P472
P463
P454
P448
P439
P436
P427
P418
P403
P387
P385
P373
P366
P361
P352
P346
P343
P331
P328
P298
P289
P280
P277
P265
P259
P256
P238
P232
P220
P217
P211
P208
P207
P205
P187
P184
P178
P169
P163
P154
P133
P130
P121
P118
P96
P91
P88
P76
0%
25%
50%
75%
100%
125%
Hydroxylation efficiency

(C)

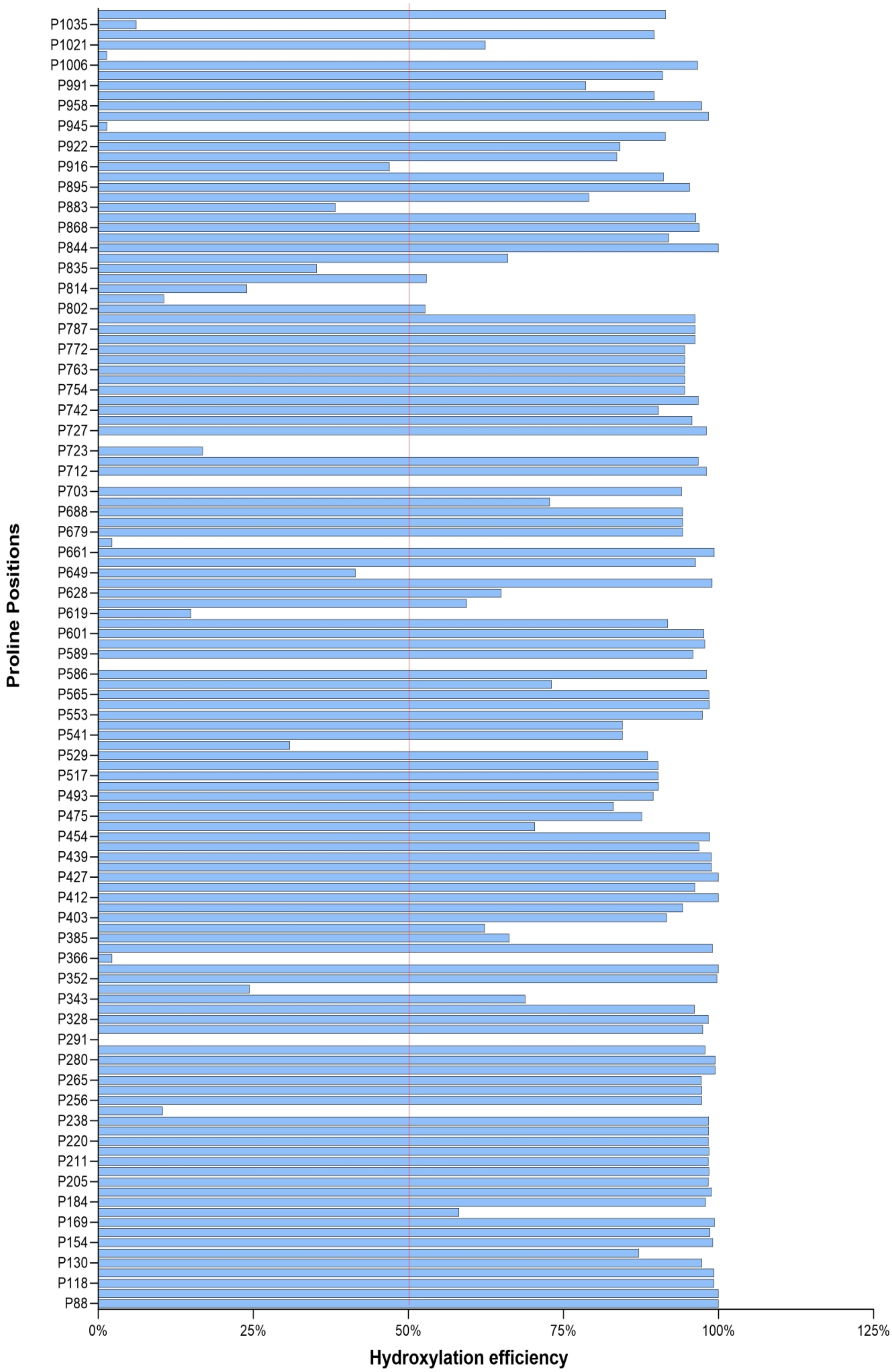

Proline Positions
P1035
P1021
P1006
P991
P958
P945
P922
P916
P895
P883
P868
P844
P835
P814
P802
P787
P772
P763
P754
P742
P727
P723
P712
P703
P688
P679
P661
P649
P628
P619
P601
P589
P586
P565
P553
P541
P529
P517
P493
P475
P454
P439
P427
P412
P403
P385
P366
P352
P343
P328
P291
P280
P265
P256
P238
P220
P211
P205
P184
P169
P154
P130
P118
P88
0%
25%
50%
75%
100%
125%
Hydroxylation efficiency

Table S1. Primers used in this study.

Primers	Sequence（5'→3'）
**Colony PCR**	
P4Hα-test-F	GGCTGAAGAGGATAAGTTGGAAC
P4Hα-test-R	CTCACCTCTACACAACATTTCGTAC
P4Hβ-test-F	CTTGGTGTGGTCATTGTAAGGC
P4Hβ-test-R	CTCTCAGCAGTCAATTCCTCAG
BaP4H-test-F	GACTGGTTCCAATTGACAAGCT
BaP4H-test-R	GACTGGTCCTGGTAGAAGTACTC
hCOL3α1 -test-F	GGTCCATGTTGTCAATACGACTC
hCOL3α1 -test-R	GTTGTCCATCAGAACCTCTTGCA
**Plasmid Construction**	
P4Hβ-AatII-F	GAAAAGTGCCACCTGACGTCAGATCTAACATCCAAAGACG
P4Hβ-AatII-R	TGGTTTCTTAGACGTCGGTCTCACTTAATCTTCTGTACTC
BaP4H-SphI-F	TTGGGCGCCATCTCCTTGATCTAACATCCAAAGACGAAAGGT
BaP4H-SphI-R	GCCGCAAGGAATGGTGTCTCACTTAATCTTCTGTACTCTGAAGAGGAG
αpre-hCOL3α1-F	ATTCGAAACGATGAGATTTCCTTCAATTTTTACTGCTGTTTTATTCGCAGCATCCTCCGCATTAGCTGGTCCATGTTGTCAATACGACTC
αpre-hCOL3α1-R	CAATGATGATGATGATGATGGTCGACTTACAAGAAGGTAGACAAGAAGACCCA
SPc1-hCOL3α1 -F	ATTCGAAACGATGTTCTCCTTCGTGGACCTTCGTTTGCTGCTACTGTTGGCAGCTACAGCCCTTTTGACTCATGGAGGTCCATGTTGTCAATACGACTC
SPc1-hCOL3α1 -R	CAATGATGATGATGATGATGGTCGACTTACAAGAAGGTAGACAAGAAGACCCA
Ost1-hCOL3α1 -F	ATTCGAAACGATGAGACAGGTTTGGTTCTCCTGGATCGTCGGTTTGTTCCTGTGCTTCTTCAACGTTTCTTCCGCCGGTCCATGTTGTCAATACGACTC
Ost1-hCOL3α1 -R	CAATGATGATGATGATGATGGTCGACTTACAAGAAGGTAGACAAGAAGACCCA
sgRNA-Δku70-F	ACAAATCAAAGCGGCCGCAAGATGCTGATGAGTCCGTGAGGACGAAACGAGTAAGCTCGTCCATCTTAGAGAATGTCAGTGGTTTTAGAGCTAGAAATAGCAAG
sgRNA-Δku70-R	GCATTCTGACATCCTCTTGAGTTTAAACTTAGAATCTAGCAAGACCGGTCTTCTCG
sgRNA-Chr1_4-F	CCTAAACAAATCAAAGCGGCCGCACTGCGCTGATGAGTCCGTG

	AGGACGAAACGAGTAAGCTCGTCCGCAGTGCGAGTGATGTCAAGTTTTAGAGCTAGAAATAGCA
sgRNA-Chr1_4-R	TCTGACATCCTCTTGAGTTTAAACTTAGAATCTAG
**Donor DNAs**	
Ku70-UP-F	CACGGGTGATTACTTGTTTACAT
ku70-UP-R	TGCGAGGAAAAAGTAAGGAACACGGGCTAAGTGTGAGAAGAAGAGA
ku70-DO-F	TCTCTTCTTCTCACACTTAGCCCGTGTTCCTTACTTTTTCCTCGCA
ku70-DO-R	CAATACCGATAAAGTGGTCAACT
0248-UP-F	ACTTACCACTGACATCGGCAC
0248-UP-Insert-R	GGACACCAAGACATTTCTACAAAAAAGATCCGCAGTGCGAGTGATGTCAAC
Insert-F	GTTGACATCACTCGCACTGCGGATCTTTTTTGTAGAAATGTCTTGGTGTCC
Insert-R	TCGTGTTGCACACGACGTTGGGTCTCACTTAATCTTCTGTACTCTGAAG
0249-DO-Insert-F	CTTCAGAGTACAGAAGATTAAGTGAGACCCAACGTCGTGTGCAACACGA
0249-DO-R	CGATCAGCACTCTCGTGATGAC
**RT-qPCR**	
hCOL3α1-F	GGTCCATGTTGTCAATACGACTC
hCOL3α1-R	GATTCACCATCCTTACCAGCAG
GAPDH-F	GCCAAGGTTGTCAACGACAC
GAPDH-R	TCAACAACGGAGACATCGAC

Table S2. Gene sequences used in this study

Genes	Sequences（5'→3'）
P4Hα	ATGATTTGGTACATTTTGATTATTGGTATTTTGTTGCCTCAATCCTTGGCTCATCCA<br>GGTTTCTTTACTTCCATTGGTCAAATGACTGATTTGATTCATACTGAAAAGGACTT<br>AGTTACCTCTCTGAAGGATTACATTAAGGCTGAAGAGGATAAGTTGGAACAAATT<br>AAGAAGTGGGCTGAAAAGCTTGATAGATTGACTTCAACCGCTACTAAGGACCCA<br>GAAGGTTTTGTTGGACATCCAGTTAACGCTTTTAAGTTGATGAAGAGACTCAACA<br>CTGAATGGTCAGAACTGGAAAATCTTGTTTTGAAGGATATGTCTGATGGTTTTATT<br>TCTAACTTAACTATTCAAAGACAATACTTTCCAAACGATGAAGATCAAGTTGGTG<br>CTGCTAAGGCTTTGTTGAGATTGCAAGATACTTACAACCTTGATACCGATACTATT<br>TCCAAGGGTAACTTGCCAGGTGTTAAGCATAAGTCCTTTTTGACCGCTGAAGATT<br>GTTTCGAACTGGGAAAGGTCGCTTATACTGAAGCTGATTACTACCATACTGAATT<br>GTGGATGGAACAAGCACTGAGACAACTGGATGAAGGTGAAATCTCTACTATTGAT<br>AAGGTTTCTGTCTTGGACTACCTTTCTTATGCTGTCTACCAACAAGGTGACTTGGA<br>TAAGGCTTTGTTGTTGACTAAGAAGCTTTTAGAGTTAGATCCAGAGCATCAAAGA<br>GCTAACGGTAACCTGAAGTACTTTGAGTACATTATGGCTAAGGAGAAGGACGTTA<br>ACAAGTCCGCCTCTGATGATCAGTCCGACCAAAAGACCACTCCAAAGAAGAAGG<br>GAGTTGCCGTCGATTACTTGCCCGAGAGACAGAAGTACGAAATGTTGTGTAGAGG<br>TGAGGGAATTAAGATGACTCCTCGTAGACAGAAGAAGTTGTTCTGTAGATATCAT<br>GATGGAAATAGAAATCCAAAGTTTATATTGGCTCCAGCTAAGCAAGAAGATGAA<br>TGGGATAAACCAAGAATTATTAGATTTCATGATATTATTTCTGATGCTGAAATTGA<br>AATCGTTAAGGATTTGGCCAAACCAAGACTTCGTAGAGCTACTATTTCCAACCCA<br>ATTACTGGTGACTTGGAAACTGTTCATTACAGGATCTCAAAGTCTGCTTGGTTGTC<br>TGGATACGAAAACCCAGTTGTTTCAAGAATTAACATGAGAATACAAGATTTGACT<br>GGATTGGATGTTTCTACCGCTGAAGAATTGCAAGTTGCCAACTATGGAGTTGGAG<br>GTCAATACGAACCACATTTTGATTTTGCTAGAAAGGATGAACCAGATGCTTTTAA<br>GGAATTGGGTACTGGTAACAGAATTGCTACCTGGTTGTTCTACATGTCTGATGTTT<br>CCGCTGGTGGTGCTACAGTTTTTCCTGAGGTTGGTGCTTCTGTTTGGCCTAAAAAG<br>GGTACTGCTGTCTTTTGGTACAACTTGTTTGCTTCCGGTGAAGGTGACTACTCTAC<br>TCGCCATGCTGCCTGTCCAGTGTTGGTTGGTAACAAGTGGGTTTCTAACAAGTGGT<br>TGCATGAAAGAGGACAAGAATTTCGTAGACCATGTACTTTGTCTGAACTTGAGTG<br>A

P4Hβ

ATGGACGCTCCTGAAGAAGAAGATCATGTTTTGGTTTTGAGAAAATCTAACTTTG
CTGAAGCTTTGGCTGCTCATAAGTACTTGTTGGTTGAATTTTATGCTCCTTGGTGT
GGTCATTGTAAGGCTTTAGCCCCTGAATACGCTAAGGCTGCTGGTAAATTGAAGG
CTGAAGGTAGTGAAATTAGATTGGCTAAAGTTGATGCTACTGAGGAATCTGATTT
GGCTCAACAATACGGAGTTAGAGGTTACCCAACTATTAAGTTTTTCAGAAACGGA
GATACTGCTTCTCCAAAGGAATACACTGCTGGTAGAGAAGCTGATGACATTGTTA
ACTGGTTGAAAAAACGTACCGGTCCAGCTGCTACTACTTTGCCTGATGGTGCTGC
TGCTGAATCTTTAGTTGAGTCCTCAGAAGTTGCTGTTATTGGTTTCTTTAAGGATG
TTGAATCTGATTCTGCTAAGCAATTTTTGCAAGCAGCTGAAGCTATTGATGATATT
CCTTTCGGTATTACTAGTAACAGTGATGTTTTTAGTAAGTATCAATTAGATAAGGA
TGGTGTTGTTTTGTTTAAGAAATTCGATGAAGGTAGAAACAATTTTGAAGGTGAG
GTTACTAAGGAGAACTTGTTGGACTTTATTAAGCATAACCAATTGCCATTGGTTAT
TGAGTTTACTGAACAAACTGCTCCTAAAATTTTTGGTGGTGAGATAAAGACTCAT
ATTTTGTTGTTTTTGCCAAAGTCTGTTAGTGATTACGATGGAAAGTTGTCTAACTT
TAAAACTGCTGCTGAATCTTTTAAAGGTAAAATTTTGTTTATTTTCATTGATTCTG
ATCATACTGATAACCAAAGAATCTTGGAATTTTTCGGTTTGAAGAAGGAGGAATG
TCCAGCTGTTAGACTGATTACTTTGGAGGAAGAAATGACTAAGTATAAGCCAGAA
TCTGAGGAATTGACTGCTGAGAGAATTACTGAATTTTGTCATAGATTCTTGGAAG
GAAAGATTAAGCCTCACTTGATGTCTCAAGAATTGCCAGAAGATTGGGATAAGCA
ACCAGTAAAAGTTTTGGTTGGTAAAAATTTTGAAGATGTTGCTTTTGATGAAAAG
AAAAACGTTTTTGTAGAATTTTACGCCCCTTGGTGTGGTCACTGTAAACAATTGGC
TCCTATTTGGGATAAATTGGGTGAAACTTACAAAGATCATGAAAACATTGTTATT
GCTAAAATGGATTCTACTGCTAATGAAGTTGAAGCTGTTAAAGTTCATTCTTTTCC
AACTTTGAAATTTTTCCCAGCTTCTGCTGATAGAACTGTTATTGATTACAACGGTG
AAAGAACTCTGGATGGTTTTAAGAAGTTTTTGGAATCTGGTGGTCAAGACGGTGC
TGGAGATGATGATGACTTGGAAGATTTGGAAGAAGCTGAGGAACCAGATATGGA
AGAAGATGATGATCAAAAGGCTGTTAAGGATGAATTGTAA

BaP4H

ATGATTTGGTACATTTTAATTATAGGTATTTTGTTGCCACAATCTTTAGCTATGAC
TAATAATAACCAAATAGGTGAAAATAAGGAACAAACAATTTTTGATCATAAAGG
TAATGTAATTAAGACTGAAGATAGAGAAATTCAAATAATTTCTAAATTTGAAGAA
CCATTGATAGTAGTTTTAGGTAACGTTTTGTCTGATGAAGAATGTGATGAATTGAT
TGAATTGAGTAAATCTAAGTTAGCTAGATCTAAAGTTGGTAGTAGTAGAGATGTT
AATGATATTAGAACTTCATCAGGTGCATTTCTTGATGATAATGAATTAACTGCTAA
GATTGAAAAAAGAATTAGTAGTATAATGAATGTACCAGCATCACATGGTGAAGG
ATTACATATTTTAAACTATGAAGTTGATCAACAATATAAAGCACATTATGATTACT
TCGCTGAGCACTCCCGTTCCGCTGCCAACAACAGGATTTCCACCCTGGTCATGTAC
TTGAACGACGTGGAGGAGGGTGGTGAGACCTTCTTCCCAAAGCTGAACTTGTCCG
TCCACCCCAGAAAGGGTATGGCTGTTTACTTCGAGTACTTCTACCAGGACCAGTC
CCTGAACGAGCTGACCCTGCACGGTGGTGCTCCCGTCACTAAGGGTGAGAAGTGG
ATCGCTACCCAGTGGGTTAGAAGGGGAACCTACAAGGAGTTCCATGATGAATTGT
AA

rhCOL3 α1	ATGTCATTTGTGCAGAAAGGTAGCTGGTTACTCCTAGCACTTTTGCATCCCACGAT<br>CATTCTGGCTGGTCCATGTTGTCAATACGACTCCTACGACGTTAAGTCCGGTGTTG<br>CTGTTGGTGGTTTGGCTGGTTATCCAGGTCCAGCTGGTCCACCAGGTCCACCTGGA<br>CCTCCAGGCACTTCTGGTCATCCTGGTTCTCCAGGATCTCCAGGTTATCAAGGACC<br>ACCAGGTGAACCAGGACAAGCTGGTCCTTCTGGACCTCCTGGACCACCTGGTGCT<br>ATTGGACCATCTGGTCCTGCTGGTAAGGATGGTGAATCCGGTAGACCAGGTAGAC<br>CCGGTGAAAGAGGTTTACCAGGTCCTCCAGGTATTAAGGGTCCAGCAGGTATTCC<br>AGGTTTCCCTGGTATGAAGGGTCATAGAGGTTTCGACGGTAGAAACGGTGAAAA<br>GGGTGAAACTGGTGCTCCTGGATTGAAGGGTGAGAATGGTTTGCCAGGTGAAAA<br>CGGTGCACCTGGTCCAATGGGTCCAAGAGGTGCTCCAGGTGAGAGAGGTAGACC<br>TGGATTGCCTGGTGCAGCTGGTGCTAGAGGTAATGATGGTGCAAGAGGTTCTGAT<br>GGACAACCCGGTCCTCCAGGTCCTCCTGGTACTGCTGGTTTTCCAGGTTCACCAGG<br>TGCTAAGGGCGAAGTTGGACCAGCAGGTTCCCCTGGATCTAATGGTGCCCCAGGT<br>CAAAGAGGTGAGCCCGGTCCACAAGGTCATGCTGGTGCACAAGGCCCACCAGGA<br>CCACCAGGCATTAACGGTTCTCCAGGTGGTAAAGGTGAAATGGGTCCTGCCGGAA<br>TTCCAGGCGCTCCAGGTTTGATGGGAGCTAGAGGCCCTCCTGGTCCAGCCGGTGC<br>CAATGGTGCTCCCGGTTTGAGAGGTGGTGCTGGTGAGCCAGGTAAGAATGGTGCC<br>AAAGGTGAGCCTGGACCAAGAGGCGAGAGAGGTGAAGCTGGTATACCTGGTGTT<br>CCCGGTGCAAAAGGCGAAGATGGTAAAGATGGATCTCCTGGCGAACCCGGTGCT<br>AACGGACTTCCAGGTGCCGCTGGTGAAAGGGGTGCACCAGGTTTTAGAGGACCA<br>GCCGGTCCTAACGGTATTCCTGGTGAAAAAGGACCTGCTGGCGAAAGAGGTGCCC<br>CTGGACCTGCCGGACCTAGAGGTGCAGCAGGCGAACCTGGTAGAGATGGTGTTCC<br>AGGCGGACCAGGTATGAGAGGTATGCCAGGCTCTCCTGGTGGCCCAGGATCTGAT<br>GGTAAACCAGGACCTCCAGGATCACAAGGTGAGTCTGGTAGGCCAGGACCTCCT<br>GGTCCTTCAGGTCCTAGAGGTCAACCAGGTGTTATGGGTTTTCCTGGACCTAAGG<br>GAAATGATGGCGCTCCTGGTAAAAATGGTGAACGTGGTGGCCCCGGTGGTCCCGG<br>ACCTCAGGGCCCACCTGGAAAGAATGGCGAAACTGGACCTCAAGGTCCACCTGG<br>TCCTACTGGTCCCGGTGGTGATAAGGGTGATACAGGCCCTCCAGGACCTCAGGGA<br>TTGCAAGGTTTACCTGGAACCGGTGGTCCACCTGGCGAAAATGGTAAGCCAGGTG<br>AACCCGGTCCTAAGGGTGATGCTGGCGCCCCAGGCGCACCTGGCGGCAAAGGTG<br>ATGCCGGTGCACCCGGTGAGAGGGGTCCTCCAGGATTGGCTGGTGCTCCAGGACT<br>AAGAGGTGGTGCAGGTCCTCCTGGTCCTGAAGGTGGTAAAGGCGCTGCAGGTCCT<br>CCAGGACCACCTGGCGCTGCTGGAACTCCAGGATTACAGGGAATGCCAGGTGAG<br>CGTGGTGGATTAGGATCACCAGGACCTAAAGGTGACAAGGGCGAACCAGGCGGC<br>CCTGGTGCTGATGGTGTCCCTGGAAAAGATGGTCCCAGAGGACCAACAGGACCTA<br>TCGGTCCACCAGGACCTGCCGGTCAACCTGGTGACAAAGGTGAAGGTGGTGCACC<br>TGGACTGCCAGGTATTGCTGGTCCTAGGGGTAGTCCAGGCGAAAGGGGAGAGAC<br>TGGCCCACCAGGTCCTGCAGGTTTTCCCGGTGCTCCTGGTCAGAACGGTGAGCCT<br>GGTGGAAAAGGCGAACGTGGCGCTCCCGGTGAGAAAGGTGAAGGCGGTCCTCCA<br>GGTGTTGCTGGACCTCCTGGTGGTAGTGGTCCAGCCGGACCTCCAGGTCCACAGG<br>GTGTTAAGGGTGAACGTGGATCTCCAGGCGGTCCCGGCGCAGCTGGTTTCCCAGG<br>CGCTAGAGGTTTGCCCGGACCTCCTGGCTCTAATGGAAATCCTGGTCCTCCAGGT<br>CCATCAGGTTCACCAGGTAAAGATGGTCCTCCTGGACCAGCAGGTAATACTGGTG<br>CTCCAGGTTCTCCTGGTGTTTCTGGTCCAAAAGGTGATGCAGGTCAGCCAGGCGA

	AAAAGGATCCCCAGGTGCTCAGGGCCCTCCAGGTGCTCCCGGACCATTAGGAATT<br>GCTGGTATTACAGGTGCCAGAGGTTTAGCTGGCCCACCTGGTATGCCAGGTCCAA<br>GAGGATCACCTGGTCCTCAAGGCGTAAAAGGTGAATCAGGCAAACCAGGCGCAA<br>ACGGTCTTAGCGGAGAAAGAGGCCCACCTGGACCACAAGGTTTACCAGGCTTAG<br>CTGGAACTGCTGGCGAGCCTGGTCGTGATGGAAATCCAGGTTCAGATGGATTGCC<br>AGGCAGAGATGGATCACCCGGTGGCAAGGGTGACAGAGGCGAAAACGGCTCTCC<br>CGGCGCACCAGGCGCTCCCGGCCATCCAGGTCCACCAGGTCCTGTCGGACCAGCA<br>GGTAAATCAGGTGATAGAGGTGAATCTGGACCCGCAGGACCCGCTGGCGCACCT<br>GGACCAGCCGGATCTCGTGGTGCCCCTGGTCCTCAGGGTCCACGTGGCGACAAGG<br>GTGAGACTGGCGAGAGGGGAGCTGCTGGTATCAAAGGACACAGAGGATTCCCAG<br>GTAACCCTGGCGCACCCGGATCTCCCGGTCCAGCTGGCCAACAAGGTGCTATTGG<br>TAGTCCAGGTCCTGCTGGCCCAAGGGGTCCAGTCGGTCCAAGTGGCCCTCCTGGA<br>AAAGACGGTACTTCTGGACATCCAGGTCCTATTGGCCCTCCAGGTCCAAGGGGTA<br>ATAGAGGTGAAAGAGGATCTGAAGGATCTCCTGGTCATCCAGGACAACCCGGAC<br>CACCTGGTCCTCCAGGCGCTCCTGGACCATGTTGTGGTGGTGTTGGTGCTGCTGCT<br>ATTGCTGGAATTGGTGGTGAGAAGGCTGGTGGTTTTGCTCCTTACTACGGTTACAT<br>CCCAGAGGCTCCAAGAGACGGTCAAGCTTACGTTAGAAAGGACGGTGAGTGGGT<br>CTTCTTGTCTACCTTCTTGTAA
GADPH	ATGGCTATCACTGTCGGTATTAACGGTTTCGGACGTATTGGACGTCTCGTTCTGAG<br>AGTCGCTCTTTCCAGAGCTGACATCAAGGTTGTTGCTATCAACGACCCATTCATTG<br>CTCCAGAATACGCTGCTTACATGTTCAAGTACGACTCTACCCACAAGGCTTACAA<br>GGGTGAGGTTTCTGCCAGCGGCAACAAGATCAACATTGACGGTAACGAAATCAC<br>CGTTTTCCAAGAGAGAGACCCTGTCAACATCCCATGGGGTAAGGCTGGTGTCGAC<br>TACGTCATTGAGTCCACCGGTGTTTTCACCACTTTGGAGGGTGCCCAAAAGCACA<br>TCGACGCCGGTGCCAAGAAGGTCGTCATCACTGCTCCATCCAAGGATGCTCCAAT<br>GTTCGTTGTCGGTGTCAACGAGGAGAAATACACTTCTGACTTGAACATTGTCTCC<br>AATGCTTCTTGTACTACCAACTGTTTGGCTCCATTGGCCAAGGTTGTCAACGACAC<br>TTTCGGAATTGAGTCCGGTTTGATGACCACCGTCCACTCCATGACCGCCACTCAA<br>AAGACCGTTGACGGTCCATCCCACAAGGACTGGAGAGGTGGTAGAACCGCTTCTG<br>GTAACATCATTCCATCTTCCACTGGTGCTGCTAAGGCCGTCGGTAAGGTTATTCCA<br>GAATTGAACGGTAAGCTGACCGGTTTGGCTTTCCGTGTCCCAACCGTCGATGTCTC<br>CGTTGTTGACTTGACCGTCAACTTGAAGAAGGAGACTACCTACGAGGAGATCAAG<br>TCTGTTATCAAGGCTGCTTCCGAGGGTAAGCTCAAGGGTGTTTTGGGTTACACTG<br>AAGATGCCGTTGTCTCTTCTGACTTCTTGGGTGACGAGAGATCCTCCATCTTCGAC<br>GCTTCTGCCGGTATTCAATTGACTCCATCTTTCGTCAAGCTGATCTCTTGGTACGA<br>CAACGAGTACGGTTACTCCACCAGAGTCGTCGACTTGTTGCAACACGTTGCTAAG<br>GCTTAA
HACI	ATGGAAATGACCGATTTCGAATTAACGTCTAATAGTCAGTCCAACTTGGCAATAC<br>CAACAAATTTCAAATCCACGCTGCCACCCCGGAAGAGGGCCAAAACGAAGGAAG<br>AGAAGGAACAACGTCGAATTGAAAGGATCCTACGAAACAGACGCGCGGCTCATC<br>AAAGCCGGGAAAAAAAGAGGTTACATTTACAATATCTAGAGCGCAAGTGTTCATT<br>ATTGGAGAACTTGTTGAATAGTGTGAATCTAGAGAAACTAGCTGACCATGAAGAC<br>GCCTTGACTTGCTCCCACGATGCTTTTGTCGCGTCTCTCGACGAGTACCGTGACTT<br>CCAATCAACTCGGGGGGCGAGCCTTGACACTCGTGCATCGAGTCACTCATCGTCG

GACACGTTTACACCCTCACCCCTTAACTGCACAATGGAACCTGCCACGCTGTCGC
CGAAAAGCATGAGAGATTCTGCGTCTGATCAGGAGACTAGCTGGGAGCTGCAGA
TGTTTAAGACCGAGAATGTTCCTGAAAGTACCACCCTCCCTGCAGTAGATAACAA
CAATCTTTTCGACGCCGTCGCGTCCCCACTTGCTGACCCGCTGTGTGATGATATTG
CAGGCAACAGTCTCCCGTTTGATAACAGCATAGATCTCGACAACTGGCGCAATCC
CGAGGCTCAGTCGGGACTGAATTCTTTTGAATTAAATGATTTTTTCATCACATCAC
ATGATGAGTTGTAG

BiP

ATGCTGTCGTTAAAACCATCTTGGCTGACTTTGGCGGCATTAATGTATGCCATGCT
ATTGGTCGTAGTGCCATTTGCTAAACCTGTTAGAGCTGACGATGTCGAATCTTATG
GAACAGTGATTGGTATCGATTTGGGTACCACGTACTCTTGTGTCGGTGTGATGAA
GTCGGGTCGTGTAGAAATTCTTGCTAATGACCAAGGTAACAGAATCACTCCTTCC
TACGTTAGTTTCACTGAAGACGAGAGACTGGTTGGTGATGCTGCTAAGAACTTAG
CTGCTTCTAACCCAAAAAACACCATCTTTGATATTAAGAGATTGATCGGTATGAA
GTATGATGCCCCAGAGGTCCAAAGAGACTTGAAGCGTCTTCCTTACACTGTCAAG
AGCAAGAACGGCCAACCTGTCGTTTCTGTCGAGTACAAGGGTGAGGAGAAGTCTT
TCACTCCTGAGGAGATTTCCGCCATGGTCTTGGGTAAGATGAAGTTGATCGCTGA
GGACTACTTAGGAAAGAAAGTCACTCATGCTGTCGTTACCGTTCCAGCCTACTTC
AACGACGCTCAACGTCAAGCCACTAAGGATGCCGGTCTCATCGCCGGTTTGACTG
TTCTGAGAATTGTGAACGAGCCTACCGCCGCTGCCCTTGCTTACGGTTTGGACAA
GACTGGTGAGGAAAGACAGATCATCGTCTACGACTTGGGTGGAGGAACCTTCGAT
GTTTCTCTGCTTTCTATTGAGGGTGGTGCTTTCGAGGTTCTTGCTACCGCCGGTGA
CACCCACTTGGGTGGTGAGGACTTTGACTACAGAGTTGTTCGCCACTTCGTTAAG
ATTTTCAAGAAGAAGCATAACATTGACATCAGCAACAATGATAAGGCTTTAGGTA
AGCTGAAGAGAGAGGTCGAAAAGGCCAAGCGTACTTTGTCTTCCCAGATGACTAC
CAGAATTGAGATTGACTCTTTCGTCGACGGTATCGACTTCTCTGAGCAACTGTCTA
GAGCTAAGTTTGAGGAGATCAACATTGAATTATTCAAGAAGACACTGAAACCAGT
TGAACAAGTCCTCAAAGACGCTGGTGTCAAGAAATCTGAAATTGATGACATTGTC
TTGGTTGGTGGTTCTACCAGAATTCCAAAGGTTCAACAATTATTGGAGGATTACTT
TGACGGAAAGAAGGCTTCTAAGGGAATTAACCCAGATGAAGCTGTCGCATACGG
TGCTGCTGTTCAGGCTGGTGTTTTGTCTGGTGAGGAAGGTGTCGATGACATCGTCT
TGCTTGATGTGAACCCCCTAACTCTGGGTATCGAGACTACTGGTGGCGTTATGACT
ACCTTAATCAACAGAAACACTGCTATCCCAACTAAGAAATCTCAAATTTTCTCCA
CTGCTGCTGACAACCAGCCAACTGTGTTGATTCAAGTTTATGAGGGTGAGAGAGC
CTTGGCTAAGGACAACAACTTGCTTGGTAAATTCGAGCTGACTGGTATTCCACCA
GCTCCAAGAGGTACTCCTCAAGTTGAGGTTACTTTTGTTTTAGACGCTAACGGAAT
TTTGAAGGTCTCTGCCACCGATAAGGGAACTGGAAAATCCGAGTCCATCACCATC
AACAATGATCGTGGTAGATTGTCCAAGGAGGAGGTTGACCGTATGGTTGAAGAG
GCCGAGAAGTACGCCGCTGAGGATGCTGCACTAAGAGAAAAGATTGAGGCTAGA
AACGCTCTGGAGAACTACGCTCATTCCCTTAGGAACCAAGTTACTGATGACTCTG
AAACCGGGCTTGGTTCTAAATTGGACGAGGACGACAAAGAGACATTGACAGATG
CCATCAAAGATACCCTAGAGTTCTTGGAAGACAACTTCGACACCGCAACCAAGGA
AGAATTAGACGAACAAAGAGAAAAGCTTTCCAAGATTGCTTACCCAATCACTTCT
AAGCTATACGGTGCTCCAGAGGGTGGTACTCCACCTGGTGGTCAAGGTTTTGACG
ATGATGATGGAGACTTTGACTACGACTATGACTATGATCATGATGAGTTGTAG

FKBP22

GCTTTGATCCCAGAGCCTGAGGTCAAGATTGAAGTTTTGCAGAAGCCATTCATCT
GTCACAGAAAGACTAAGGGTGGCGACTTGATGCTGGTCCACTACGAGGGATACCT
GGAGAAGGACGGTTCCTTGTTCCACTCTACCCACAAGCACAACAACGGTCAACCA
ATCTGGTTCACCTTGGGTATCTTGGAGGCTCTGAAGGGTTGGGACCAGGGTTTGA
AGGGTATGTGTGTCGGTGAGAAGAGAAAGTTGATTATCCCACCAGCCCTGGGATA
CGGAAAGGAGGGTAAAGGAAAGATCCCACCAGAGTCTACCTTGATTTTCAACATC
GACCTGTTGGAGATTAGAAACGGACCACGTTCCCACGAGTCCTTCCAGGAGATGG
ACTTGAACGACGACTGGAAGTTGTCCAAGGACGAGGTCAAGGCTTACTTGAAGA
AGGAGTTCGAGAAGCACGGTGCTGTTGTCAACGAGTCTCACCACGACGCCTTGGT
CGAGGACATTTTCGATAAGGAGGACGAGGACAAGGACGGTTTCATCTCCGCTAG
AGAGTTCACCTACAAGCACGATGAGTTGTAA

Hsp47

ATGAGATCTTTGTTGTTGTTGTCTGCTTTCTGTTTGTTGGAGGCTGCCTTGGCTGCT
GAAGTTAAGAAGCCAGCTGCTGCTGCCGCTCCAGGTACTGCTGAAAAGTTGTCCC
CTAAGGCAGCTACCTTAGCTGAAAGATCAGCTGGATTGGCTTTCTCTTTGTACCAA
GCTATGGCTAAGGATCAAGCTGTTGAAAACATCTTGGTTTCCCCAGTTGTTGTTGC
TTCTTCCTTGGGTTTGGTTTCTTTGGGTGGAAAGGCCACTACCGCTTCTCAGGCTA
AGGCTGTCTTGTCTGCTGAACAACTGAGAGATGAGGAAGTTCACGCTGGTTTGGG
TGAATTGTTGAGATCTTTATCTAACTCCACTGCTAGAAACGTTACTTGGAAATTGG
GTTCAAGATTGTACGGTCCATCCTCTGTTTCATTCGCTGACGATTTCGTTAGATCT
TCTAAGCAACATTACAACTGTGAGCACTCTAAGATCAACTTCAGAGATAAGAGAT
CCGCTTTGCAATCTATTAACGAATGGGCTGCTCAAACCACTGATGGTAAATTGCC
TGAAGTCACTAAGGATGTTGAAAGAACTGATGGTGCTCTATTGGTTAACGCTATG
TTCTTCAAGCCACACTGGGACGAGAAGTTTCACCATAAGATGGTTGACAACAGAG
GATTCATGGTTACTAGATCTTACACTGTTGGTGTTATGATGATGCACAGAACTGGT
CTGTACAACTACTACGATGACGAGAAGGAAAAGTTGCAAATCGTTGAAATGCCAT
TGGCTCACAAGCTTTCCTCCTTGATTATTCTGATGCCACATCACGTCGAGCCACTG
GAGAGATTGGAGAAGTTGTTGACTAAGGAACAACTGAAGATTTGGATGGGAAAG
ATGCAAAAGAAGGCTGTTGCCATTTCCCTTCCAAAGGGTGTTGTTGAAGTTACCC
ATGACTTGCAAAAGCACTTGGCTGGTTTGGGTTTGACCGAGGCTATTGATAAGAA
TAAGGCTGATCTATCTAGAATGTCTGGTAAAAAGGACTTGTACTTGGCTTCTGTTT
TTCACGCTACTGCTTTCGAGTTGGATACTGATGGTAACCCATTCGATCAAGATATC
TATGGTAGAGAGGAATTGAGATCTCCTAAGTTGTTCTACGCTGATCACCCATTCAT
CTTTCTGGTTAGAGATACTCAATCTGGTTCTTTGTTGTTCATTGGTAGACTTGTTAG
ACCAAAGGGTGACAAGATGCATGATGAATTGTAA

	ATGAGGATAGTAAGGAGCGTAGCTATCGCAATAGCCTGTCATTGTATAACAGCGT
	TAGCAAACCCTCAAATCCCTTTTGACGGCAACTACACCGAGATCATCGTGCCAGA
	TACCGAAGTTAACATCGGACAGATTGTAGATATTAACCACGAAATAAAACCCAA
	ACTGGTGGAACTGGTCAACACAGACTTCTTCAAATATTACAAATTAAACCTATGG
	AAACCATGTCCGTTTTGGAATGGTGATGAGGGATTCTGCAAGTATAAGGATTGCT
	CTGTCGACTTTATCACTGATTGGTCTCAGGTGCCTGATATCTGGCAACCAGACCAA
	TTGGGTAAGCTTGGAGATAACACGGTACATAAGGATAAGGGCCAAGATGAAAAT
	GAGCTGTCCTCAAATGATTATTGCGCTTTGGATAAAGACGACGATGAAGATTTAG
	TATATGTCAATTTGATTGATAACCCTGAAAGATTCACCGGTTATGGTGGTCAGCA
	ATCTGAATCTATTTGGACTGCGGTCTATGATGAGAACTGTTTCCAGCCGAATGAA
	GGATCACAATTGGGTCAAGTTGAAGACCTCTGTTTGGAGAAACAGATCTTTTACC
	GATTGGTTTCTGGTTTGCATTCTAGTATCTCCACCCACCTCACAAACGAATATCTG
	AATTTGAAAAATGGAGCATACGAACCAAATTTGAAACAGTTCATGATCAAAGTTG
	GGTATTTTACTGAAAGAATTCAAAACTTACATCTCAATTATGTCCTTGTATTGAAG
EROI	TCACTAATAAAGCTACAAGAATACAATGTTATCGACAATCTACCTCTCGATGACT
	CTTTGAAAGCTGGTCTTAGCGGTTTAATATCTCAAGGAGCACAGGGTATTAACCA
	GAGCTCTGATGATTATCTATTTAACGAGAAGGTTCTTTTCCAAAATGACCAAAAT
	GATGATTTGAAAAATGAATTCCGTGACAAATTCCGCAACGTGACTAGATTAATGG
	ATTGTGTCCATTGCGAGAGATGCAAATTATGGGGAAAATTGCAAACTACAGGGTA
	CGGGACTGCATTGAAGATTCTATTTGATTTGAAGAATCCTAATGACTCCATCAATT
	TAAAGAGAGTTGAGTTAGTTGCTCTAGTCAACACATTCCATAGATTGTCCAAATC
	TGTTGAAAGCATTGAAAACTTTGAAAAACTATATAAGATTCAACCGCCAACGCAG
	GATCGTGCATCAGCGTCGTCCGAATCCTTAGGCCTTTTCGATAACGAAGATGAAC
	AAAATCTCCTCAACTCGTTTTCGGTTGATCAGGCAGTCATTTCATCGAAAGAGGC
	ACCAGAAGAAATCAAAAGCAAACCTGTTGGAAAAGCCGCATATAAACAAAACAG
	TTGTCCATCATTGGGTTCAAAATCTATCAAAGAAGCATTCCATGAAGAACTTCAC
	GCATTTATTGATGCAATTGGATTTATATTGAACTCTTACAGGACTTTGCCCAAGCT
	GTTGTACACACTTTTCCTCGTTAAATCATCTGAATTATGGGACATTTTCATTGGCA
	CTCAAAGGCACCGAGATACCACATATAGAGTAGACTTGCATGATGAGTTGTAA
	ATGACAGCATACACGGACGTCGTAAACGACTTGAGTAGAGAATTATCCTTGAAGA
	AACCCAAAGATGTGATCCAATTCTGTGCGGACTACTTCAACAATAAGTTAGCAGA
	GGATCCTGCCAGACAACAACCATCAACAGCAACAGCCCCCTCTTCACGCCCTGCT
	CAGACTGCTTCAGGCGCACCTCTGTTTAAAGACCAGTTTGGAGGTGATCCTCATG
	AACCATTGTCTCATCACCCTCATCGTGGTAGCATTTTTAAGGACTCATTCACTCAT
	CCTGAAGACCCTCAAAGTAATAAAATAAGGCCTTTGGATAATGCCTCCCCTGGCT
	CAGGATTTGCCGCACATCTTGCTAAGGTTAAGTTCAACCCAGAGAGACGTATCTC
Bcy1	TGTGAGTGCCGAATCTGTGAATCCAGAAATTTTTGCTACCAGTTCTTGGCAGCCAC
	CTGTCCACGACCTGAGTGATGAGCAACTGCAACGTTTAAACAACATTGTCAGAAA
	GAGTTTCCTGTTTAGCCAGCTAGAAGATGAAGCACTACGAACAGTGATATATGCC
	TTGGAAGAGAAGAAAGTGCCACGCGGAACAGAGATAATCAAACAAGGCGACGA
	AGGTGATTACTTTTACGTTTTGGAAAGCGGTGAGGTCACATTTGTTGTCAATGGA
	GAGACTAAAGGCCAAGGAAAGTCTGGGTCAACCTTTGGTGAACTTGCACTGATGT
	ATAACTCGCCGCGGGCGGCCACCGTGATTTCTAGTCAGGACTGTGTTCTTTGGGC
	ACTTGATCGTATGACGTTTAGAAGAATTTTACTTGAAGGTACAGCCAAGAGACGT

GCGATGTATGATGGGTTTCTCAAGGATGTTCCAGTGTTACGCTGTCTCAACTCCTA
TGAACGTAATAAGCTCGCTGATGCTTTAGAATCTGAGACTTACAACAAAGGCGAC
ACGATCATCCGTGAAGGCGAACCAGGAGAAAACTTTTATTTTATTGAACAAGGTG
AAGCTGAAGTGTTTAAAGAAGGTGAAGGACATATGGCAACATTGGGCAAAGGTG
ATTACTTTGGCGAACTCGCTCTGTTGAACGACCTTCCACGTCAAGCTACTGTCGTT
GCTAAAAGTGACGTGATCAAGGTTGTTACCCTAGATAAGAATGGATTCCAGAGAT
TGCTTGGGCCAGCAATTGAGGTTCTCAAGCTGCAAGATCCCACCAAGATCCATGA
TGAGTTGTAG

Table S3. Details of genomic editing sites used in this study.

Genomic sites	sgRNA sequence (N20+NGG)（5'→3'）
Ku70	CATCTTAGAGAATGTCAGTG AGG
PAS_Chr1-4	CGCAGTGCGAGTGATGTCAA CGG

Table S4. The copy number of the rhCOL3α1 gene in different recombinant strains.

Recombinant strains	Ct value		Copy numbers
	GAPDH	*rhCOLα1*	
GS-SigN-rhCOL3α1	19.757 ± 0.033	19.644 ± 0.014	1.37
	19.987 ± 0.050	18.602± 0.069	3.18
	20.017 ± 0.022	18.260 ± 0.056	4.07
	19.170 ± 0.049	16.769 ± 0.089	6.22
	20.169 ± 0.026	17.413 ± 0.012	7.56
	20.250 ± 0.037	17.330 ± 0.042	8.79
	19.615 ± 0.050	16.452 ± 0.017	10.31
GS-BaP4H-SigN-rhCOL3α1	20.175 ± 0.016	18.03 ± 0.039	2.32
	20.35 ± 0.023	16.97 ± 0.093	5.44
	20.35 ± 0107	16.79 ± 0.166	6.14
	19.62 ± 0.021	15.75 ± 0.055	7.59
	20.22 ± 0.003	16.21 ± 0.072	8.45
	19.76 ± 0.011	15.54 ± 0.034	9.65
GS-P4Hαβ-SigN-rhCOL3α1	19.21 ± 0.063	16.68 ± 0.041	2.76
	19.67 ± 0.101	16.64 ± 0.008	3.89
	20.08 ± 0.013	16.67 ± 0.069	5.07
	19.59 ± 0.033	15.61 ± 0.083	7.26
	19.27 ± 0.066	15.11 ± 0.014	8.08
	19.97 ± 0.013	15.62 ± 0.027	9.38
GS-P4Hαβ-BaP4H-SigN-rhCOL3α1	19.36 ± 0.050	19.46 ± 0.043	0.56
	20.35 ± 0.034	18.41 ± 0.114	2.18
	20.35 ± 0.031	17.33 ± 0.041	4.36
	19.62 ± 0.016	16.02 ± 0.055	6.23
	20.22 ± 0.007	16.21 ± 0.073	8.30
	19.76 ± 0.041	15.59 ± 0.069	9.07
GS-P4Hαβ-BaP4H-SPc1-rhCOL3α1	19.50 ± 0.120	18.41 ± 0.045	2.21
	18.72 ± 0.11	16.71 ± 0.069	4.02
	19.06 ± 0.089	16.52 ± 0.082	5.74
	18.97 ± 0.022	16.02 ± 0.034	7.53
	17.75 ± 0.048	14.54 ± 0.010	8.78
	18.81 ± 0.012	15.28 ± 0.054	11.04
GS-P4Hαβ-BaP4H-αpre-rhCOL3α1	21.36 ± 0.046	21.24 ± 0.026	1.56
	21.97 ± 0.005	20.79 ± 0.012	3.17
	21.01 ± 0.013	19.52 ± 0.033	4.06
	21.26 ± 0.056	19.10 ± 0.081	6.25
	21.53 ± 0.010	19.16 ± 0.064	7.16
	21.31 ± 0.006	18.63 ± 0.033	8.86
GS-P4Hαβ-BaP4H-Ost1-rhCOL3α1	21.52 ± 0.047	20.83 ± 0.019	2.15
	21.33 ± 0.13	19.97 ± 0.110	3.34

	21.74 ± 0.075	20.08 ± 0.098	4.07
	20.32 ± 0.032	18.12 ± 0.044	5.76
	21.52 ± 0.044	18.79 ± 0.028	8.20
	20.98 ± 0.022	18.16 ± 0.073	8.67
