# Supplementary material for: Enhanced biosynthesis of full-length triple-helical human type III collagen in engineered Komagataella phaffii
Source: Appl Microbiol Biotechnol. 2026 Aug 29;110:300. doi: 10.1007/s00253-026-13996-7 (PMC13646244; doi:10.1007/s00253-026-13996-7)
Supplement: Supplementary file 2 [file 253_2026_13996_MOESM2_ESM.docx]

***Supplementary materials***

**Enhanced biosynthesis of full-length triple-helical human type III collagen in engineered *Komagataella phaffii***

Zhijian Ni ^1,2^·Xiaolu Yu ^2^·Qi Da ^2^·Xiaoxi Wu ^2^·ZhiDa Wei ^2^·Yulei Hu ^2^·Yao Xiao ^4^·Chunting Ye ^2^·Chunlin Deng ^1,3,^*

**Fig. S1.** Growth profiles of recombinant strains harboring different rhCOL3α1 copy numbers. Biomass (OD600) was measured after 48 h of methanol-induced shake-flask fermentation.

**Fig. S2.** Distribution of hydroxyproline residues across the rhCOL3α1 chain in recombinant strains co-expressing collagen with different P4Hs, as identified by LC-MS/MS. Hydroxylated proline sites are color-coded as follows: yellow, 67 sites in strain GS-BaP4H-SigN-rhCOL3α1 (10-copy); blue, 103 sites in strain GS-P4Hαβ-SigN-rhCOL3α1 (8-copy); and red, 128 sites in strain GS-P4Hαβ-BaP4H-SigN-rhCOL3α1 (8-copy).

**Fig. S3.** Site-specific hydroxylation efficiency (defined as the percentage of Hyp at each modified proline residue) of the rhCOL3α1 chain in recombinant strains co-expressing collagen with P4Hs, determined by LC-MS/MS. **(A)** Strain GS-BaP4H-SigN-rhCOL3α1 (10-copy): 16 of 67 modified sites exhibited a Hyp content exceeding 50%. **(B)** Strain GS-P4Hαβ-SigN-rhCOL3α1 (8-copy): 77 of 103 modified sites exhibited a Hyp content exceeding 50%. **(C)** Strain GS-P4Hαβ-BaP4H-SigN-rhCOL3α1 (8-copy): 108 of 125 modified sites exhibited a Hyp content exceeding 50%.

**Fig. S1**

**Fig. S2**

**Fig. S3**

**(A)**

**(B)**

**Table S1.** Primers used in this study.

**Table S2** Details of genomic editing sites used in this study.

| Genomic sites | sgRNA sequence (N20+NGG)（5'→3'） |
| --- | --- |
| Ku70 | CATCTTAGAGAATGTCAGTG AGG |
| PAS_Chr1-4 | CGCAGTGCGAGTGATGTCAA CGG |

**Table S3** The copy number of the rhCOL3α1 gene in different recombinant strains.

| Recombinant strains | Ct value | | Copy numbers |
| --- | --- | --- | --- |
|  | *GAPDH* | *rhCOLα1* |  |
| GS-SigN-rhCOL3α1 | 19.757 ± 0.033 | 19.644 ± 0.014 | 1.37 |
|  | 19.987 ± 0.050 | 18.602± 0.069 | 3.18 |
|  | 20.017 ± 0.022 | 18.260 ± 0.056 | 4.07 |
|  | 19.170 ± 0.049 | 16.769 ± 0.089 | 6.22 |
|  | 20.169 ± 0.026 | 17.413 ± 0.012 | 7.56 |
|  | 20.250 ± 0.037 | 17.330 ± 0.042 | 8.79 |
|  | 19.615 ± 0.050 | 16.452 ± 0.017 | 10.31 |
| GS-BaP4H-SigN-rhCOL3α1 | 20.175 ± 0.016 | 18.03 ± 0.039 | 2.32 |
|  | 20.35 ± 0.023 | 16.97 ± 0.093 | 5.44 |
|  | 20.35 ± 0107 | 16.79 ± 0.166 | 6.14 |
|  | 19.62 ± 0.021 | 15.75 ± 0.055 | 7.59 |
|  | 20.22 ± 0.003 | 16.21 ± 0.072 | 8.45 |
|  | 19.76 ± 0.011 | 15.54 ± 0.034 | 9.65 |
| GS-P4Hαβ-SigN-rhCOL3α1 | 19.21 ± 0.063 | 16.68 ± 0.041 | 2.76 |
|  | 19.67 ± 0.101 | 16.64 ± 0.008 | 3.89 |
|  | 20.08 ± 0.013 | 16.67 ± 0.069 | 5.07 |
|  | 19.59 ± 0.033 | 15.61 ± 0.083 | 7.26 |
|  | 19.27 ± 0.066 | 15.11 ± 0.014 | 8.08 |
|  | 19.97 ± 0.013 | 15.62 ± 0.027 | 9.38 |
| GS-P4Hαβ-BaP4H-SigN-rhCOL3α1 | 19.36 ± 0.050 | 19.46 ± 0.043 | 0.56 |
|  | 20.35 ± 0.034 | 18.41 ± 0.114 | 2.18 |
|  | 20.35 ± 0.031 | 17.33 ± 0.041 | 4.36 |
|  | 19.62 ± 0.016 | 16.02 ± 0.055 | 6.23 |
|  | 20.22 ± 0.007 | 16.21 ± 0.073 | 8.30 |
|  | 19.76 ± 0.041 | 15.59 ± 0.069 | 9.07 |
| GS-P4Hαβ-BaP4H-SPc1-rhCOL3α1 | 19.50 ± 0.120 | 18.41 ± 0.045 | 2.21 |
|  | 18.72 ± 0.11 | 16.71 ± 0.069 | 4.02 |
|  | 19.06 ± 0.089 | 16.52 ± 0.082 | 5.74 |
|  | 18.97 ± 0.022 | 16.02 ± 0.034 | 7.53 |
|  | 17.75 ± 0.048 | 14.54 ± 0.010 | 8.78 |
|  | 18.81 ± 0.012 | 15.28 ± 0.054 | 11.04 |
| GS-P4Hαβ-BaP4H-αpre-rhCOL3α1 | 21.36 ± 0.046 | 21.24 ± 0.026 | 1.56 |
|  | 21.97 ± 0.005 | 20.79 ± 0.012 | 3.17 |
|  | 21.01 ± 0.013 | 19.52 ± 0.033 | 4.06 |
|  | 21.26 ± 0.056 | 19.10 ± 0.081 | 6.25 |
|  | 21.53 ± 0.010 | 19.16 ± 0.064 | 7.16 |
|  | 21.31 ± 0.006 | 18.63 ± 0.033 | 8.86 |
| GS-P4Hαβ-BaP4H-Ost1-rhCOL3α1 | 21.52 ± 0.047 | 20.83 ± 0.019 | 2.15 |
|  | 21.33 ± 0.13 | 19.97 ± 0.110 | 3.34 |
|  | 21.74 ± 0.075 | 20.08 ± 0.098 | 4.07 |
|  | 20.32 ± 0.032 | 18.12 ± 0.044 | 5.76 |
|  | 21.52 ± 0.044 | 18.79 ± 0.028 | 8.20 |
|  | 20.98 ± 0.022 | 18.16 ± 0.073 | 8.67 |

## Uncropped gels and images

**Fig. 1. (B)** SDS-PAGE analysis of recombinant P4Hs in neutral cell lysates after 48 h of shake-flask fermentation. Lane M: Marker; lane 1: GS-P4Hαβ; lane 2: GS-P4Hαβ-BaP4H; lane 3: *K. phaffii* GS115 (control); lane 4: GS-BaP4H. Arrows indicate the P4H α-subunit (~60 kDa) and β-subunit (~55 kDa); the BaP4H band (~27 kDa) was not detected.

**Fig. 1. (C)** Reducing SDS-PAGE of recombinant collagen in acidified cell lysates after 48 h of shake-flask fermentation. The arrow indicates the rhCOL3α1 chain (~130 kDa).

**Fig. 1.(D)** Non-reducing SDS-PAGE of recombinant collagen in acidified lysates from high-copy-number strains, with or without pepsin treatment. Lane M: Marker; lane hCOL3: natural human type III collagen standard (2 μg loaded). Arrows indicate the trimer band (> 300 kDa) and pepsin (~40 kDa).

**Fig. 2.** Expression of rhCOL3 procollagen using alternative signal sequences. **(A)** Non-reducing SDS-PAGE of acidified lysates from high-copy-number strains after 48 h of shake-flask fermentation, with or without pepsin treatment. Lane M: Marker; lane hCOL3: natural human type III collagen standard (2 μg loaded). Arrows indicate the trimer band (> 300 kDa) and pepsin (~40 kDa).

**Fig. 3.** Enhancement of rhCOL3 procollagen expression via chaperone co-expression. **(B)** Non-reducing SDS-PAGE of acidified lysates from the indicated strains after 48 h of shake-flask fermentation. Lane M: Marker; lane hCOL3: natural human type III collagen standard (2 μg loaded); lane 1: control strain (GSΔku70-P4Hαβ-BaP4H-Ost1-rhCOL3α1); lanes 2–7: strains co-expressing FKBP22, ERO1, HAC1, BiP, HSP47, and BCY1, respectively. Arrows indicate the trimer band (> 300 kDa).

**Fig. 4.** Production of rhCOL3 procollagen in 5-L bioreactors. **(A)** Non-reducing SDS-PAGE of acidified cell lysates after 60 h of methanol induction in fed-batch fermentation. Lane M: Marker; lane hCOL3: natural human type III collagen standard (2 μg loaded); Group 1: GSΔku70-P4Hαβ-BaP4H-Ost1-rhCOL3α1-Bcy1; Group 2: GS-P4Hαβ-BaP4H-SigN-rhCOL3α1 (8-copy).

**Fig. 5.** Purification of triple-helical rhCOL3. **(A)** Non-reducing SDS-PAGE of samples from successive purification steps. (a) Supernatant of cell lysate; (b) lysate supernatant after pepsin digestion; (c) purified rhCOL3.

**Fig. 6. (E)** Self-assembly properties of triple-helical rhCOL3.(c) TEM images of collagen fibrils at 500 nm, 200 nm, and 50 nm scale.
